# Chronic Administration of Exogenous Lactate Increases Energy Expenditure during Exercise through Activation of Skeletal Muscle Energy Utilization Capacity in Mice

**DOI:** 10.3390/metabo14040220

**Published:** 2024-04-13

**Authors:** Inkwon Jang, Sunghwan Kyun, Deunsol Hwang, Taeho Kim, Kiwon Lim, Hun-Young Park, Sung-Woo Kim, Jisu Kim

**Affiliations:** 1 Laboratory of Exercise and Nutrition, Department of Sports Medicine and Science in Graduate School, Konkuk University, Seoul 05029, Republic of Korea; inkwon555@konkuk.ac.kr (I.J.); y10345@konkuk.ac.kr (S.K.); hds49@konkuk.ac.kr (D.H.); qwer92224@konkuk.ac.kr (T.K.); exercise@konkuk.ac.kr (K.L.); parkhy1980@konkuk.ac.kr (H.-Y.P.); kswrha@konkuk.ac.kr (S.-W.K.); 2 Physical Activity and Performance Institute (PAPI), Konkuk University, Seoul 05029, Republic of Korea; 3 Department of Physical Education, Konkuk University, Seoul 05029, Republic of Korea

**Keywords:** chronic exogenous lactate, exercise training, metabolic during exercise, energy substrate utilization, body composition, metabolic regulator

## Abstract

We compared the effects of chronic exogenous lactate and exercise training, which influence energy substrate utilization and body composition improvements at rest and during exercise, and investigated the availability of lactate as a metabolic regulator. The mice were divided into four groups: CON (sedentary + saline), LAC (sedentary + lactate), EXE (exercise + saline), and EXLA (exercise + lactate). The total experimental period was set at 4 weeks, the training intensity was set at 60–70% VO_2_max, and each exercise group was administered a solution immediately after exercise. Changes in the energy substrate utilization at rest and during exercise, the protein levels related to energy substrate utilization in skeletal muscles, and the body composition were measured. Lactate intake and exercise increased carbohydrate oxidation as a substrate during exercise, leading to an increased energy expenditure and increased protein levels of citrate synthase and malate dehydrogenase 2, key factors in the TCA(tricarboxylic acid) cycle of skeletal muscle. Exercise, but not lactate intake, induced the upregulation of the skeletal muscle glucose transport factor 4 and a reduction in body fat. Hence, chronic lactate administration, as a metabolic regulator, influenced energy substrate utilization by the skeletal muscle and increased energy expenditure during exercise through the activation of carbohydrate metabolism-related factors. Therefore, exogenous lactate holds potential as a metabolic regulator.

## 1. Introduction

Physical inactivity causes various metabolic issues collectively called “the diseasome of physical inactivity” [1]. This is because physical inactivity is highly correlated with various metabolic diseases [2] such as type 2 diabetes [3], cardiovascular disease [4], cancer [5], and depression [6]. Therefore, preventing metabolic diseases by identifying the mediators linking physical activity and metabolic improvement has become a research hotspot.

Exercise resolves physical inactivity and regulates metabolism [7]. Myokines secreted from the skeletal muscles interact with endocrine organs to mediate physiological metabolic adaptations, such as energy homeostasis [8], insulin response [9], and anti-inflammatory regulation [10]. They improve health and extend the lifespan by lowering the risk of various chronic diseases. Therefore, it is essential to investigate the molecular mediators that activate myokines and metabolism, which provide beneficial adaptations to exercise and skeletal muscle [11,12].

Lactate, a myokine secreted by skeletal muscles, plays a central role in metabolism during exercise [13]. In the past, lactate has been considered only an indicator of fatigue [14], but it is also reused as an energy source through gluconeogenesis [15,16] and the Cori cycle [17], and it initiates metabolic change reactions through interactions with other tissues as a signaling substance. Lactate holds promise as an “exerkine” that can regulate and activate metabolism [13,18].

Acute exogenous lactate IP injection of 3 g/kg in mice increased blood lactate concentration to 20 mmol/L and significantly increased p-AMPK expression, which is related to oxidative metabolism in skeletal muscles [19]. Additionally, exogenous lactate treatment increased TGF-β2 expression in adipocytes in cells and animals, which improved glucose metabolism and increased fatty acid oxidation, but this effect was not observed when lactate production was inhibited [20]. Moreover, our previous study reported that acute exogenous lactate administration at rest increased the expression of factors related to glycogen synthesis and fat oxidation in skeletal muscles, and its administration before exercise increased carbohydrate utilization during exercise [21,22].

Hence, exogenous lactate has the potential to regulate the metabolic activation of skeletal muscle and adipose tissue, as well as altering energy substrate utilization. However, the metabolic effects of the chronic intake of exogenous lactate remain unknown. A previous study investigated the phosphorylation levels of proteins related to oxidative metabolism, but it was an acute study and did not identify changes in energy substrate utilization [19]. Additionally, lactate induces the expression of TGF-β2, causing metabolic improvement, but metabolic changes were not confirmed after direct treatment with exogenous lactate [20]. Our previous study also investigated changes in skeletal muscle metabolism through the acute administration of exogenous lactate at rest and before exercise, but did not confirm the effect of chronic lactate administration on metabolic changes [21,22].

To determine the availability of lactate as a supplement, comparing the effects of chronic lactate administration and exercise training (a non-pharmacological approach to improve metabolism) on energy substrate use is essential at rest and during exercise, and so too is assessing body composition changes. Furthermore, investigating the expression levels of energy utilization-related proteins in skeletal muscle is vital. Therefore, we investigated the effects of four weeks of exogenous lactate administration, exercise training, and combined treatment on energy substrate utilization at rest and during exercise, changes in body composition (fat and fat-free mass), and key energy utilization-related protein expression in skeletal muscles. Finally, we have evaluated the potential of lactate as a metabolic regulator, and suggest directions for future research based on our study findings.

## 2. Materials and Methods

### 2.1. Animal Care

Six-week-old male Institute of Cancer Research mice (Orient Bio Inc., Seongnam, Republic of Korea) were acclimatized to the laboratory environment for 1 week (*n* = 28). All mice were housed in standard plastic cages at 23–24 °C with 45–50% humidity and a 12 h dark/light cycle (lights on from 19:00 to 07:00), and provided with a standard commercial diet (5L79, Orient Bio Inc., Seongnam, Republic of Korea) and water ad libitum.

### 2.2. Study Design

**Figure 1:**
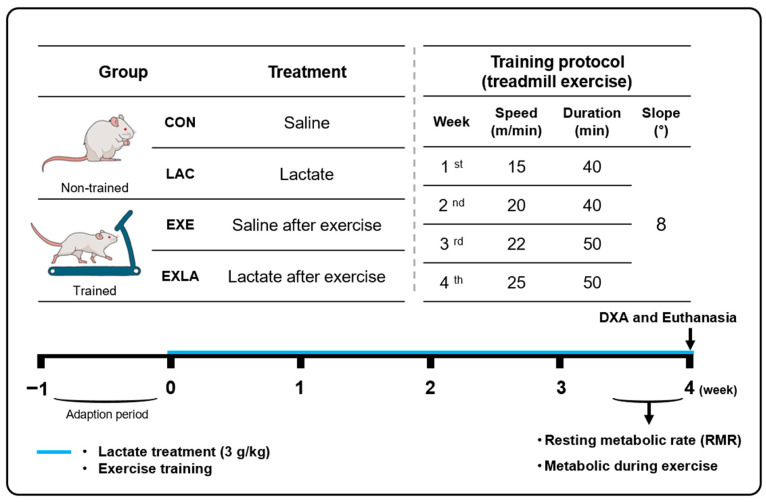
Schematic representation of the experimental design and procedure. Seven-week-old male ICR mice were used for this experiment. After the one-week adaption period, mice were randomly divided into four groups (*n* = 7 per group), and each treatment was performed for 4 weeks. Lactate groups were administered sodium lactate (3 g/kg), and control groups were orally administered an equal amount of saline. Exercise groups were administered each solution immediately after every exercise. CON, sedentary and saline administration; LAC, sedentary and lactate administration; EXE, exercise training and saline administration; EXLA, exercise training and lactate administration; RMR, resting metabolic rate; DXA, dual-energy X-ray absorptiometry.

The experimental design and process are illustrated in Figure 1. Seven-week-old male, acclimatized mice were randomly divided into four groups (*n* = 7 per group): CON (sedentary and saline administration), LAC (sedentary and lactate administration), EXE (exercise training and saline administration), and EXLA (exercise and lactate administration). The lactate group was orally administered sodium lactate (3 g/kg, 195-05965, Wako Chemical, Osaka, Japan). Equal amounts of saline were administered orally to the CON group. The exercise training groups were administered each solution immediately after each exercise session.

Exercise training was conducted on a treadmill five times per week for four weeks. Exercise intensity was set to 60–70% of VO_2_ max (15–25 m/min) for 40–50 min. The speed and time was increased each week considering exercise adaption. The slope of the treadmill was fixed at 8°. Mice were euthanized under anesthesia using 10 μL/g of 1.25% avertin and dissected 48 h after the last exercise and lactate treatment. All tissues were weighed and stored at −80 °C immediately after dissection.

### 2.3. Metabolic Analysis at Rest and during Exercise

To investigate the effects of 4 weeks of lactate intake and exercise training on energy metabolism, measurements were conducted using a metabolic analyzer. Separated metabolic chambers were used for 24 h to measure resting energy metabolism. Energy metabolism during exercise was measured using separate metabolic treadmill chambers at a constant speed for 1 h (speed, 20 m/min; slope, 8°). All measurements were conducted 1 d after training to minimize the effects of acute exercise. The metabolic chambers were constructed using the open-circuit method and the volume of each chamber was 3 L. The average flow rate in each chamber was 3 L/min. Respiratory gases were analyzed using a mass spectrometer (ARCO-2000; ARCO System, Chiba, Japan) and a switching system (ARCO-2000-GS-8; ARCO System, Chiba, Japan), allowing the spectrometer to sample the gas from each chamber. An acrylic tube was connected to each chamber to control the air volume. Based on the measured oxygen uptake (VO_2_), carbon dioxide production (VCO_2_), and respiratory exchange rate (RER), carbohydrate oxidation (CO), fat oxidation (FO), and energy expenditure (EE) were calculated [22].

### 2.4. Body Composition Analysis

The mice were scanned using DXA (iNSiGHT VET, Osteosys, Seoul, Republic of Korea) under anesthesia before dissection. Body composition was calculated using an embedded DXA algorithm [23].

### 2.5. Immunoblotting Analysis

Gastrocnemius muscles were homogenized using a TissueRuptor (QIAGEN, Hilden, Germany) containing 800 μL of protein extraction buffer (EzRIPA Lysis kit, WSE07420, ATTO, Tokyo, Japan). Whole lysates were centrifuged at 20,000× *g* at 4 °C for 15 min. The top layer (lipid) was removed, and the clear supernatants were transferred to a new tube. Protein concentration was determined using a Pierce™ BCA Protein Assay Kit (23225, Thermo Fisher Scientific, Waltham, MA, USA). Proteins were denatured by heating at 55 or 100 °C for 5–10 min. Total protein (20 μg per lane) was separated using 10, 12, and 14% SDS-PAGE at 60 V for 30 min, followed by 100 V for 130 min, and then transferred to polyvinylidene difluoride membranes (Millipore, Billerica, MA, USA) at 100 V for 2 h. The membranes were blocked for 1 h at 25 °C in 5% non-fat dried milk (NFDM, F141511, Cellconic, Hanam, Republic of Korea) in 0.1% PBS-T. Later they were incubated overnight at 4 °C in primary antibodies in 3% NFDM in 0.1% PBS-T, and subsequently incubated for 90 min at 25 °C in horseradish peroxidase-conjugated secondary antibodies in 3% NFDM in 0.1% PBS-T. Immunodetection was performed using ECL™ Prime Western blotting detection reagent (GERPN2232; Cytiva, Marlborough, MA, USA). All images showing the results of the quantitative analysis were assessed using ImageJ software 1.8. Information on the antibodies used is provided in Supplementary Table S1.

### 2.6. Statistical Analysis

All data were analyzed using IBM SPSS Statistics 28 software. All graphs were created using GraphPad Prism 10.2 software. All data, except food intake, were checked for normality of distribution and analyzed using one-way analysis of variance (ANOVA). Post hoc tests were conducted using Tukey’s HSD. Food intake was calculated with mean differences among groups using the Kruskal–Wallis test, a non-parametric analysis. Statistical methods were not used to predetermine the sample sizes, which have been expressed as mean ± standard deviation (SD). Statistical significance was set at *p* < 0.05.

## 3. Results

### 3.1. Administration of Exogenous Lactate Increases Carbohydrate Utilization as an Energy Substrate during Exercise, Leading to Increased Energy Expenditure

**Figure 2:**
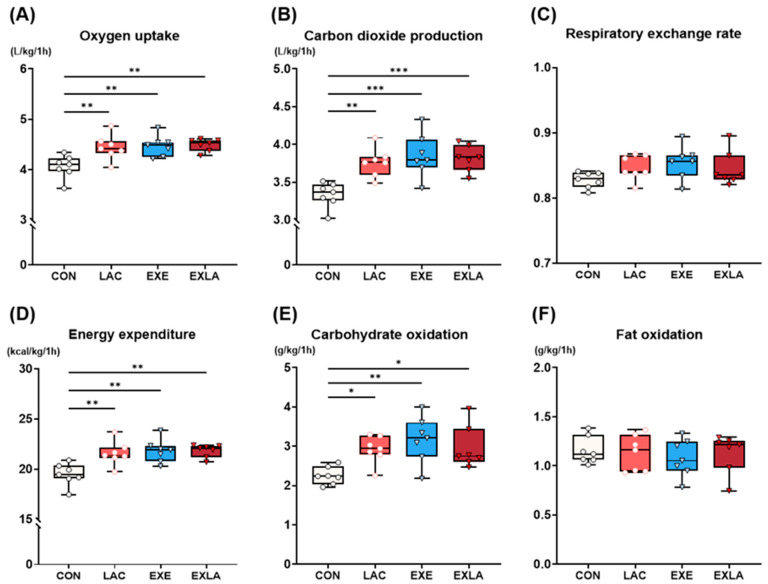
Administration of exogenous lactate increases carbohydrate utilization as an energy substrate during exercise, leading to increased energy expenditure. Metabolic rate during exercise was measured on each independent treadmill chamber at a constant speed for 1 h (speed—20 m/min, slope—8°). The data were compiled for 1 h, and the integrated values were (**A**) VO_2_, (**B**) VCO_2_, (**C**) RER, (**D**) EE, (**E**) CO, and (**F**) FO, while RER represents the 1 h average value. A one-way ANOVA was performed, and Tukey HSD was used as a post hoc test (*n* = 7 per group). VO_2_, oxygen uptake; VCO_2_, carbon dioxide production; RER, respiratory exchange rate; EE, energy expenditure; CO, carbohydrate oxidation; FO, fat oxidation. * *p* < 0.05, ** *p* < 0.01, *** *p* < 0.001 vs. CON. Data are presented as mean ± standard deviation.

To determine the effects of 4 weeks of lactate intake and exercise training on energy substrate use and consumption, metabolic rate was measured at a constant speed for 1 h (Figure 2). Compared with the control group, oxygen uptake (VO_2_; vs. LAC, *p* = 0.01; EXE, *p* = 0.006; EXLA, *p* = 0.004) and carbon dioxide production (VCO_2_; vs. LAC, *p* = 0.004; EXE, *p* < 0.001; EXLA, *p* < 0.001) were significantly increased in all groups (Figure 2A,B). Energy expenditure was also significantly increased in all groups compared to the control group (vs. LAC, *p* = 0.005; EXE, *p* = 0.002; EXLA, *p* = 0.002), which was achieved by significantly increasing carbohydrate oxidation (vs. LAC: *p* = 0.048; EXE: *p* = 0.005; EXLA: *p* = 0.034) rather than fat oxidation as an energy substrate (Figure 2D,E). The combination of lactate and exercise had no additional effect on metabolism during exercise. Considering these results, exercise as well as independent lactate intake increases the oxidation of carbohydrates during exercise, and also increases energy expenditure, leading to metabolic improvement.

### 3.2. Exercise Training Induces Positive Changes in Body Composition but Not in Body Weight 

**Figure 3:**
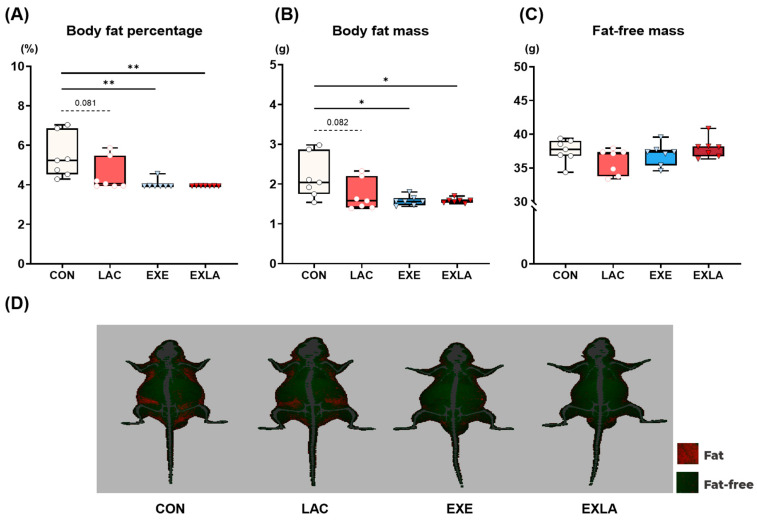
Exercise training induces positive improvements in body composition but not in body weight. After the 4-week experiment, body composition was measured using DXA under anesthesia before dissection. (**A**) Body fat percentage. (**B**) Body fat mass. (**C**) Fat-free mass. (**D**) Representative DXA images. A one-way ANOVA was performed, and Tukey HSD was used as a post hoc test (*n* = 7 per group). * *p* < 0.05, ** *p* < 0.01 vs. CON. DXA, dual-energy X-ray absorptiometry. Data are presented as mean ± standard deviation.

We used DXA to determine the effects of 4 weeks of lactate intake and exercise training on body composition (Figure 3). There were no significant differences in body weight or skeletal muscle mass between the groups. However, exercise training significantly lowered both body fat mass (EXE: *p* = 0.017; EXLA: *p* = 0.017) and body fat percentage (EXE: *p* = 0.005; EXLA: *p* = 0.003) compared to the control group (Figure 3C,D). Additionally, there was no significant difference in body fat improvement in the independent lactate intake group compared to that in the control group, but body fat tended to be significantly lower (LAC: *p* = 0.08; Figure 3C,D). Hence, it was confirmed that exercise training did not increase skeletal muscle but reduced body fat, leading to positive changes in body composition.

### 3.3. Administration of Exogenous Lactate Activates Oxidative Metabolism in Skeletal Muscle

**Figure 4:**
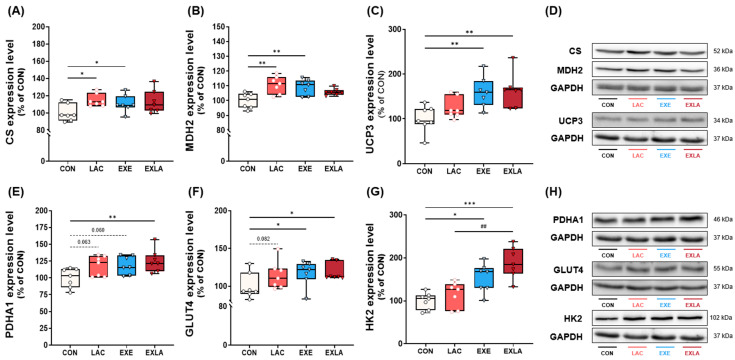
Administration of exogenous lactate activates oxidative metabolism in skeletal muscle. Protein expression levels of the gastrocnemius muscle: (**A**) CS, (**B**) MDH2, (**C**) UCP3, (**E**) PDHA1, (**F**) GLUT4, and (**G**) HK2. (**D**,**H**) Western blot images. A one-way ANOVA was performed, and Tukey HSD was used as a post hoc test (*n* = 7 per group). CS, citrate synthase; MDH2, malate dehydrogenase 2; UCP3, uncoupling protein 3; PDHA1, pyruvate dehydrogenase E1 subunit alpha 1; GLUT4, glucose transporter 4; HK2, hexokinase 2; GAPDH, glyceraldehyde-3-phosphate dehydrogenase. * *p* < 0.05, ** *p* < 0.01, *** *p* < 0.001 vs. CON; ## *p* < 0.01 vs. LAC. Data are presented as mean ± standard deviation.

To understand the molecular changes that occur due to exogenous lactate intake and exercise training, we examined the expression of citrate synthase (CS), malate dehydrogenase 2 (MDH2), uncoupling protein 3 (UCP3), pyruvate dehydrogenase E1 subunit alpha 1 (PDHA1), glucose transporter 4 (GLUT4), hexokinase 2 (HK2), oxidative phosphorylation system (OxPhos complexes), carnitine palmitoyltransferase 1B (CPT1B), adenosine monophosphate-activated protein kinase alpha (AMPKα), and peroxisome proliferator-activated receptor-gamma coactivator 1 alpha (PGC1α) proteins involved in energy substrate utilization and skeletal muscle activation. Independent lactate intake and exercise training showed that CS (vs. LAC: *p* = 0.023; vs. EXE: *p* = 0.021) and MDH2 (vs. LAC: *p* = 0.002; vs. EXE: *p* = 0.01), which are related to oxidative metabolism, were expressed at significantly higher levels than in the control group, and no significant difference was observed in the combined treatment EXLA group (Figure 4A,B). Moreover, UCP3 (thermogenesis-related) was expressed at a significantly higher level in the exercise training group than in the control group, regardless of lactate intake (Figure 4C; vs. EXE, *p* = 0.01; vs. EXLA, *p* = 0.005). Additionally, PDHA1 (*p* = 0.01), GLUT4 (*p* = 0.023), and HK2 (*p* < 0.001) were significantly higher in the EXLA group treated with the combination than in the control group (Figure 4E–G). The EXE group showed a significantly higher GLUT4 (*p* = 0.018) and HK2 (*p* = 0.017) expression than that in the control group, and PDHA1 showed a tendency to increase, although the difference was not significant (*p* = 0.06; Figure 4E–G). Although there was no statistically significant difference between the LAC and control groups, a tendency for higher expression of PDHA1 (*p* = 0.063) and GLUT4 (*p* = 0.082) was observed (Figure 4E,F). 

In summary, independent exogenous lactate intake increased the expressions of proteins that activate oxidative metabolism in skeletal muscles, and when combined with exercise, increased the expression of factors related to carbohydrate oxidation.

## 4. Discussion

Investigating the effects of chronic lactate administration on the changes in body composition, energy metabolism, and substrate utilization to confirm the possibility of using lactate as a metabolic regulator is necessary. Comparing exercise-induced metabolic changes with chronic lactate administration and determining whether the combined treatments have an additional effect is also crucial. Therefore, we examined the effects of independent exogenous lactate administration, exercise training, and combined treatments on energy substrate selection, energy metabolism-related protein expression in skeletal muscles, and body composition changes at rest and during exercise. Finally, we also examined exogenous lactate availability.

Independent lactate administration, exercise training, and combined treatment increased energy expenditure by increasing carbohydrate oxidation as an energy substrate (Figure 2). Assuming the “crossover” perspective where energy substrate adaptation occurs depending on exercise intensity [24], a training intensity of 60–70% VO_2_ max improved carbohydrate use rather than fat oxidation. In addition, the intensity set in the metabolic measurement during exercise was approximately 60% VO_2_ max, which implies increased carbohydrate use [25,26], ultimately leading to increased energy expenditure induced by increased carbohydrate oxidation. The EXE group showed a higher expression of CS [27,28] and MDH2 [29] than that in the CON group—key factors in the TCA and GLUT4 cycles, the latter of which is very important in skeletal muscle carbohydrate transport during exercise [30]. In addition, UCP3 expression [31,32], a factor related to skeletal muscle thermogenesis, stimulating glucose transport, and GLUT4 translocation, was significantly higher than that the control group. HK2 [33,34], the rate-limiting enzyme of glycolysis, was significantly higher than that in the control group.

These results indicate that exercise training increases the transport and utilization of carbohydrates through UCP3, GLUT4, and HK2 activation by upregulating energy substrate utilization-related proteins in skeletal muscle, and it may also affect TCA cycle activation, which is an efficient energy production process.

Notably, the LAC group also showed significantly increased energy expenditure during exercise owing to increased carbohydrate utilization. Although few studies have directly confirmed the effect of lactate on energy substrate utilization, increased blood lactate inhibits circulating free fatty acids and lipolysis in adipocytes via GPR81 activation [18,35,36]. Consistent with these results, our previous study reported that acute lactate administration before exercise increases carbohydrate oxidation as an energy substrate during exercise and lowers blood lipid concentrations after exercise [22]. Although further research on the mechanisms related to energy substrate use is required, the inhibition of fat energy utilization by continuous lactate treatment may lead to the greater adaptation of factors related to carbohydrate oxidation as an energy substrate. Additionally, energy expenditure may be increased by further increasing carbohydrate availability as a substrate in the case of exercise training, which demands increased energy. Similar to exercise training, lactate administration significantly increased CS and MDH2 expression in skeletal muscle compared to that in the controls (Figure 4). However, lactate administration alone did not significantly increase UCP3, GLUT4, HK2, and PDHA1 protein levels, which are involved in thermogenesis and carbohydrate transport and utilization. Moreover, GLUT4 and PDHA1 levels tended to increase, although not significantly (*p* = 0.08; Figure 4E,F). Although additional research is required, independent lactate administration may activate TCA cycle-related factors in skeletal muscle energy substrate utilization and induce changes (acting as signaling molecules) in metabolic activation, such as in carbohydrate transport and utilization. The exercise training groups had significantly less body fat and improved body composition compared to the control group, regardless of lactate intake. Exercise training increases resting energy expenditure through changes in energy metabolism adaptation [8,37] and increases fat-free mass [38,39]. Exercise-induced total energy expenditure effectively improves body fat [40]. The trend of reduced body fat after chronic lactate was not significantly different (Figure 3; *p* = 0.08). Similarly, the resting metabolic rate results indicate that lactate intake tended to increase energy expenditure, although the difference was not statistically significant. Considering that there were no differences in fat-free mass among the groups in the body composition results, the increased resting energy expenditure could be a result of changes in tissue metabolic adaptation due to lactate intake. Moreover, the resting energy expenditure in the lactate intake group increased by approximately 8% compared to that of the control group, suggesting that this slight increase in energy expenditure may have accumulated over the experimental period and influenced body fat changes. There were no significant differences in the dietary intake of any group during the experimental period (Supplementary Figure S4).

Finally, the additional effect of the combined lactate administration and exercise training was minor. This suggests that the endogenous lactate flux generated during exercise may be sufficient to provide a signaling molecule to other tissues and organs. Lactate flux is proportional to exercise intensity [41]; this study was set at high intensity. In exercise situations where the lactate flux increases significantly, lactate can be regenerated as an energy source and delivered to other organs to play a sufficient role as a signaling molecule [13,41]. Therefore, in situations in which endogenous lactate is sufficient, the effect of exogenous lactate administration may be masked. The combined treatment significantly increased PDHA1, a factor involved in the rate-limiting step of aerobic carbohydrate metabolism in the skeletal muscle [42]. We also observed that the combination treatment group had the highest amount of carbohydrate oxidation in the RMR (Supplementary Figure S1E). We also measured the expression levels of electron transport chain proteins involved in oxidative phosphorylation, and found that only the combined treatment significantly increased the expression of complexes I, III, and IV (Supplementary Figure S3). The OXPHOS I–III–IV supercomplex is used to pump protons via electron transport from NADH to oxygen [43,44]. Although additional studies are required, the high expression of the I–III–IV supercomplex in the combined treatment group is considered to be a result of processing the large amounts of NADH generated from the conversion of additional exogenous lactate administered to pyruvate after exercise. These results indicate that the combined treatment has the potential to improve overall energy metabolism by activating carbohydrate oxidation pathways and inducing increases in mitochondrial function.

In summary, our study shows that independent lactate administration significantly increased the CS and MDH2 protein levels, which are related to energy substrate utilization in skeletal muscles, and increased energy expenditure via increasing carbohydrate oxidation during exercise. Although there was no statistical difference, lactate intake increased resting energy expenditure by approximately 8%, and it is possible that this slight increase accumulated over the experiment and influenced body fat changes. Collectively, these results suggest that lactate, a metabolic regulator, induces metabolic changes. Although independent lactate administration does not lead to as much improvement in body composition as exercise training, there is a clear possibility that it can increase energy expenditure by improving the substrate utilization ability of skeletal muscle.

In addition, the combined treatment of exogenous lactate and exercise increased the ability to utilize carbohydrates in metabolic measurements at rest and during exercise, which likely occurred due to the high UCP3, GLUT4, PDHA1, and HK2 expression, factors related to carbohydrate utilization in skeletal muscle. Moreover, when examining not only carbohydrate oxidation but also the levels of OXPHOS complexes, only the combined treatment showed high levels of the I–III–IV supercomplex, which could represent a possible way to improve mitochondrial function.

The limitations of this study include the fact that the exercise intensity was too high to determine fat utilization ability, and the intervention period was relatively short. We examined the skeletal muscle expression levels of CPT1B, associated with fatty acid oxidation [45], as well as AMPKα and PGC1α, which are associated with mitochondrial biogenesis [46,47]; however, no significant differences were observed among the groups. The intensity of exercise training in the study was set at 60–70% VO_2_max. At this intensity, the utilization of carbohydrates as a substrate increases during exercise, so the activation of carbohydrate-related factors takes priority; therefore, there may be no change in the levels of CPT1B and PGC1α related to oxidative phosphorylation. Furthermore, the experimental period of four weeks that was set to observe the effect of chronic lactate intake may have been too short to confirm significant improvements in energy metabolism and body composition based on metabolic changes in skeletal muscle.

Therefore, to verify the effect of lactate as a metabolic regulator, it is necessary to confirm its effects on metabolism using long-term treatment. In addition, experiments are needed to evaluate the effects of lactate and exercise treatment on obesity-induced impaired energy metabolism and improved glucose tolerance in diet-induced obese mice, considering that lactate treatment activates the carbohydrate oxidation pathway and improves skeletal muscle mitochondria function when combined with exercise.

## 5. Conclusions

Chronic lactate treatment for 4 weeks induces an increase in total energy expenditure by activating factors related to energy substrate utilization in skeletal muscles and increasing carbohydrate oxidation during exercise. These results indicate the potential use of lactate as a metabolic regulator. Nevertheless, this study’s experimental period is considered to have been too short for independent lactate administration to significantly change body fat and resting substrate utilization. Therefore, future research needs to investigate the role of lactate through long-term studies.

## Data Availability

Data supporting the findings of this study are available from the corresponding author upon request.

## Supplementary Materials

The following supporting information can be downloaded at: https://www.mdpi.com/article/10.3390/metabo14040220/s1.
