# Supplementary material for: Chronic Administration of Exogenous Lactate Increases Energy Expenditure during Exercise through Activation of Skeletal Muscle Energy Utilization Capacity in Mice
Source: Metabolites. 2024 Apr 13;14(4):220. doi: 10.3390/metabo14040220 (PMC11052295; doi:10.3390/metabo14040220)
Supplement: Supplementary file 1 [file metabolites-14-00220-s001.zip › Final Supplementary metarials.pdf]

Chronic Administration of Exogenous Lactate Increases Energy Expenditure during Exercise through Activation of Skeletal Muscle Energy Utilization Capacity in Mice

Inkwon Jang [1,2], Sunghwan Kyun [1,2], Deunsol Hwang [1,2] , Taeho Kim [1,2] , Kiwon Lim [1,2,3] , Hun-Young Park [1,2] ,

Sung-Woo Kim [1,2] and Jisu Kim [1,2,*]

[1] Laboratory of Exercise and Nutrition, Department of Sports Medicine and Science in Graduate School, Konkuk University, Seoul 05029, Republic of Korea; inkwon555@konkuk.ac.kr (I.J.); y10345@konkuk.ac.kr (S.K.); hds49@konkuk.ac.kr (D.H.); qwer92224@konkuk.ac.kr (T.K.); exercise@konkuk.ac.kr (K.L.); parkhy1980@konkuk.ac.kr (H.-Y.P.); kswrha@konkuk.ac.kr (S.-W.K.)

[2] Physical Activity and Performance Institute (PAPI), Konkuk University, Seoul 05029, Republic of Korea

[3] Department of Physical Education, Konkuk University, Seoul 05029, Republic of Korea

* Correspondence: kimpro@konkuk.ac.kr; Tel.: +82-10-2049-6034

Primary antibody				Secondary antibody		
Target	Molecular Weight (kDa)	Concentration	Cat. No	Target	Concentration	Cat. No
CS	52	1:2,000	ab96600 (Abcam)	anti-rabbit	1:10,000	ab205718 (Abcam)
MDH2	36	1:2,000	ab96193 (Abcam)		1:20,000	
UCP3	34	1:1,000	ab251173 (Abcam)		1:50,000	
GLUT4	55	1:3,000	ab654 (Abcam)		1:30,000	
CPT1B	87	1:2,000	ab134988 (Abcam)		1:10,000	
PGC1a	113	1:3,000	ab188102 (Abcam)		1:10,000	
GAPDH	37	1:5,000	ab9485 (Abcam)		1:20,000	
PDHA1	46	1:1,000	ab110330 (Abcam)	anti-mouse	1:10,000	ab205719 (Abcam)
HK2	102	1:1,000	sc-130358 (Santa Cruz)		1:10,000	
OxPhos complex	55, 48, 40, 30, 20	1:2,000	45-8099 (Thermo Fisher)		1:30,000	

Table S1. Information of used antibodies for immunoblotting in the experiment.

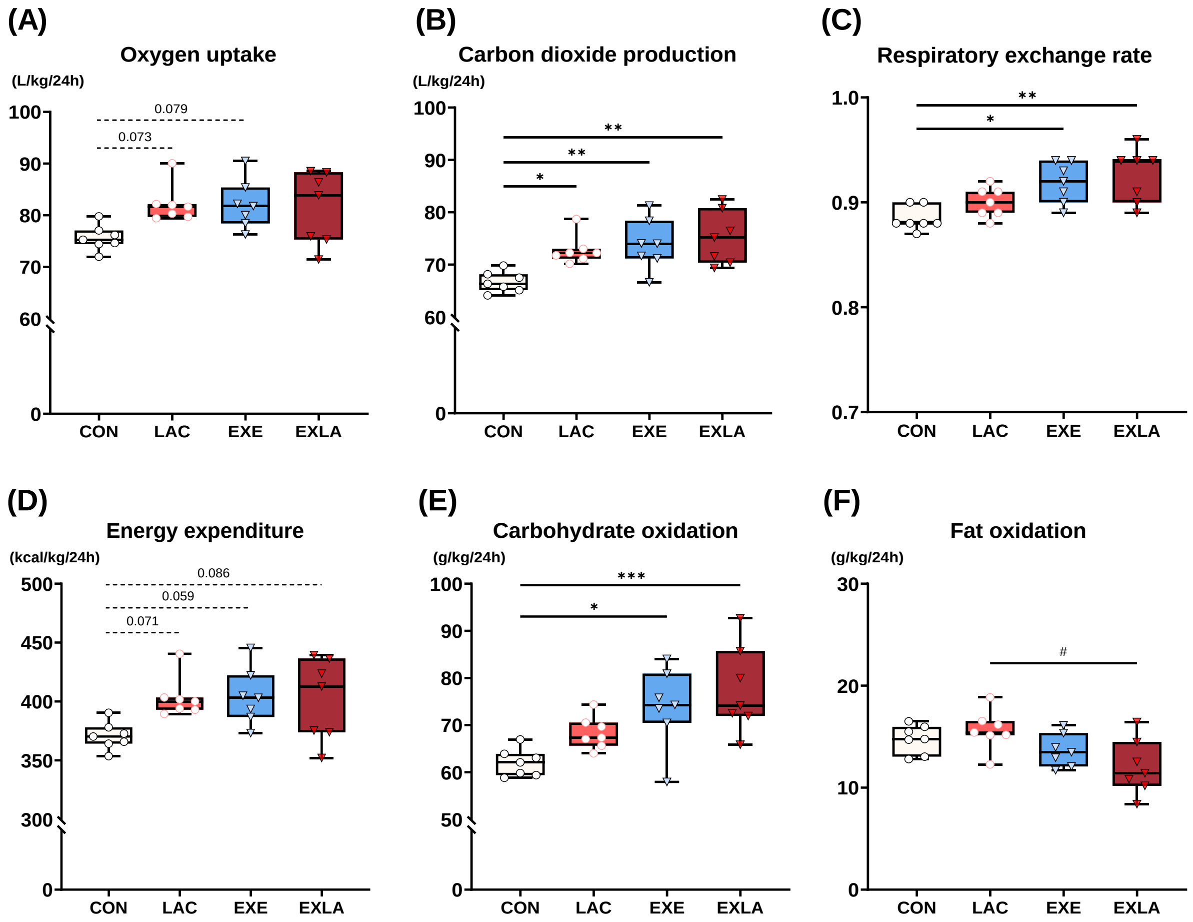

Figure S1. Resting metabolism was measured for 24 h in each independent chamber. The data was a composite of 24 h, and the integrated values were (A) VO_2, (B) VCO_2, (C) RER, (D) EE, (E) CO, and (F) FO, and RER represents the 24-h average value. A one-way ANOVA was performed, and Tukey HSD was used as a post hoc test (n = 7 per group). CON, sedentary and saline administration; LAC, sedentary and lactate administration; EXE, exercise training, and saline administration); EXLA, exercise training and lactate administration; VO_2, oxygen uptake; VCO_2, carbon dioxide production; RER, respiratory exchange rate; EE, energy expenditure; CO, carbohydrate oxidation; FO, fat oxidation. *p<0.05, **p<0.01, ***p<0.001 vs. CON; #p<0.01 vs. LAC. Data are presented as mean ± standard deviation

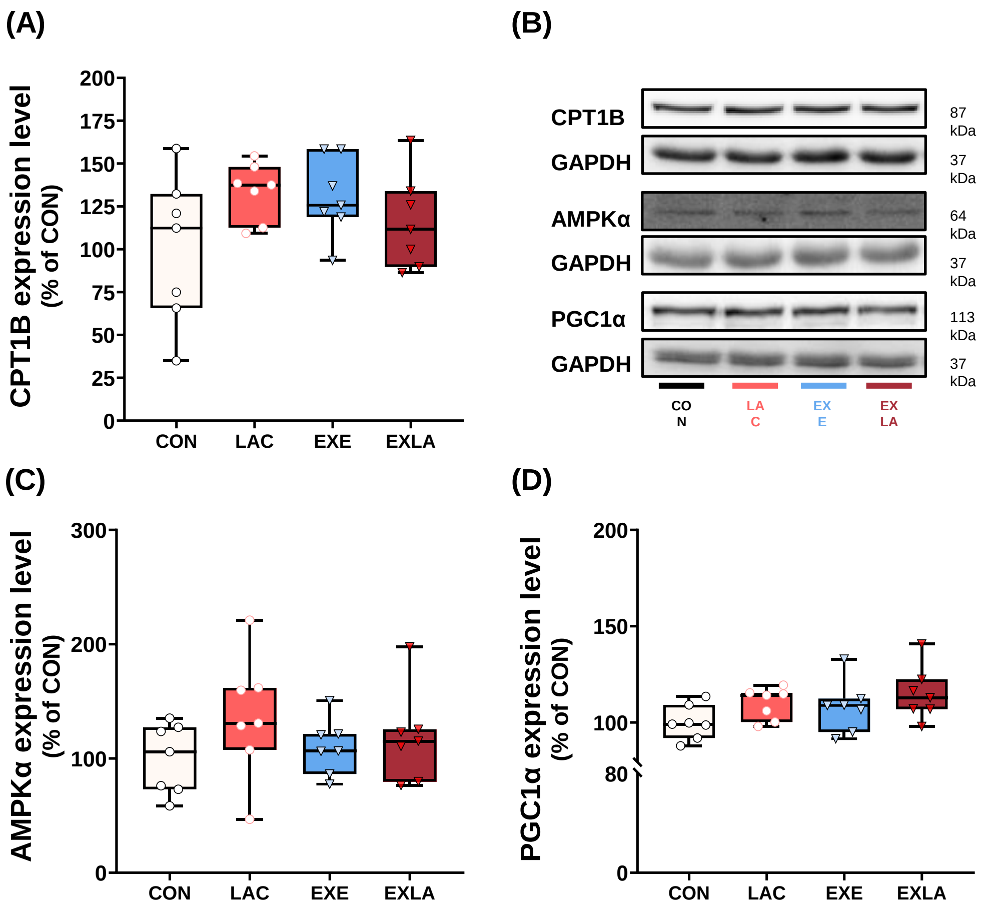

Figure S2. Protein expression levels of the gastrocnemius muscle (A) CPT1B, (C) AMPKα, and (D) PGC1α. (B) represent western blot images. A one-way ANOVA was performed (n = 7 per group). AMPKα, adenosine monophosphate-activated protein kinase alpha; PGC1α, peroxisome proliferator-activated receptor-gamma coactivator 1 alpha. Data are presented as mean ± standard deviation.

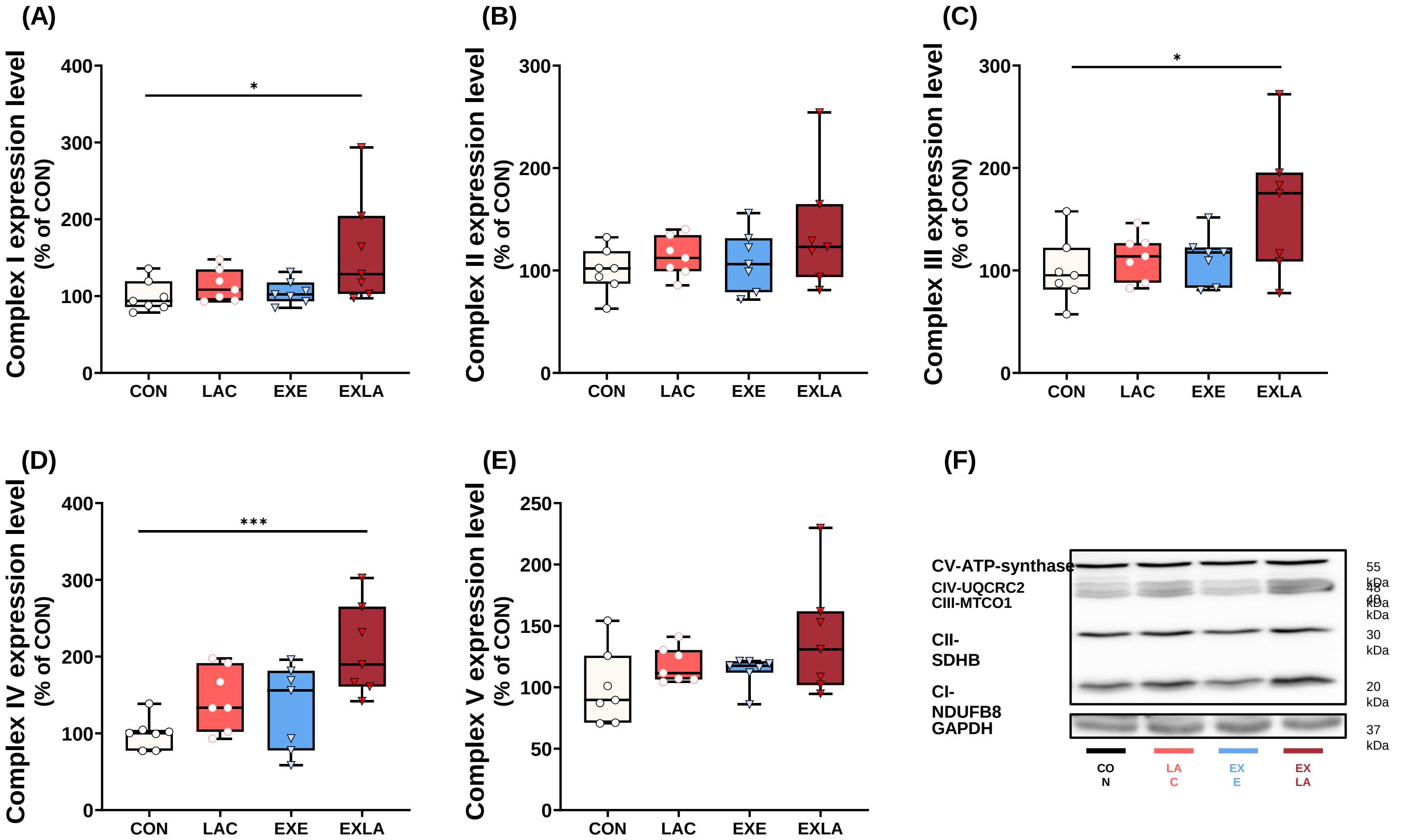

Figure S3. Administration of exogenous lactate activates oxidative metabolism in skeletal muscle. Protein expression levels of the gastrocnemius muscle (A) CI, (B) CII, (C) CIII, (D) CIV, and (E) CV. (F) represent western blot images. A one-way ANOVA was performed, and Tukey HSD was used as a post hoc test (n = 7 per group). CON, sedentary and saline administration; LAC, sedentary and lactate administration; EXE, exercise training, and saline administration); EXLA, exercise training and lactate administration. CI, NADH-ubiquinone oxidoreductase; CII, succinate-coenzyme Q reductase; CIII, cytochrome c–oxidoreductase; CIV, cytochrome c oxidase; CV, ATP-synthase; GAPDH, glyceraldehyde-3-phosphate dehydrogenase. *p<0.05, ***p<0.001 vs. CON. Data are presented as mean ± standard deviation.

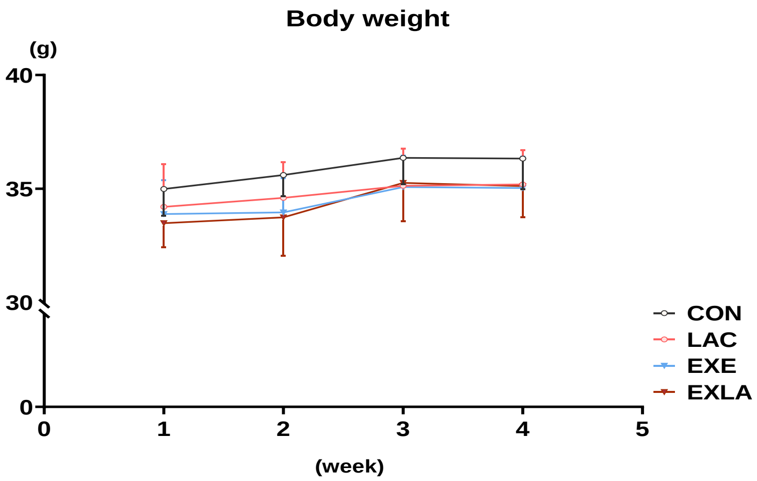

Figure S4. Tracking body weight changes during the experiment. The graph represents the average weight among groups by week. CON, sedentary and saline administration; LAC, sedentary and lactate administration; EXE, exercise training, and saline administration); EXLA, exercise training and lactate administration. Data are presented as mean ± standard deviation.

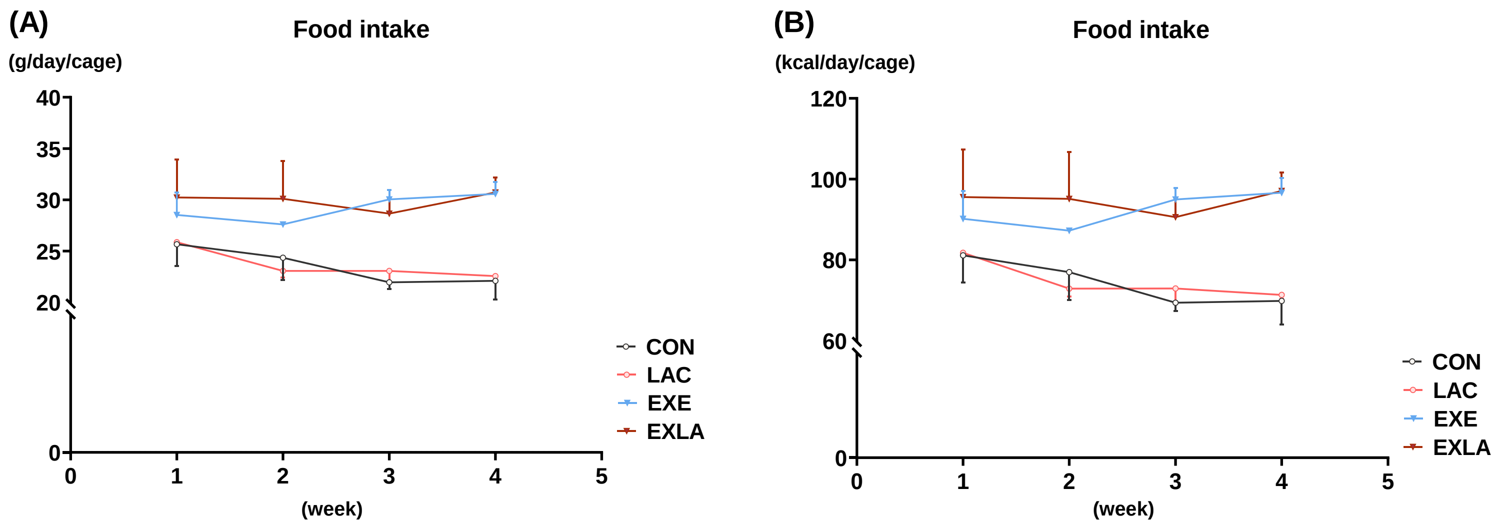

Figure S5. Tracking food intake during the experiment. (A) Average daily food intake by week, (B) Average daily kilocalorie intake by week. The Kruskal-Wallis test was performed (*n* = 2 per group). CON, sedentary and saline administration; LAC, sedentary and lactate administration; EXE, exercise training, and saline administration); EXLA, exercise training and lactate administration. Data are presented as mean ± standard deviation.

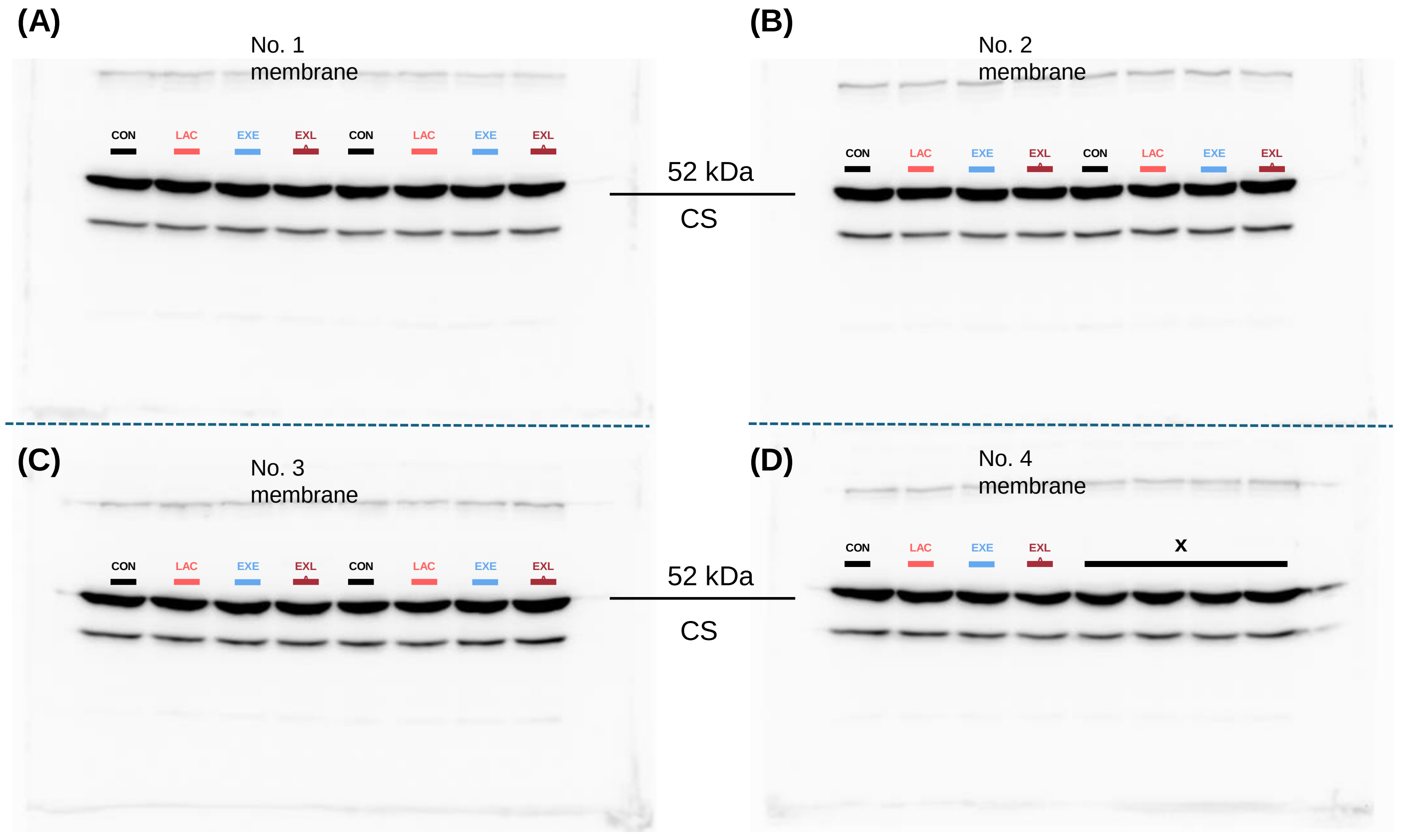

Figure S6. The original blots of skeletal muscle protein expression of CS presented in Fig. 4. The result of (A) CS expression level in membrane No. 1, (B) CS expression level in membrane No. 2, (C) CS expression level in membrane No. 3, and (D) CS expression level in membrane No. 4. CON, sedentary and saline administration; LAC, sedentary and lactate administration; EXE, exercise training, and saline administration); EXLA, exercise training and lactate administration. CS, citrate synthase.

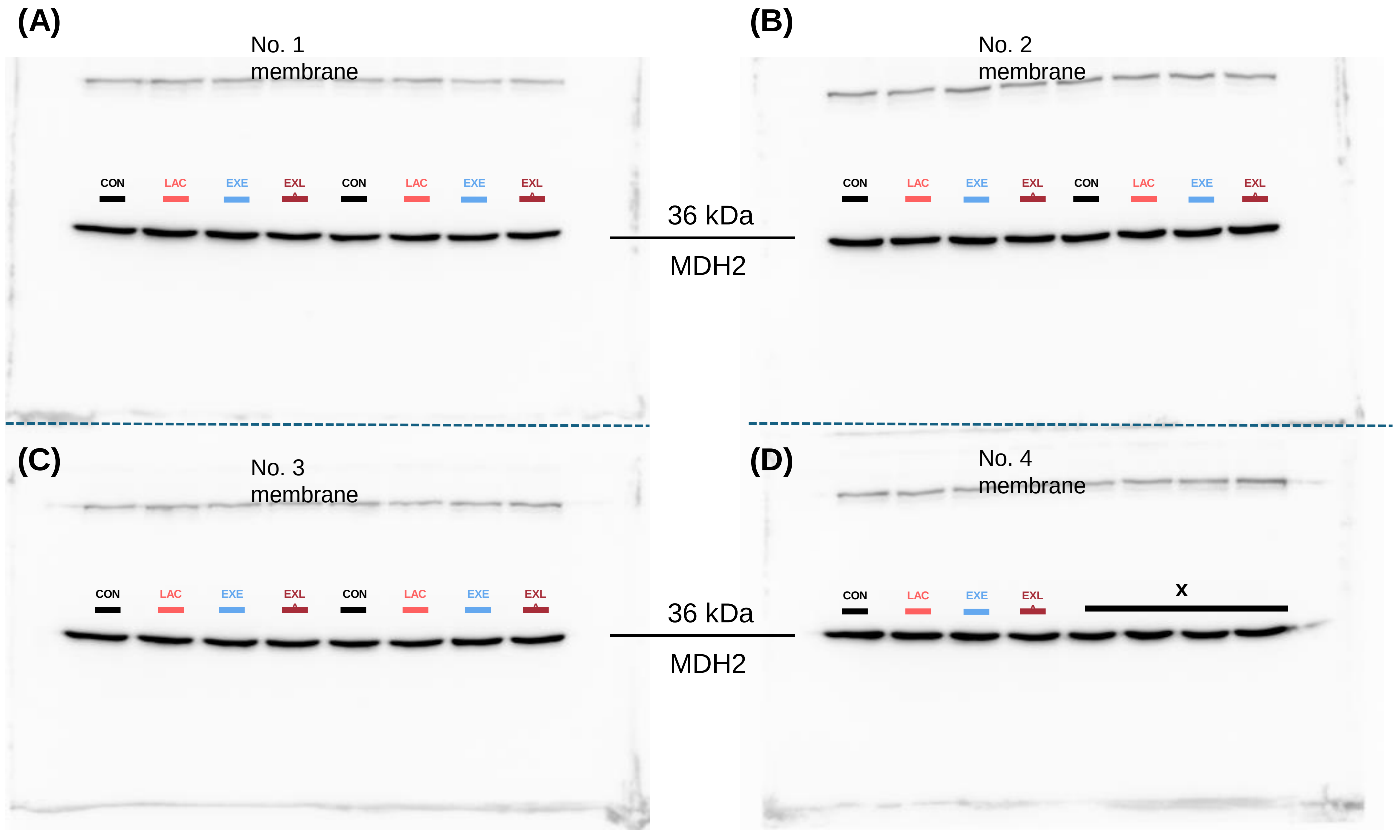

Figure S7. The original blots of skeletal muscle protein expression of MDH presented in Fig. 4. The result of (A) MDH2 expression level in membrane No. 1, (B) MDH2 expression level in membrane No. 2, (C) MDH2 expression level in membrane No. 3, and (D) MDH2 expression level in membrane No. 4. CON, sedentary and saline administration; LAC, sedentary and lactate administration; EXE, exercise training, and saline administration); EXLA, exercise training and lactate administration. MDH2, malate dehydrogenase 2.

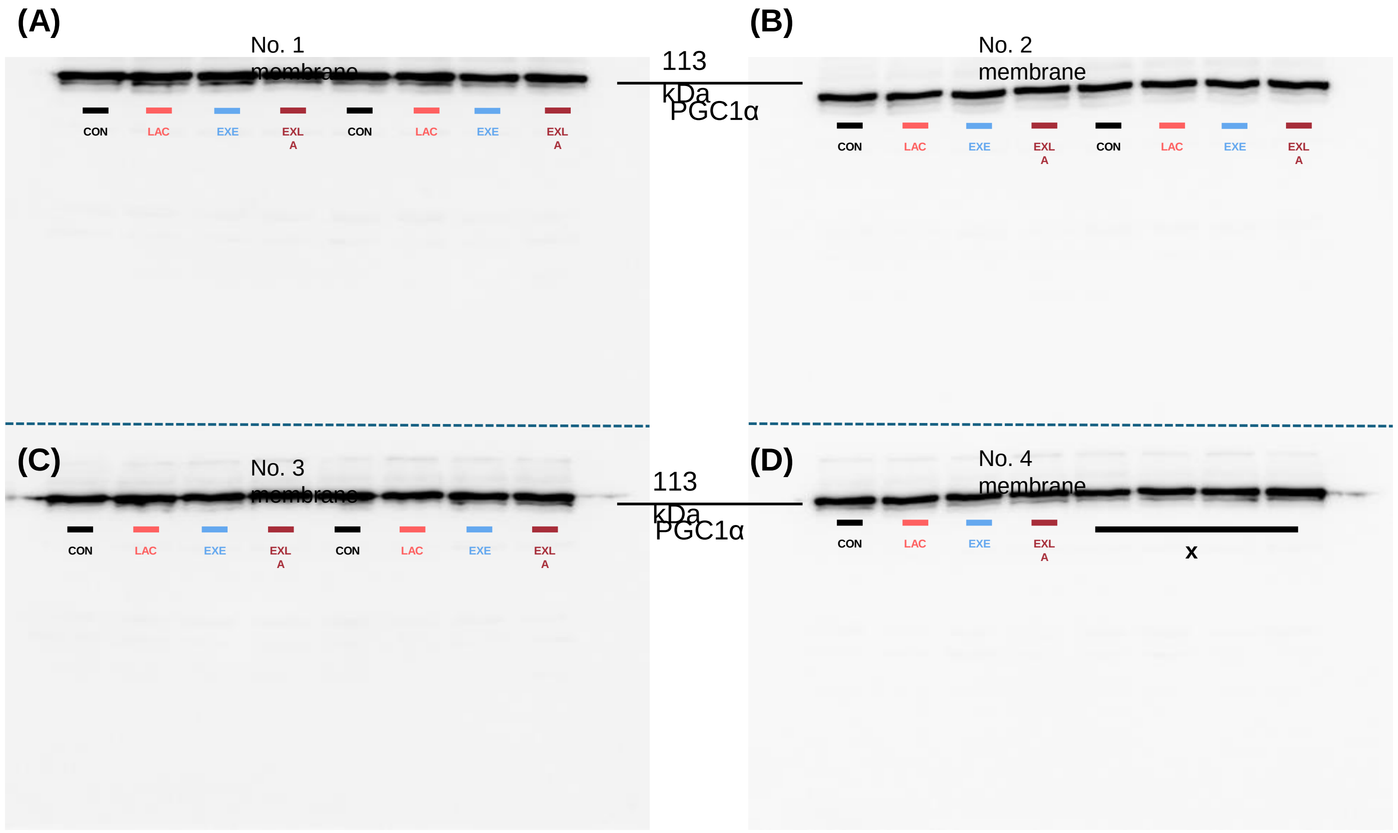

Figure S8. The original blots of skeletal muscle protein expression of PGC1*α* presented in Supplementary Fig. 2. The result of (A) PGC1*α* expression level in membrane No. 1, (B) PGC1*α* expression level in membrane No. 2, (C) PGC1*α* expression level in membrane No. 3, and (D) PGC1*α* expression level in membrane No. 4. CON, sedentary and saline administration; LAC, sedentary and lactate administration; EXE, exercise training, and saline administration); EXLA, exercise training and lactate administration. peroxisome proliferator-activated receptor-gamma coactivator 1 alpha.

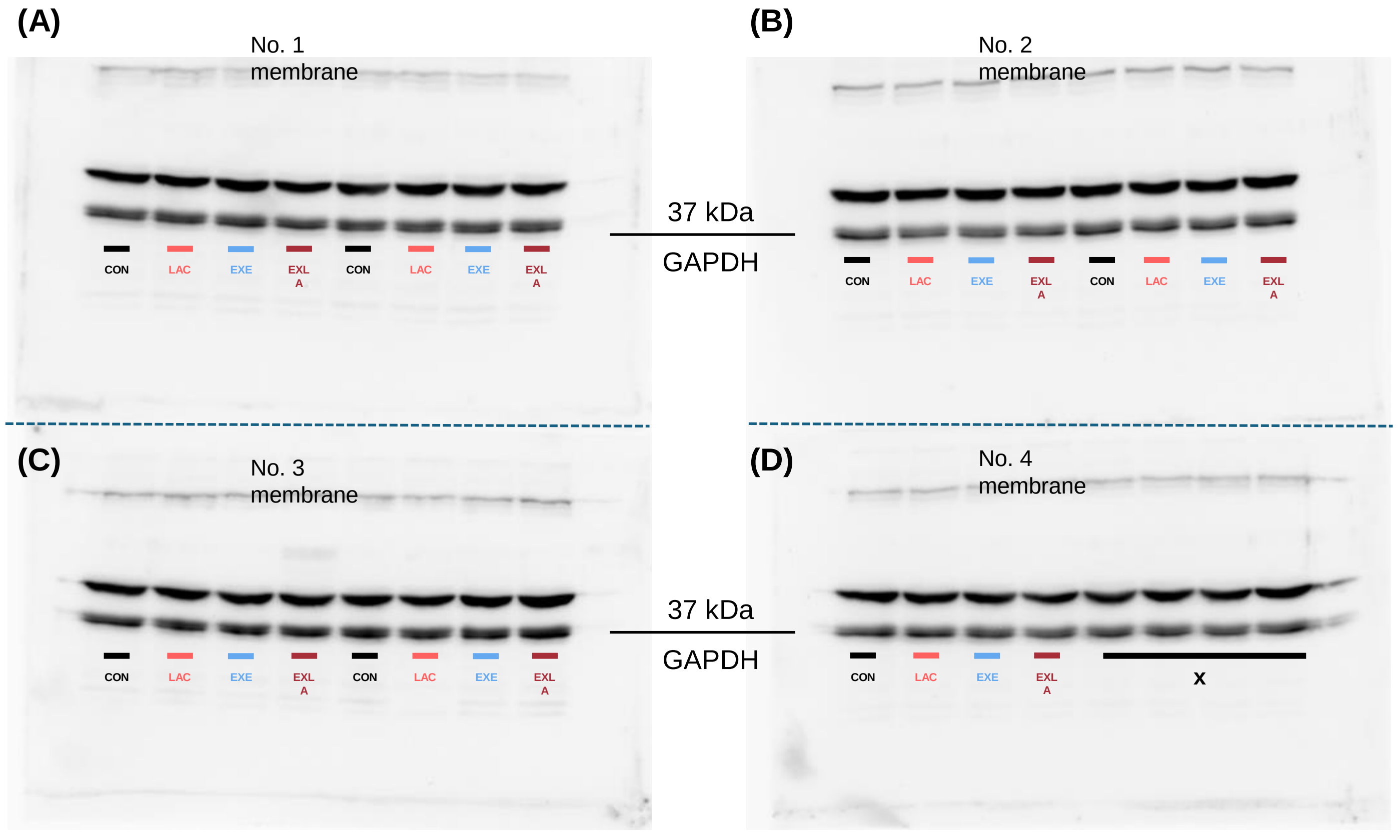

Figure S9. The original blots of skeletal muscle protein expression of GAPDH presented in Fig. 4. The result of (A) GAPDH expression level in membrane No. 1, (B) GAPDH expression level in membrane No. 2, (C) GAPDH expression level in membrane No. 3, and (D) GAPDH expression level in membrane No. 4. CON, sedentary and saline administration; LAC, sedentary and lactate administration; EXE, exercise training, and saline administration); EXLA, exercise training and lactate administration. GAPDH, glyceraldehyde-3-phosphate dehydrogenase.

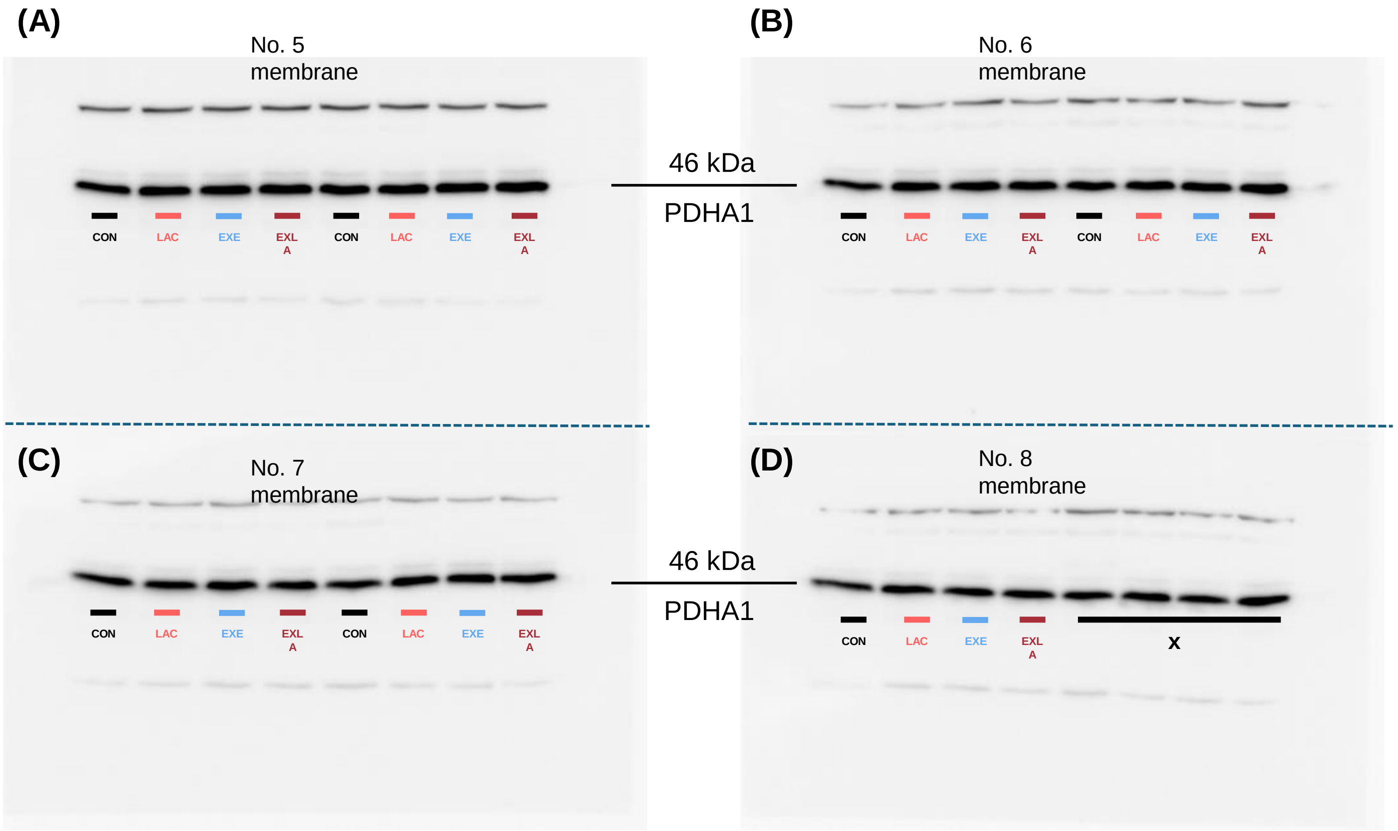

Figure S10. The original blots of skeletal muscle protein expression of PDHA1 presented in Fig. 4. The result of (A) PDHA1 expression level in membrane No. 5, (B) PDHA1 expression level in membrane No. 6, (C) PDHA1 expression level in membrane No. 7, and (D) PDHA1 expression level in membrane No. 8. CON, sedentary and saline administration; LAC, sedentary and lactate administration; EXE, exercise training, and saline administration); EXLA, exercise training and lactate administration. PDHA1, pyruvate dehydrogenase E1 subunit alpha 1.

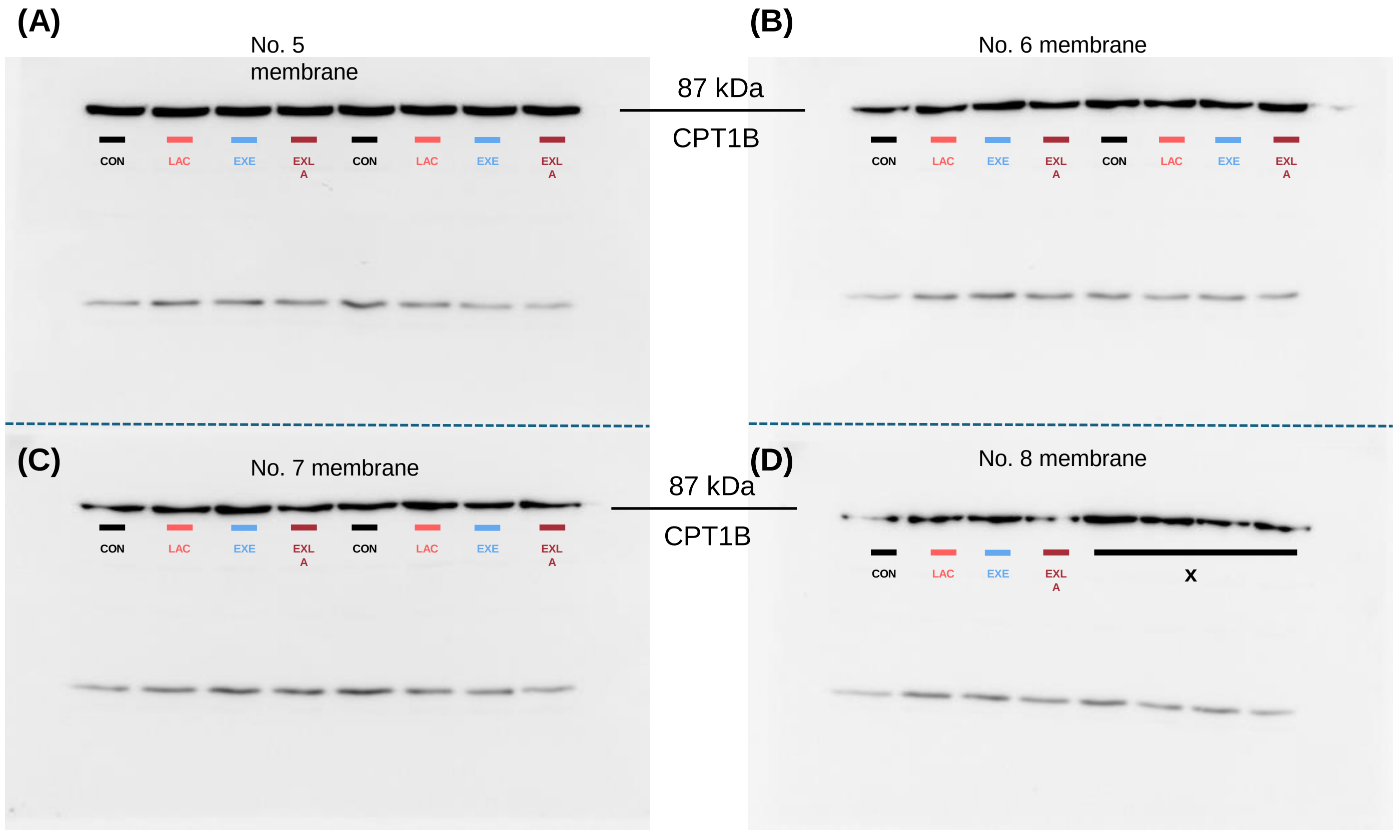

Figure S11. The original blots of skeletal muscle protein expression of CPT1B presented in Supplementary Fig. 2A. The result of (A) CPT1B expression level in membrane No. 5, (B) CPT1B expression level in membrane No. 6, (C) CPT1B expression level in membrane No. 7, and (D) CPT1B expression level in membrane No. 8. CON, sedentary and saline administration; LAC, sedentary and lactate administration; EXE, exercise training, and saline administration); EXLA, exercise training and lactate administration. CPT1B, carnitine palmitoyltransferase 1B.

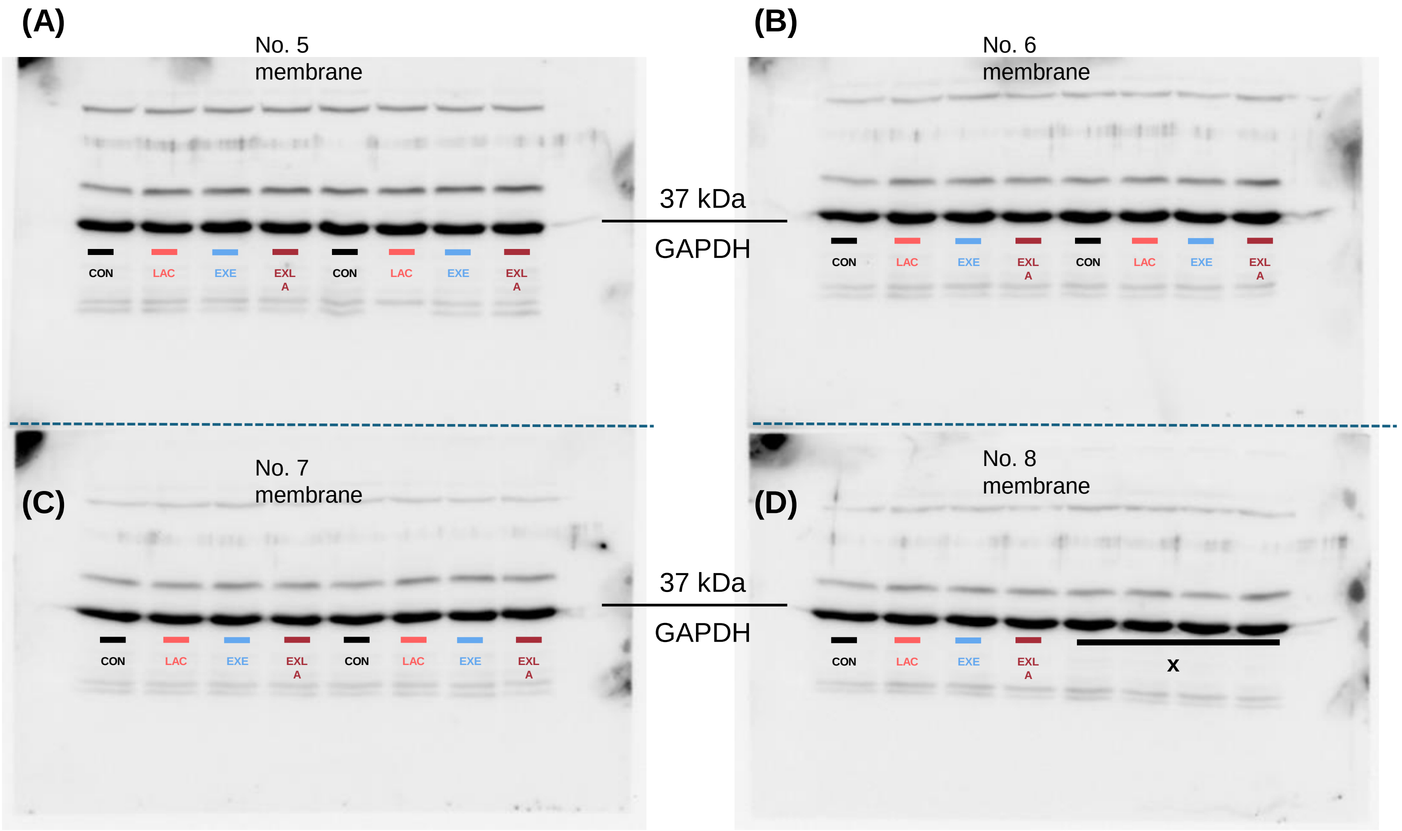

Figure S12. The original blots of skeletal muscle protein expression of GAPDH presented in Fig. 4F. The result of (A) GAPDH expression level in membrane No. 5, (B) GAPDH expression level in membrane No. 6, (C) GAPDH expression level in membrane No. 7, and (D) GAPDH expression level in membrane No. 8. CON, sedentary and saline administration; LAC, sedentary and lactate administration; EXE, exercise training, and saline administration); EXLA, exercise training and lactate administration. GAPDH, glyceraldehyde-3-phosphate dehydrogenase.

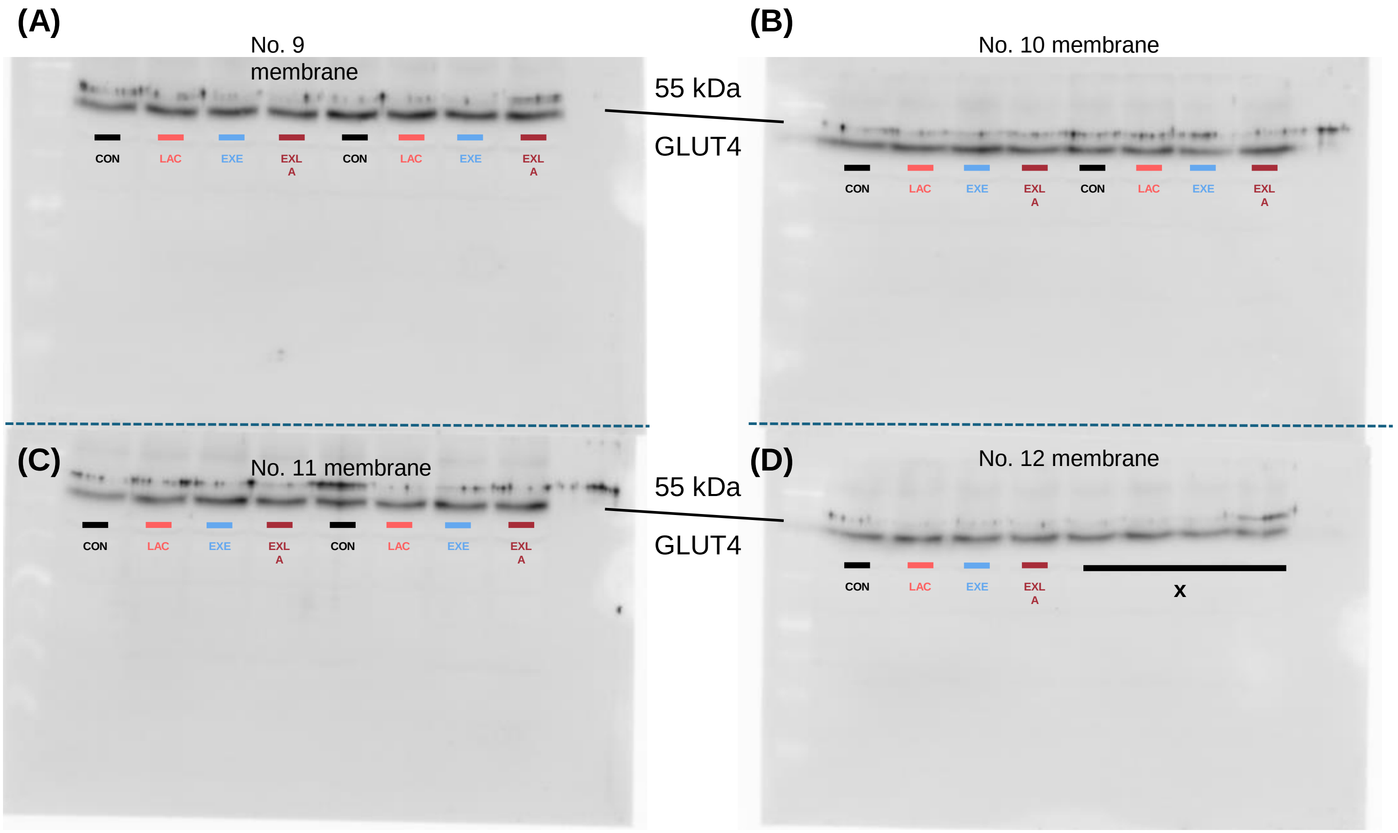

Figure S13. The original blots of skeletal muscle protein expression of GLUT4 presented in Fig. 4. The result of (A) GLUT4 expression level in membrane No. 9, (B) GLUT4 expression level in membrane No. 10, (C) GLUT4 expression level in membrane No. 11, and (D) GLUT4 expression level in membrane No. 12. CON, sedentary and saline administration; LAC, sedentary and lactate administration; EXE, exercise training, and saline administration); EXLA, exercise training and lactate administration. GLUT4, glucose transporter 4.

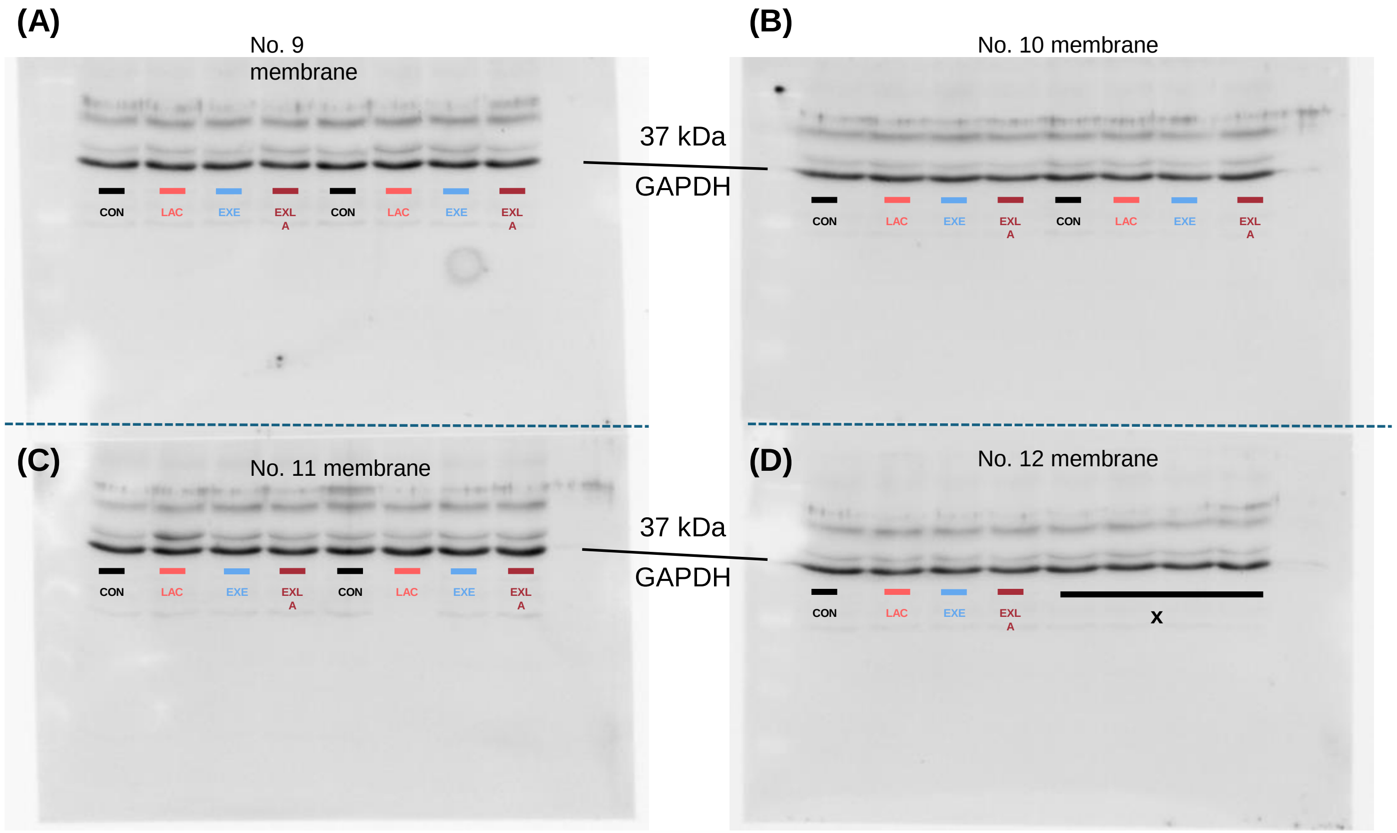

Figure S14. The original blots of skeletal muscle protein expression of GAPDH presented in Fig. 4. The result of (A) GAPDH expression level in membrane No. 9, (B) GAPDH expression level in membrane No. 10, (C) GAPDH expression level in membrane No. 11, and (D) GAPDH expression level in membrane No. 12. CON, sedentary and saline administration; LAC, sedentary and lactate administration; EXE, exercise training, and saline administration); EXLA, exercise training and lactate administration. GAPDH, glyceraldehyde-3-phosphate dehydrogenase.

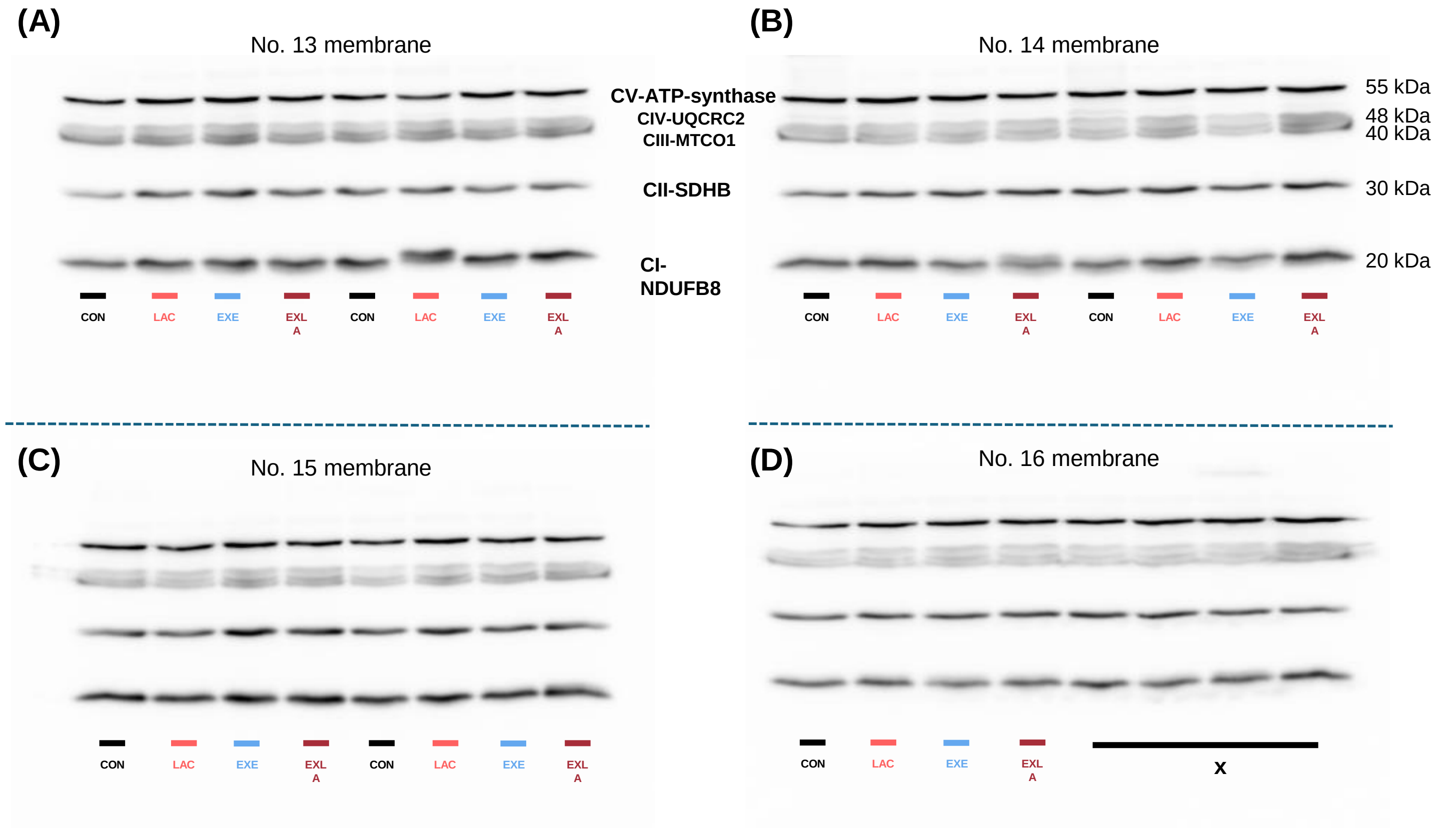

Figure S15. The original blots of skeletal muscle protein expression of OxPhos complexes presented in Supplementary Fig. 3. The result of (A) OxPhos complexes expression level in membrane No. 13, (B)) OxPhos complexes expression level in membrane No. 14, (C)) OxPhos complexes expression level in membrane No. 15, and (D)) OxPhos complexes expression level in membrane No. 16. CON, sedentary and saline administration; LAC, sedentary and lactate administration; EXE, exercise training, and saline administration); EXLA, exercise training and lactate administration. CI, NADH-ubiquinone oxidoreductase; CII, succinate-coenzyme Q reductase; CIII, cytochrome c–oxidoreductase; CIV, cytochrome c oxidase; CV, ATP-synthase.

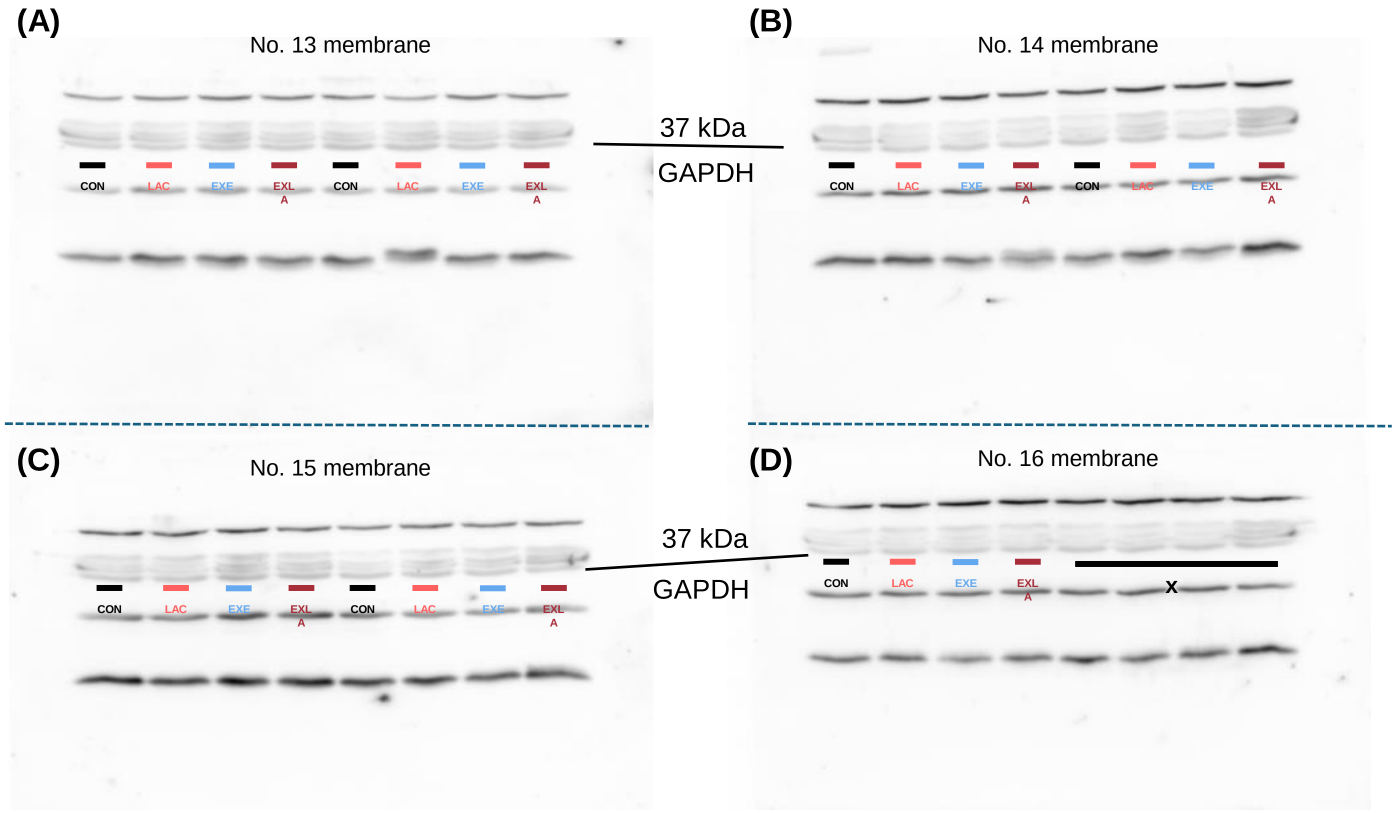

Figure S16. The original blots of skeletal muscle protein expression of GAPDH presented in Supplementary Fig. 3. The result of (A) GAPDH expression level in membrane No. 13, (B) GAPDH expression level in membrane No. 14, (C) GAPDH expression level in membrane No. 15, and (D) GAPDH expression level in membrane No. 16. CON, sedentary and saline administration; LAC, sedentary and lactate administration; EXE, exercise training, and saline administration); EXLA, exercise training and lactate administration. GAPDH, glyceraldehyde-3-phosphate dehydrogenase.

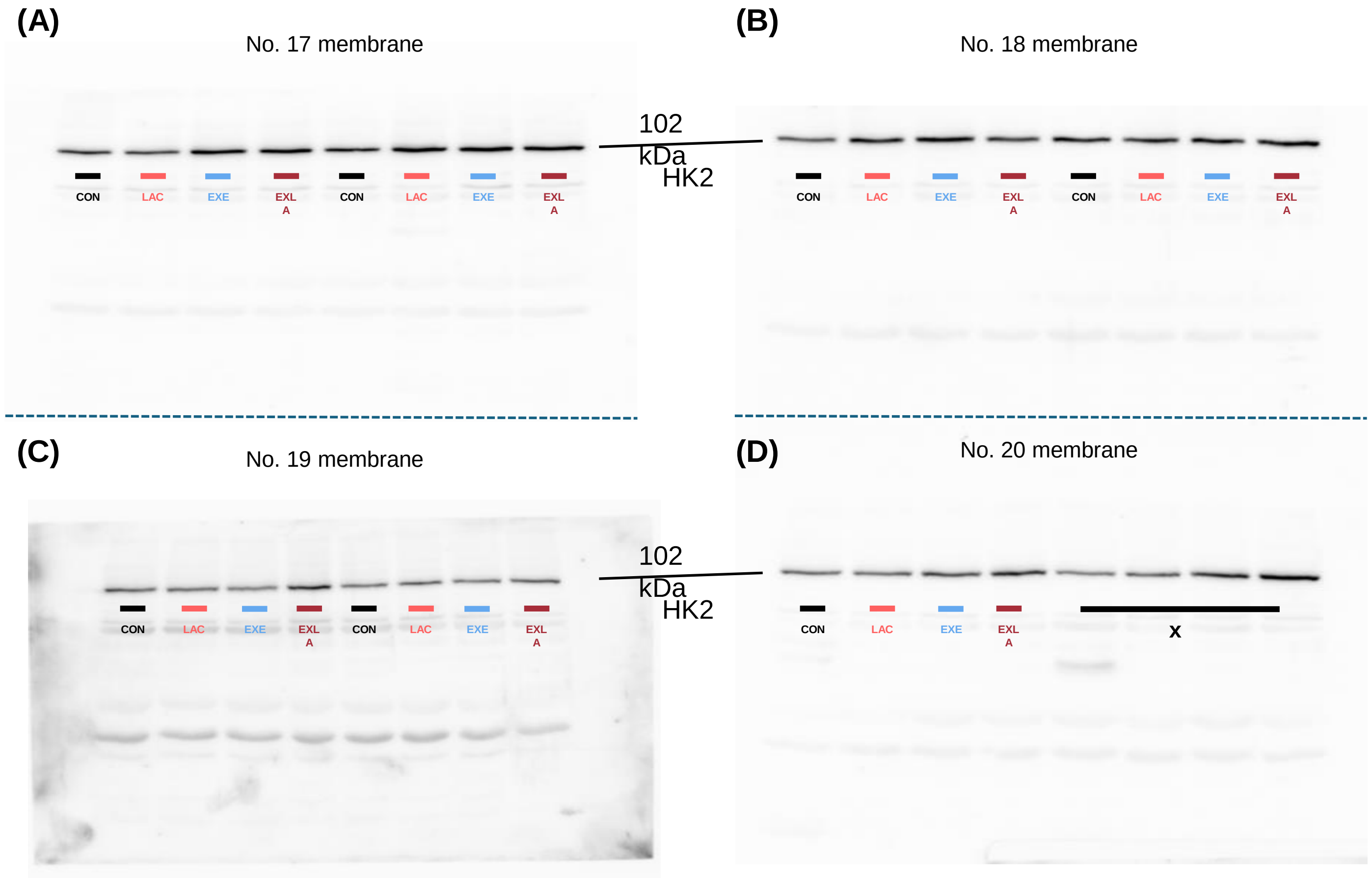

Figure S17. The original blots of skeletal muscle protein expression of HK2 presented in Fig. 4. The result of (A) HK2 expression level in membrane No. 17, (B) HK2 expression level in membrane No. 18, (C) HK2 expression level in membrane No. 19, and (D) HK2 expression level in membrane No. 20. CON, sedentary and saline administration; LAC, sedentary and lactate administration; EXE, exercise training, and saline administration); EXLA, exercise training and lactate administration. HK2, hexokinase 2.

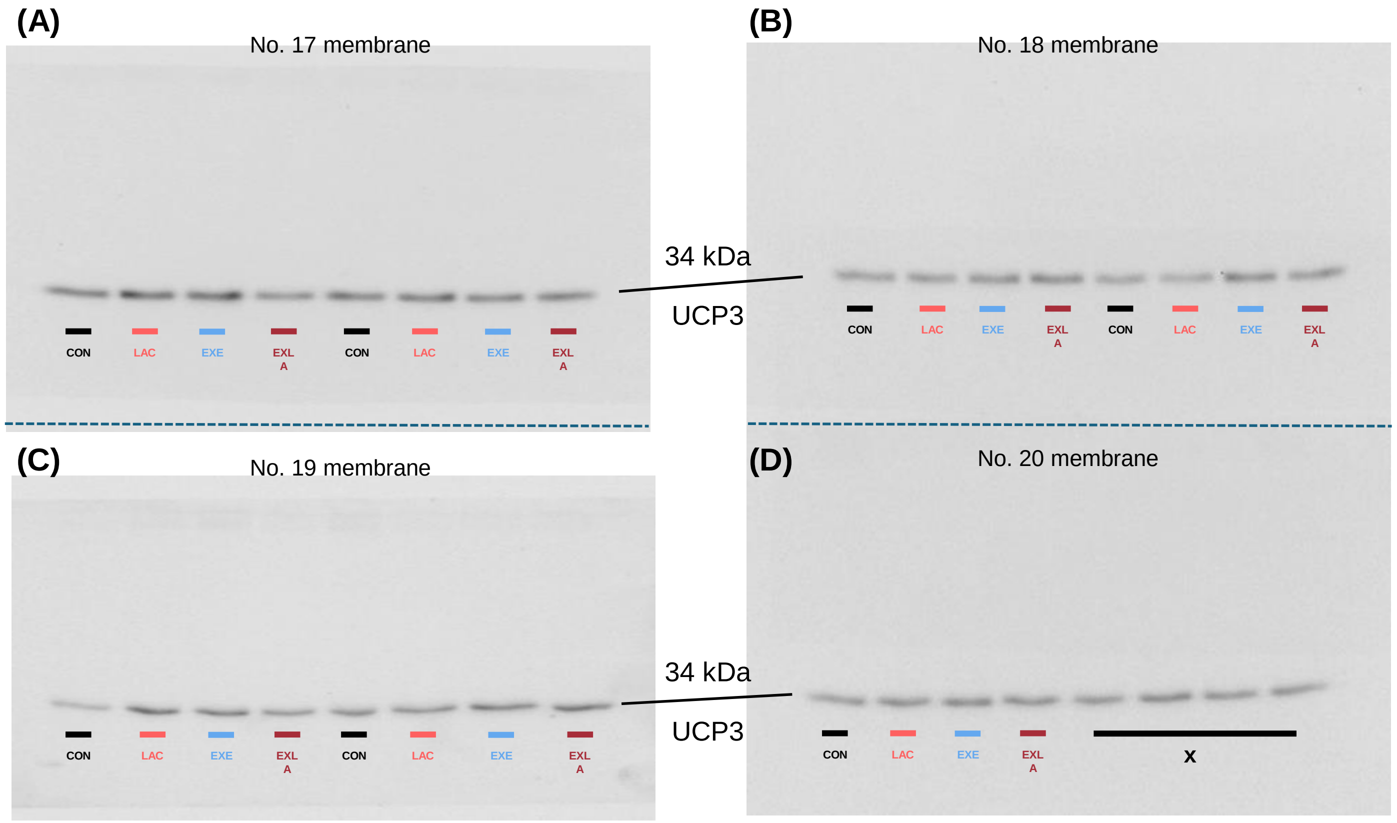

Figure S18. The original blots of skeletal muscle protein expression of UCP3 presented in Fig. 4. The result of (A) UCP3 expression level in membrane No. 17, (B) UCP3 expression level in membrane No. 18, (C) UCP3 expression level in membrane No. 19, and (D) UCP3 expression level in membrane No. 20. CON, sedentary and saline administration; LAC, sedentary and lactate administration; EXE, exercise training, and saline administration); EXLA, exercise training and lactate administration. UCP3, uncoupling protein 3.

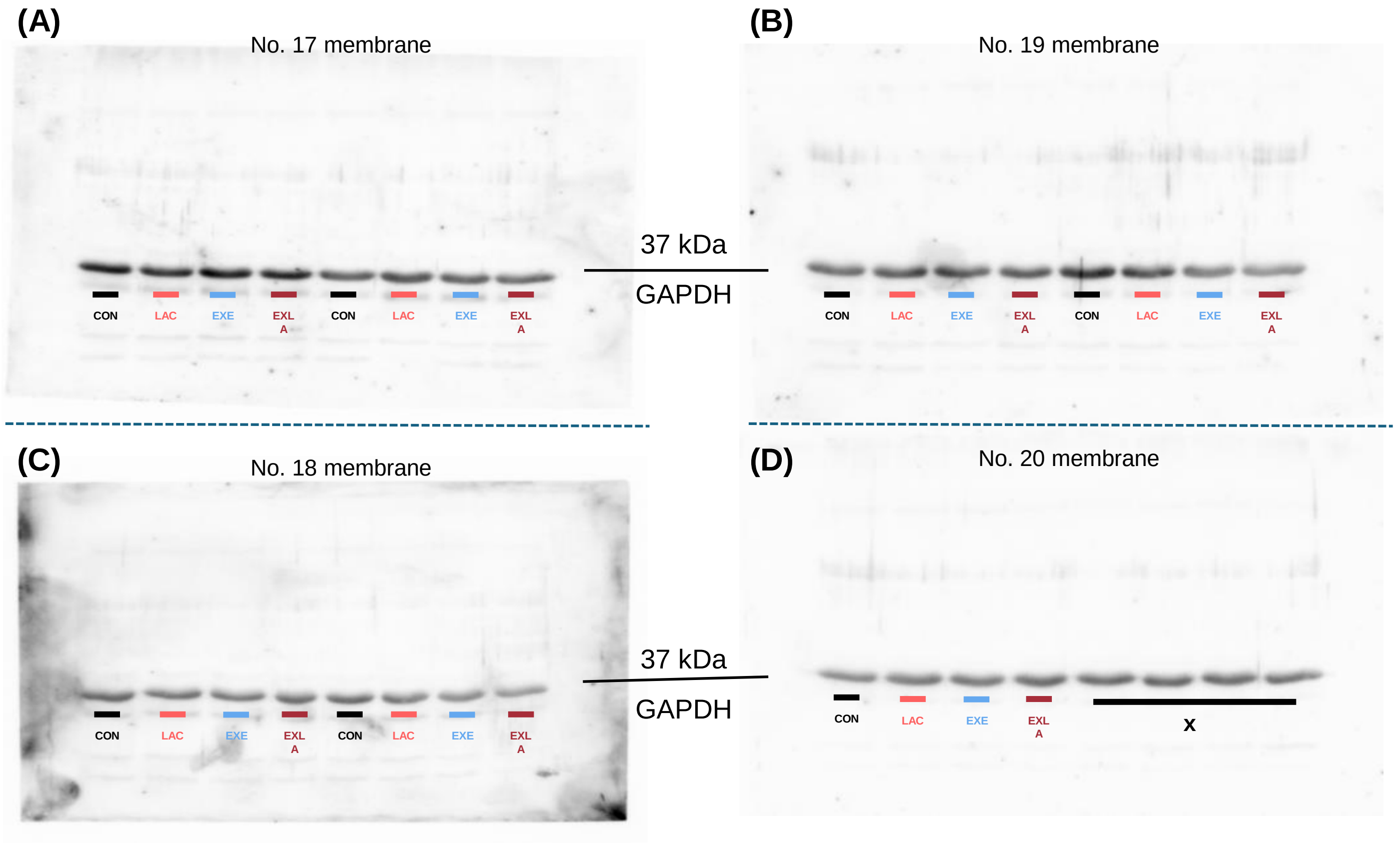

Figure S19. The original blots of skeletal muscle protein expression of GAPDH presented in Fig. 4. The result of (A) GAPDH expression level in membrane No. 17, (B) GAPDH expression level in membrane No. 18 (C) GAPDH expression level in membrane No. 19, and (D) GAPDH expression level in membrane No. 20. CON, sedentary and saline administration; LAC, sedentary and lactate administration; EXE, exercise training, and saline administration); EXLA, exercise training and lactate administration. GAPDH, glyceraldehyde-3-phosphate dehydrogenase.

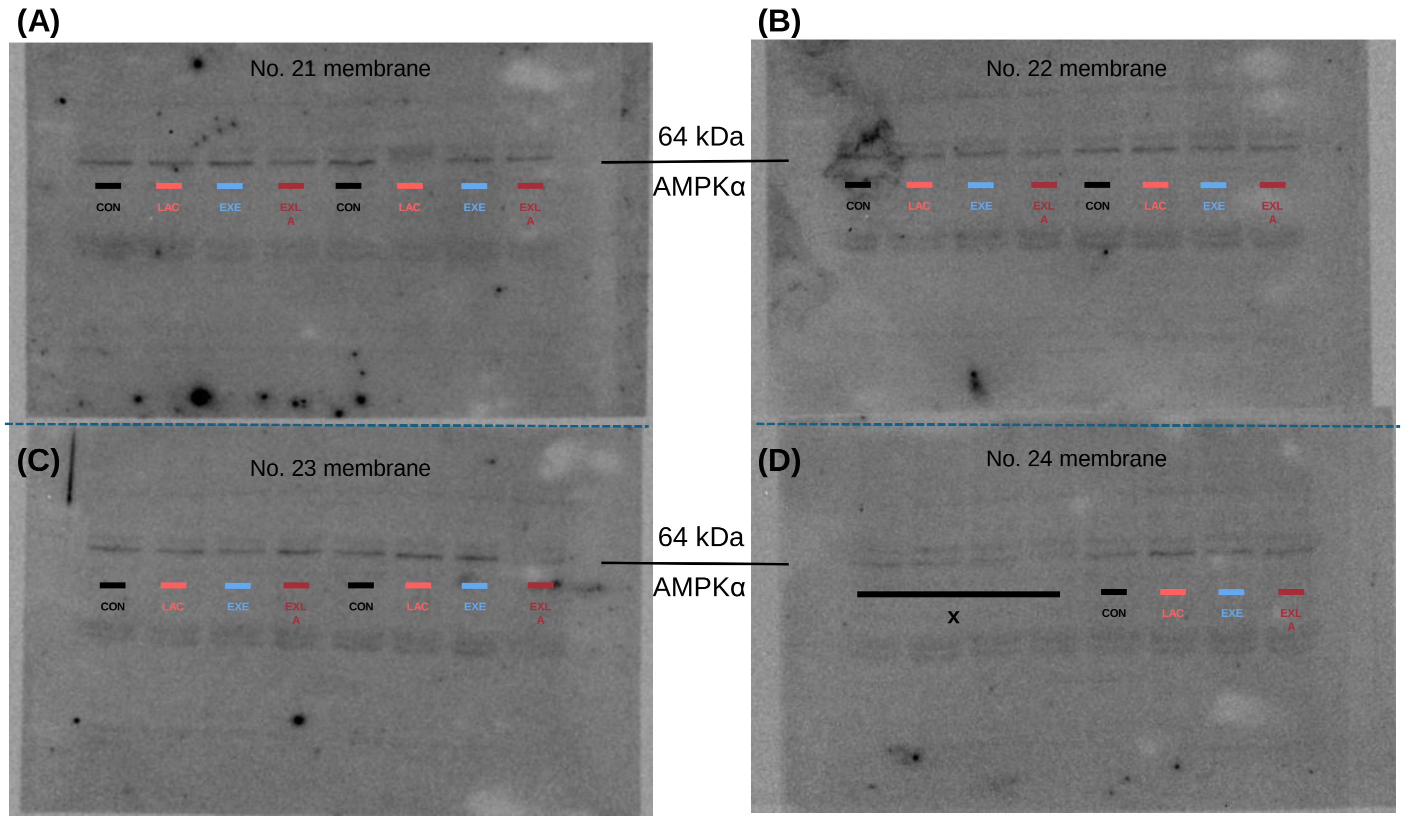

Figure S20. The original blots of skeletal muscle protein expression of AMPKα presented in Supplementary Figure. 2. The result of (A) AMPKα expression level in membrane No. 21, (B) AMPKα expression level in membrane No. 22, (C) AMPKα expression level in membrane No. 23, and (D) AMPKα expression level in membrane No. 24. CON, sedentary and saline administration; LAC, sedentary and lactate administration; EXE, exercise training, and saline administration); EXLA, exercise training and lactate administration. AMPKα, adenosine monophosphate-activated protein kinase alpha

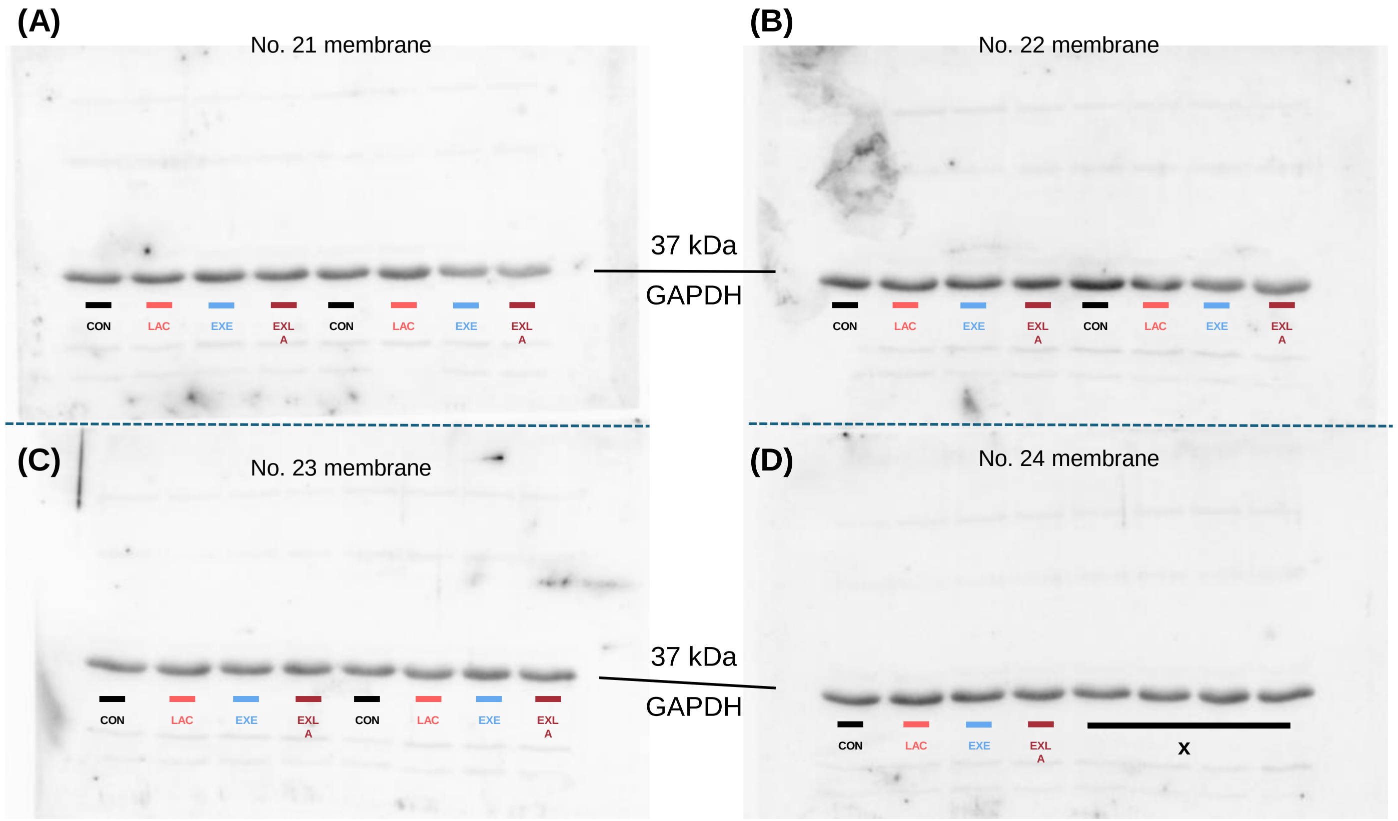

Figure S21. The original blots of skeletal muscle protein expression of GAPDH presented in Supplementary Figure. 2. The result of (A) GAPDH expression level in membrane No. 21, (B) GAPDH expression level in membrane No. 22, (C) GAPDH expression level in membrane No. 23, and (D) GAPDH expression level in membrane No. 24. CON, sedentary and saline administration; LAC, sedentary and lactate administration; EXE, exercise training, and saline administration); EXLA, exercise training and lactate administration. GAPDH, glyceraldehyde-3-phosphate dehydrogenase.
