# Supplementary material for: Chronic Administration of Exogenous Lactate Increases Energy Expenditure during Exercise through Activation of Skeletal Muscle Energy Utilization Capacity in Mice
Source: Metabolites. 2024 Apr 13;14(4):220. doi: 10.3390/metabo14040220 (PMC11052295; doi:10.3390/metabo14040220)
Supplement: Supplementary file 1 [file metabolites-14-00220-s001.zip › Final Supplementary metarials.pptx]

## Slide 1
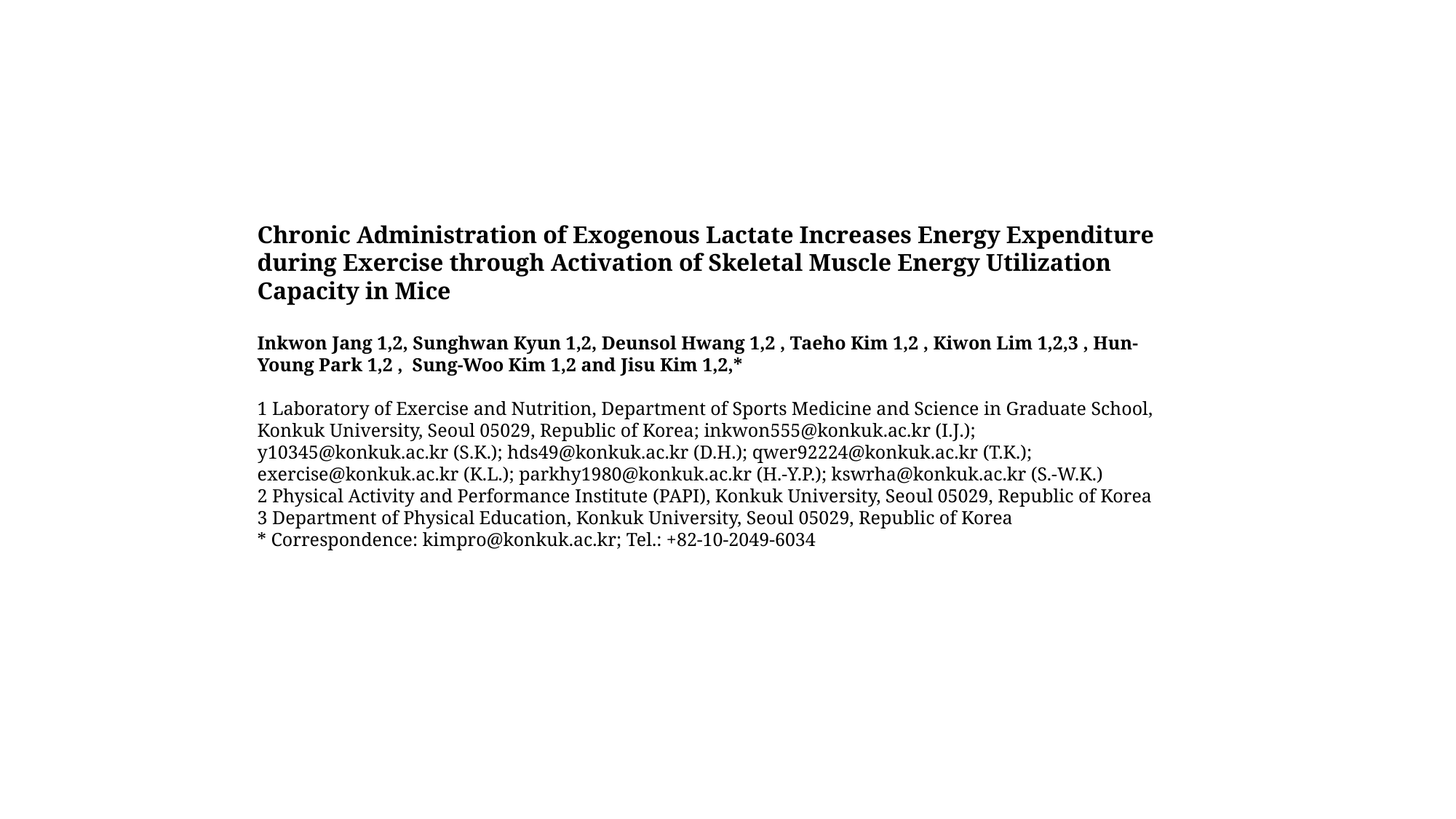

Chronic Administration of Exogenous Lactate Increases Energy Expenditure during Exercise through Activation of Skeletal Muscle Energy Utilization Capacity in Mice
Inkwon Jang 1,2, Sunghwan Kyun 1,2, Deunsol Hwang 1,2 , Taeho Kim 1,2 , Kiwon Lim 1,2,3 , Hun-Young Park 1,2 , Sung-Woo Kim 1,2 and Jisu Kim 1,2,*
1 Laboratory of Exercise and Nutrition, Department of Sports Medicine and Science in Graduate School,
Konkuk University, Seoul 05029, Republic of Korea; inkwon555@konkuk.ac.kr (I.J.);
y10345@konkuk.ac.kr (S.K.); hds49@konkuk.ac.kr (D.H.); qwer92224@konkuk.ac.kr (T.K.);
exercise@konkuk.ac.kr (K.L.); parkhy1980@konkuk.ac.kr (H.-Y.P.); kswrha@konkuk.ac.kr (S.-W.K.)
2 Physical Activity and Performance Institute (PAPI), Konkuk University, Seoul 05029, Republic of Korea
3 Department of Physical Education, Konkuk University, Seoul 05029, Republic of Korea
* Correspondence: kimpro@konkuk.ac.kr; Tel.: +82-10-2049-6034

## Slide 2
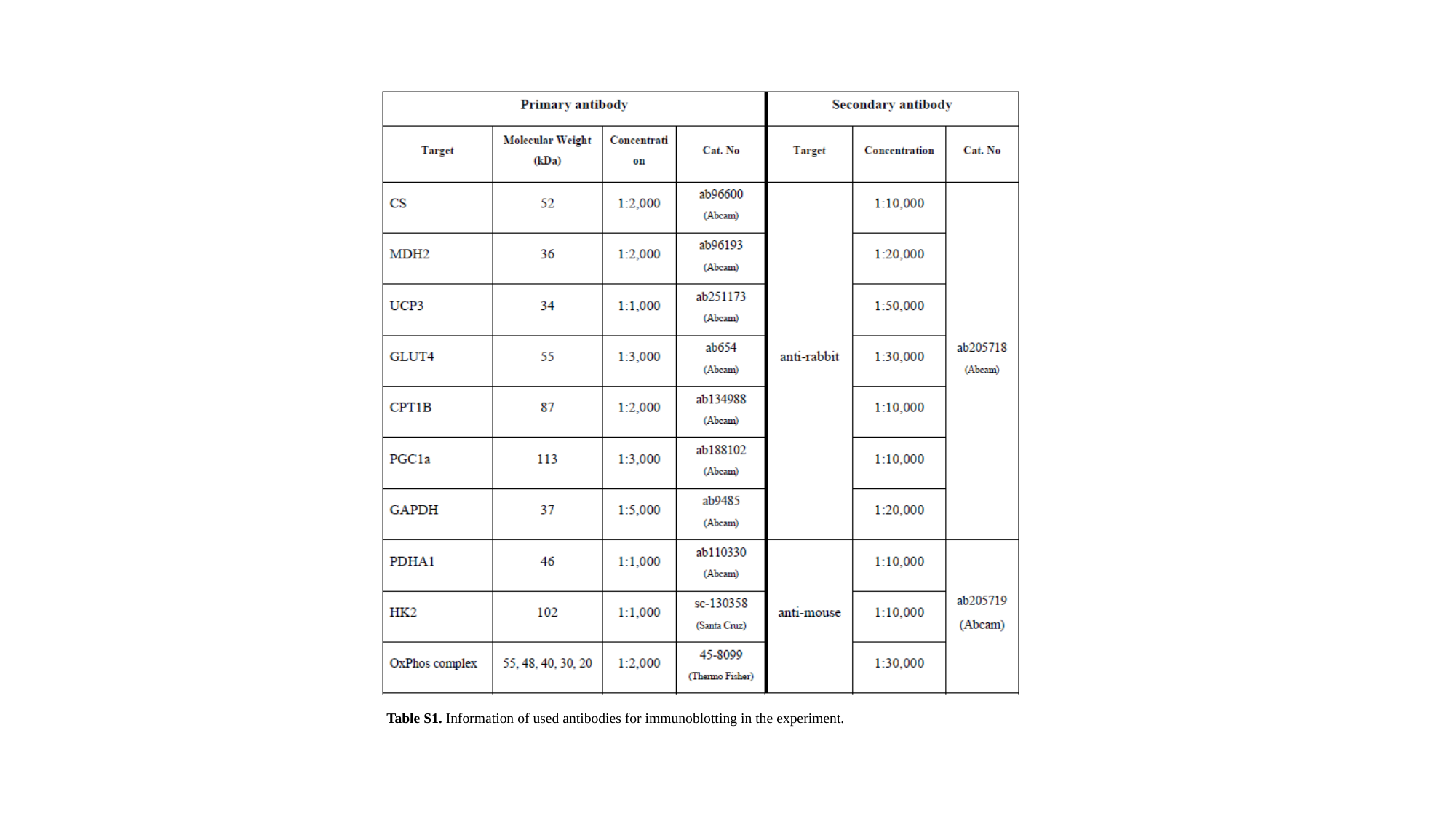

Table S1. Information of used antibodies for immunoblotting in the experiment.

## Slide 3
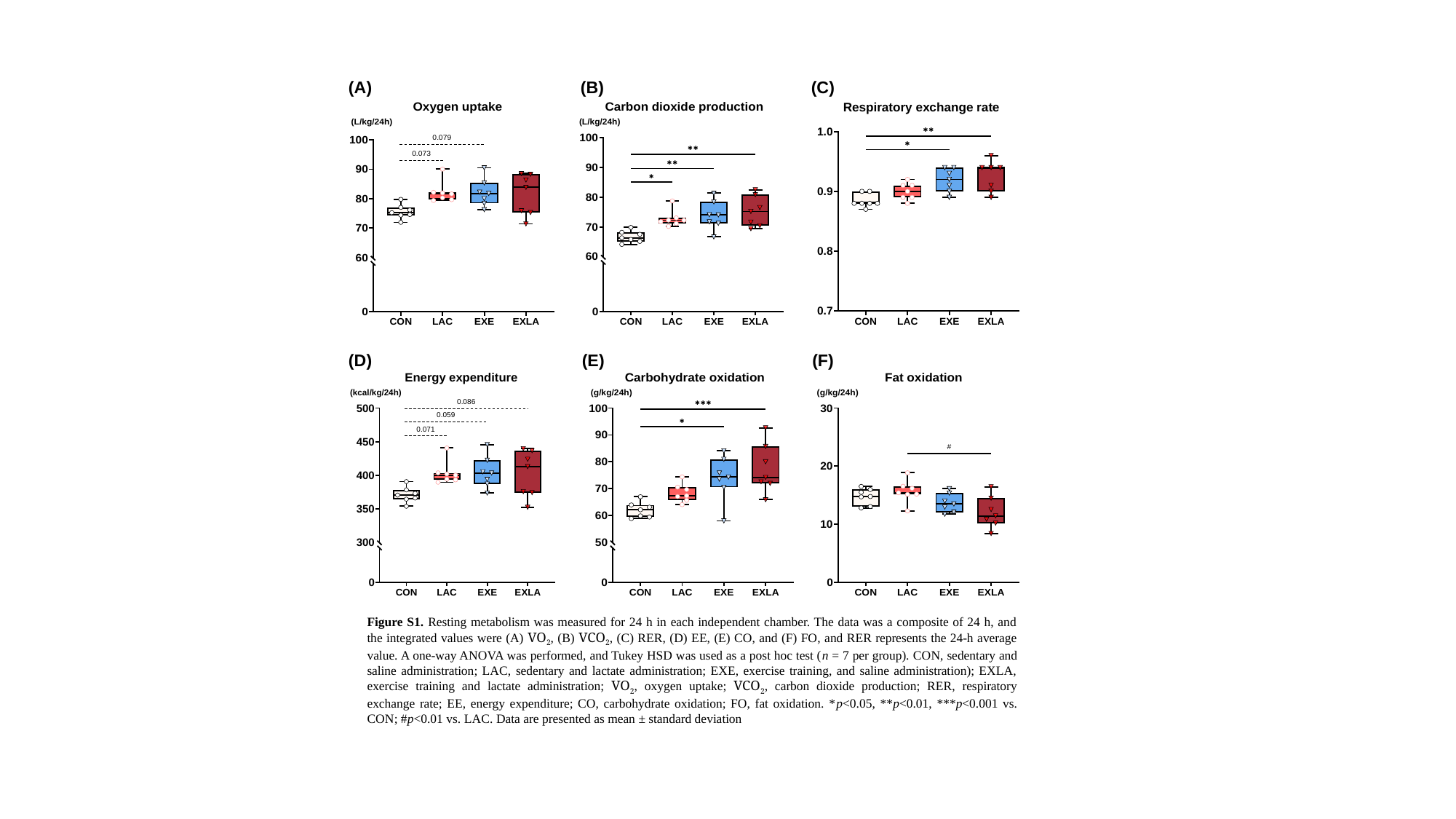

(A)
(B)
(C)
(D)
(E)
(F)
Figure S1. Resting metabolism was measured for 24 h in each independent chamber. The data was a composite of 24 h, and the integrated values were (A) VO2, (B) VCO2, (C) RER, (D) EE, (E) CO, and (F) FO, and RER represents the 24-h average value. A one-way ANOVA was performed, and Tukey HSD was used as a post hoc test (n = 7 per group). CON, sedentary and saline administration; LAC, sedentary and lactate administration; EXE, exercise training, and saline administration); EXLA, exercise training and lactate administration; VO2, oxygen uptake; VCO2, carbon dioxide production; RER, respiratory exchange rate; EE, energy expenditure; CO, carbohydrate oxidation; FO, fat oxidation. *p<0.05, **p<0.01, ***p<0.001 vs. CON; #p<0.01 vs. LAC. Data are presented as mean ± standard deviation

## Slide 4
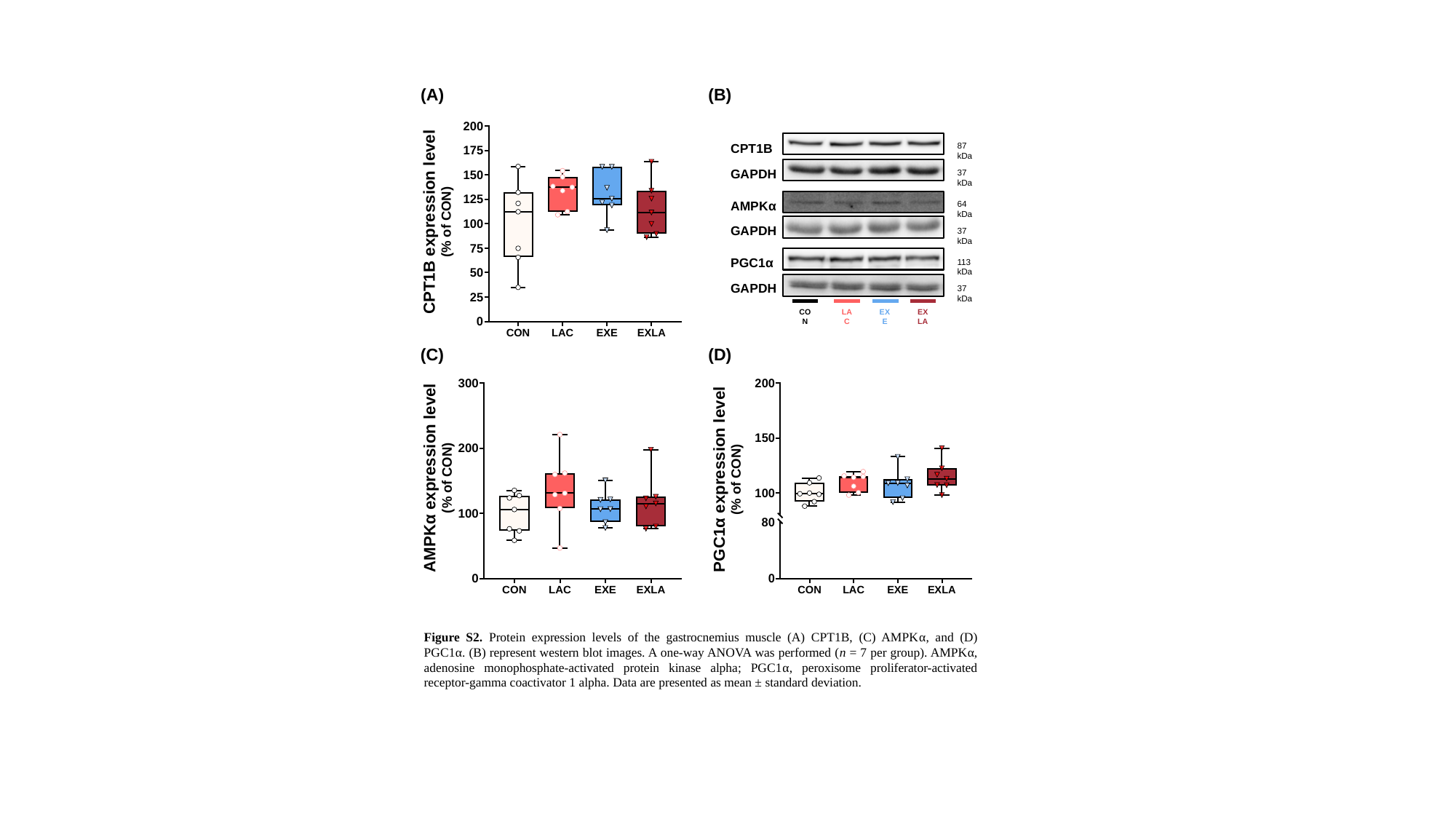

(A)
(B)
CPT1B
87 kDa
GAPDH
37 kDa
AMPKα
64 kDa
GAPDH
37 kDa
PGC1α
113 kDa
GAPDH
37 kDa
CON
LAC
EXE
EXLA
(C)
(D)
Figure S2. Protein expression levels of the gastrocnemius muscle (A) CPT1B, (C) AMPKα, and (D) PGC1α. (B) represent western blot images. A one-way ANOVA was performed (n = 7 per group). AMPKα, adenosine monophosphate-activated protein kinase alpha; PGC1α, peroxisome proliferator-activated receptor-gamma coactivator 1 alpha. Data are presented as mean ± standard deviation.

## Slide 5
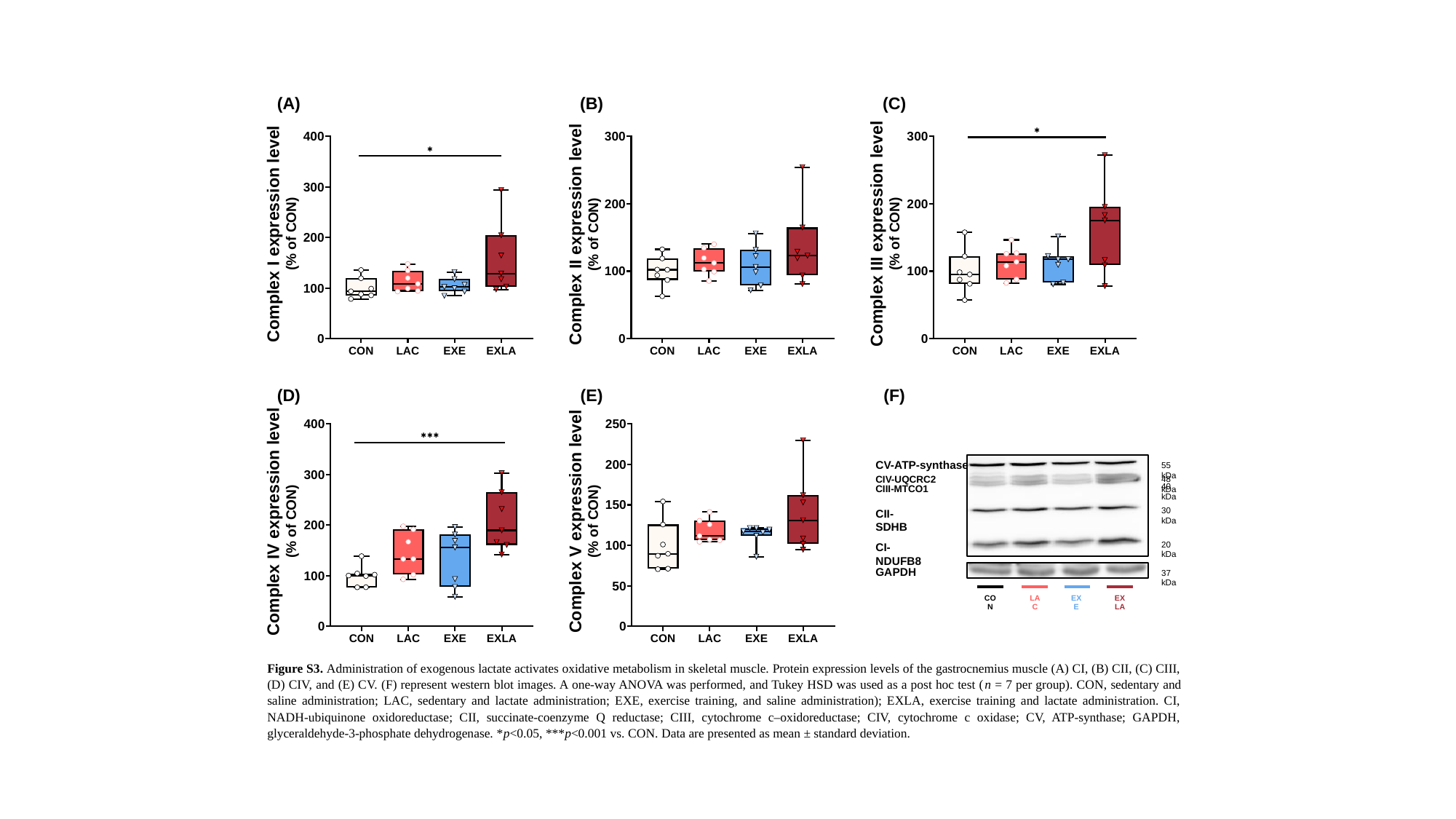

(A)
(B)
(C)
(D)
(E)
(F)
CV-ATP-synthase
55 kDa
CIV-UQCRC2
48 kDa
40 kDa
CIII-MTCO1
30 kDa
CII-SDHB
20 kDa
CI-NDUFB8
GAPDH
37 kDa
CON
LAC
EXE
EXLA
Figure S3. Administration of exogenous lactate activates oxidative metabolism in skeletal muscle. Protein expression levels of the gastrocnemius muscle (A) CI, (B) CII, (C) CIII, (D) CIV, and (E) CV. (F) represent western blot images. A one-way ANOVA was performed, and Tukey HSD was used as a post hoc test (n = 7 per group). CON, sedentary and saline administration; LAC, sedentary and lactate administration; EXE, exercise training, and saline administration); EXLA, exercise training and lactate administration. CI, NADH-ubiquinone oxidoreductase; CII, succinate-coenzyme Q reductase; CIII, cytochrome c–oxidoreductase; CIV, cytochrome c oxidase; CV, ATP-synthase; GAPDH, glyceraldehyde-3-phosphate dehydrogenase. *p<0.05, ***p<0.001 vs. CON. Data are presented as mean ± standard deviation.

## Slide 6
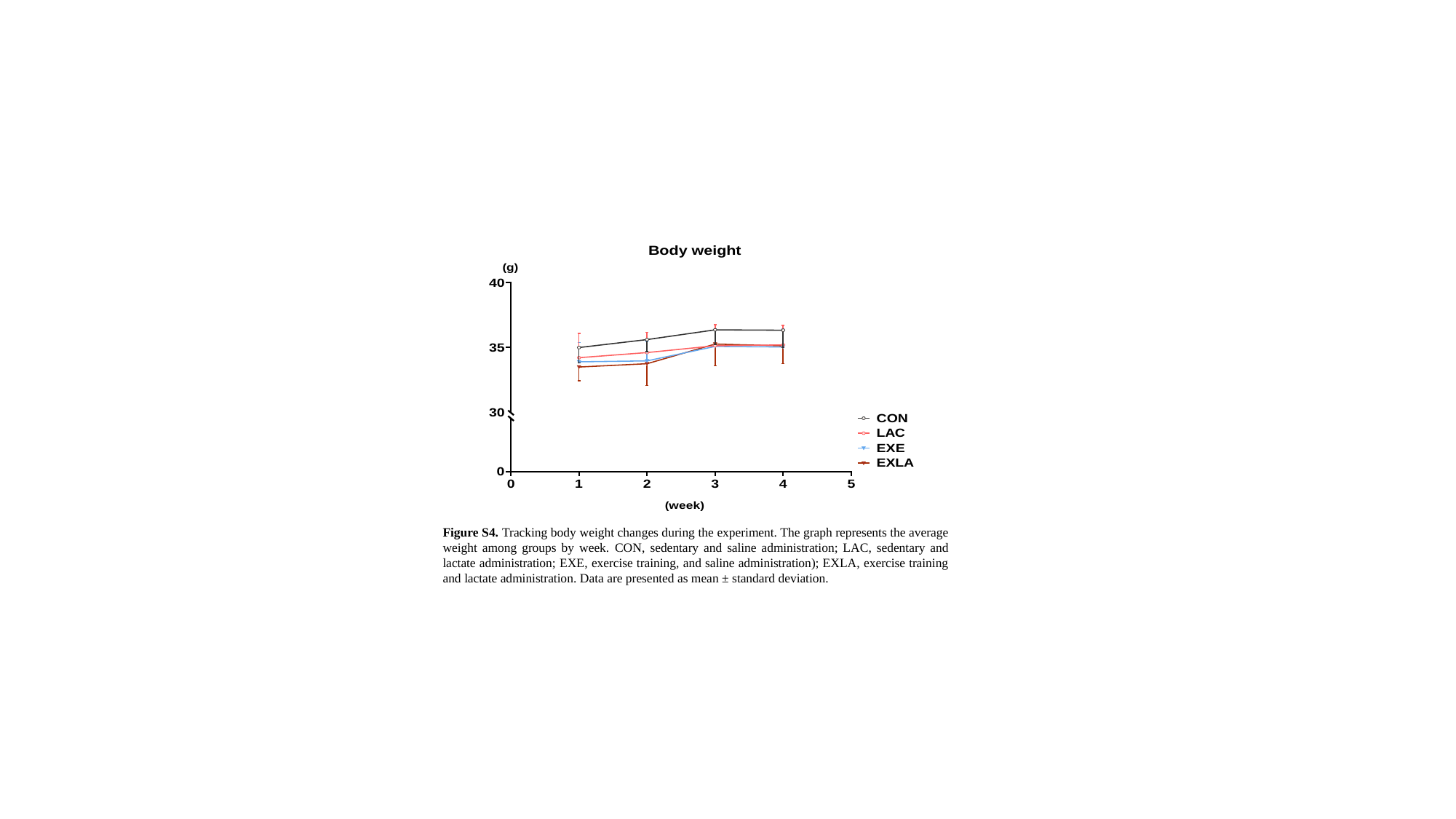

Figure S4. Tracking body weight changes during the experiment. The graph represents the average weight among groups by week. CON, sedentary and saline administration; LAC, sedentary and lactate administration; EXE, exercise training, and saline administration); EXLA, exercise training and lactate administration. Data are presented as mean ± standard deviation.

## Slide 7
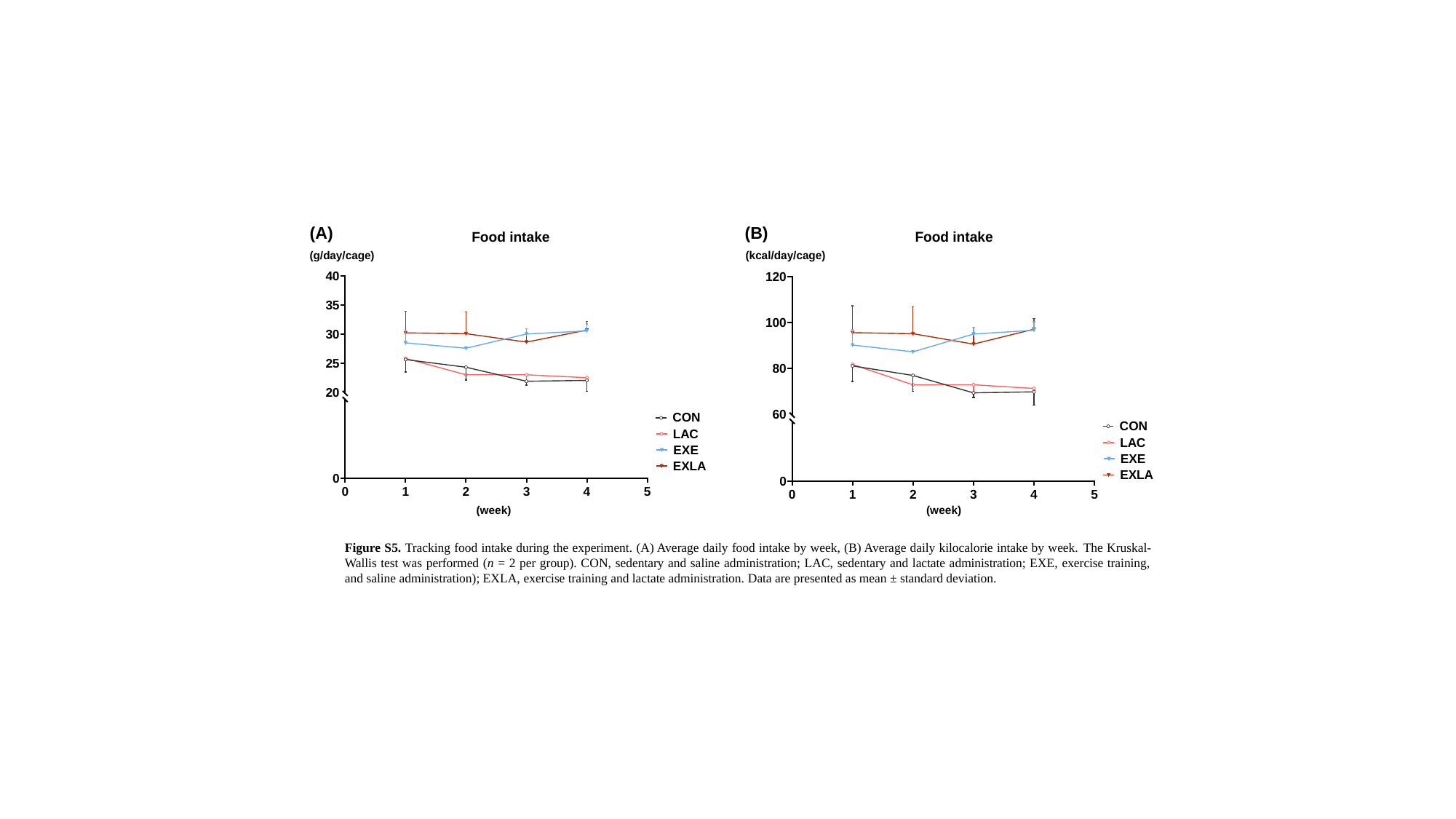

(A)
(B)
Figure S5. Tracking food intake during the experiment. (A) Average daily food intake by week, (B) Average daily kilocalorie intake by week. The Kruskal-Wallis test was performed (n = 2 per group). CON, sedentary and saline administration; LAC, sedentary and lactate administration; EXE, exercise training, and saline administration); EXLA, exercise training and lactate administration. Data are presented as mean ± standard deviation.

## Slide 8
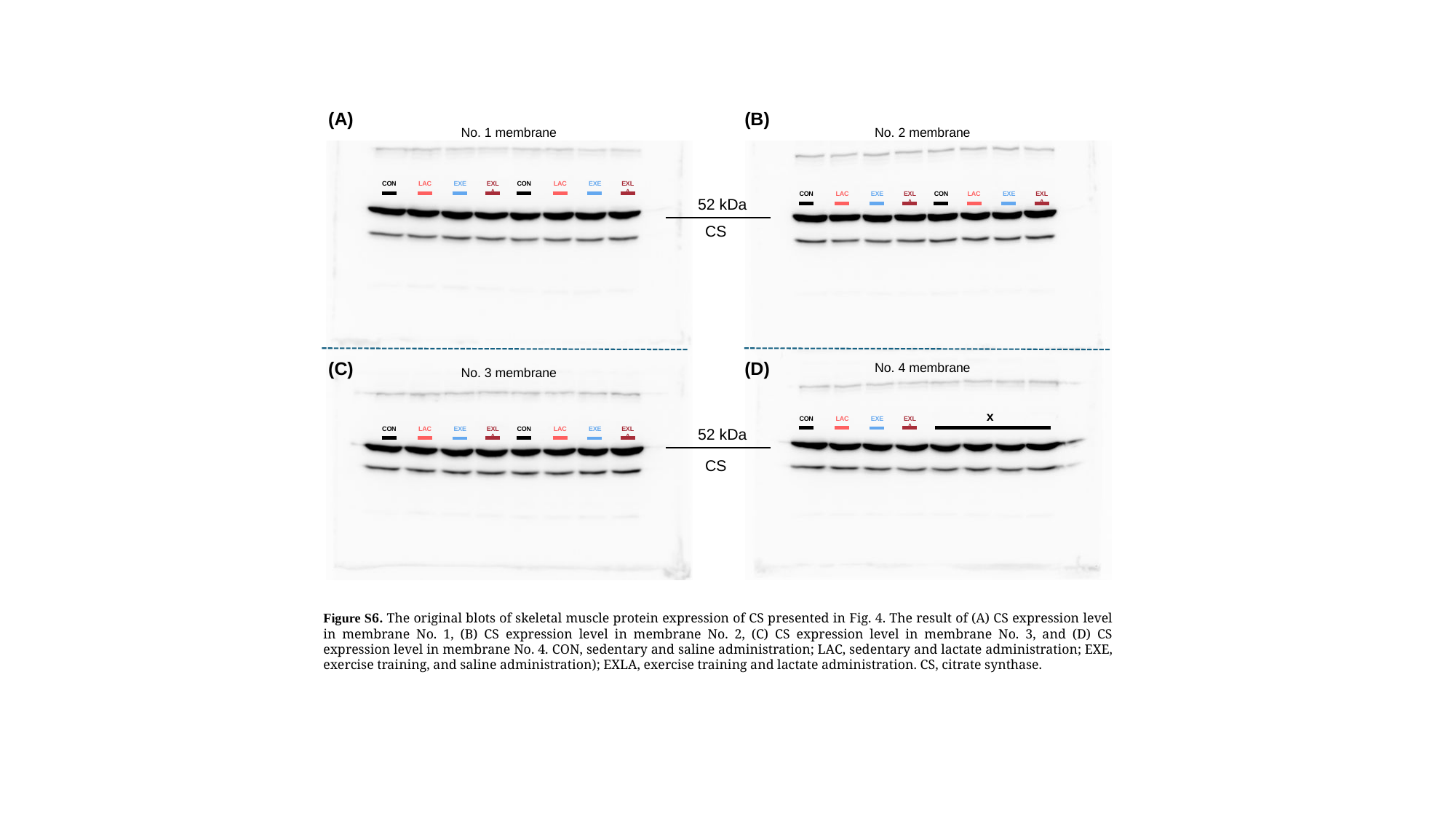

(A)
(B)
No. 1 membrane
No. 2 membrane
EXE
EXE
EXLA
EXLA
CON
LAC
CON
LAC
EXE
EXE
EXLA
EXLA
CON
LAC
CON
LAC
52 kDa
CS
(C)
(D)
No. 4 membrane
No. 3 membrane
x
EXE
EXLA
CON
LAC
EXE
EXE
52 kDa
EXLA
EXLA
CON
LAC
CON
LAC
CS
Figure S6. The original blots of skeletal muscle protein expression of CS presented in Fig. 4. The result of (A) CS expression level in membrane No. 1, (B) CS expression level in membrane No. 2, (C) CS expression level in membrane No. 3, and (D) CS expression level in membrane No. 4. CON, sedentary and saline administration; LAC, sedentary and lactate administration; EXE, exercise training, and saline administration); EXLA, exercise training and lactate administration. CS, citrate synthase.

## Slide 9
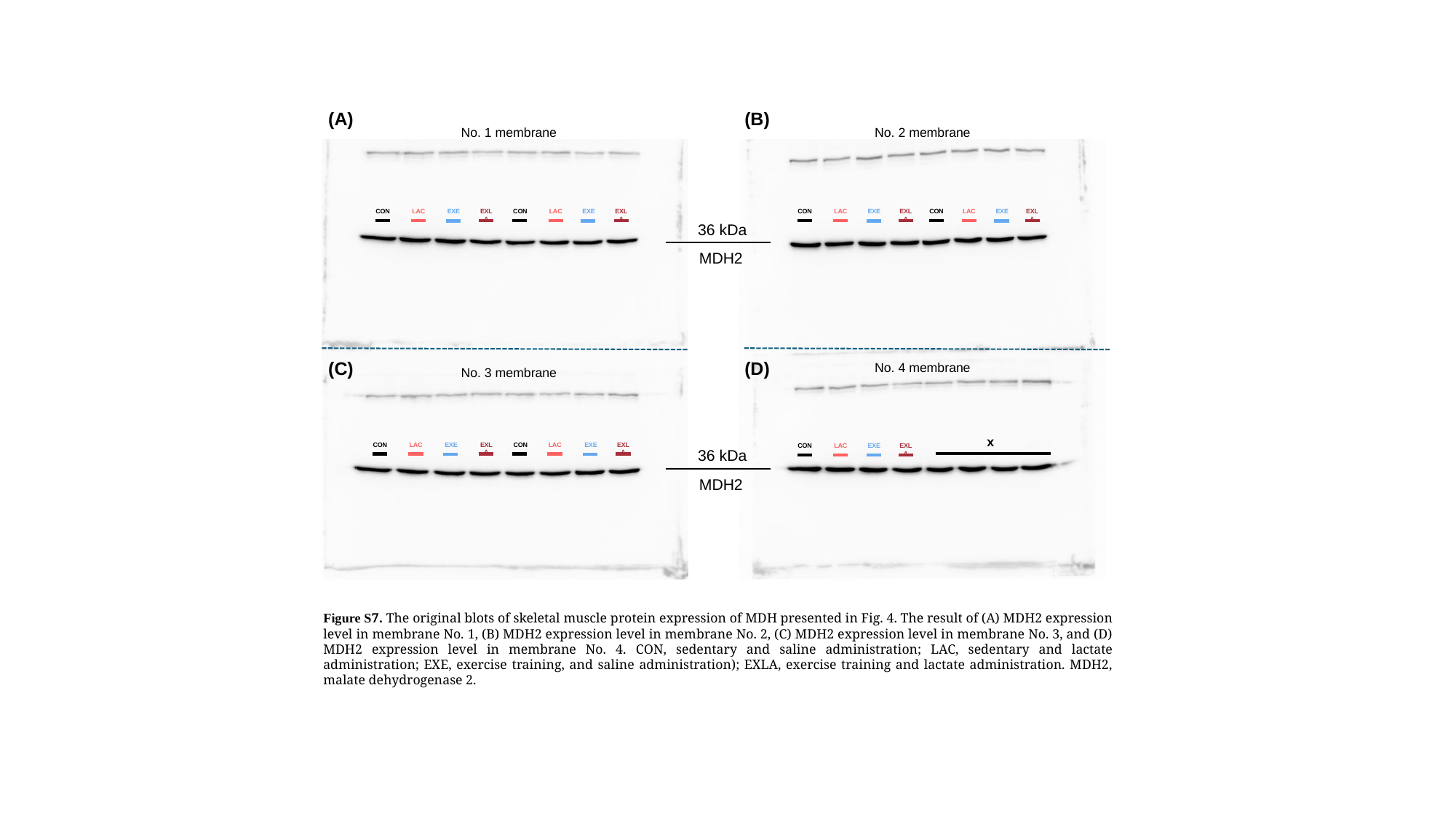

(A)
(B)
No. 1 membrane
No. 2 membrane
EXE
EXE
EXE
EXE
EXLA
EXLA
EXLA
EXLA
CON
LAC
CON
LAC
CON
LAC
CON
LAC
36 kDa
MDH2
(C)
(D)
No. 4 membrane
No. 3 membrane
x
EXE
EXE
EXLA
EXLA
CON
LAC
CON
LAC
EXE
EXLA
CON
LAC
36 kDa
MDH2
Figure S7. The original blots of skeletal muscle protein expression of MDH presented in Fig. 4. The result of (A) MDH2 expression level in membrane No. 1, (B) MDH2 expression level in membrane No. 2, (C) MDH2 expression level in membrane No. 3, and (D) MDH2 expression level in membrane No. 4. CON, sedentary and saline administration; LAC, sedentary and lactate administration; EXE, exercise training, and saline administration); EXLA, exercise training and lactate administration. MDH2, malate dehydrogenase 2.

## Slide 10
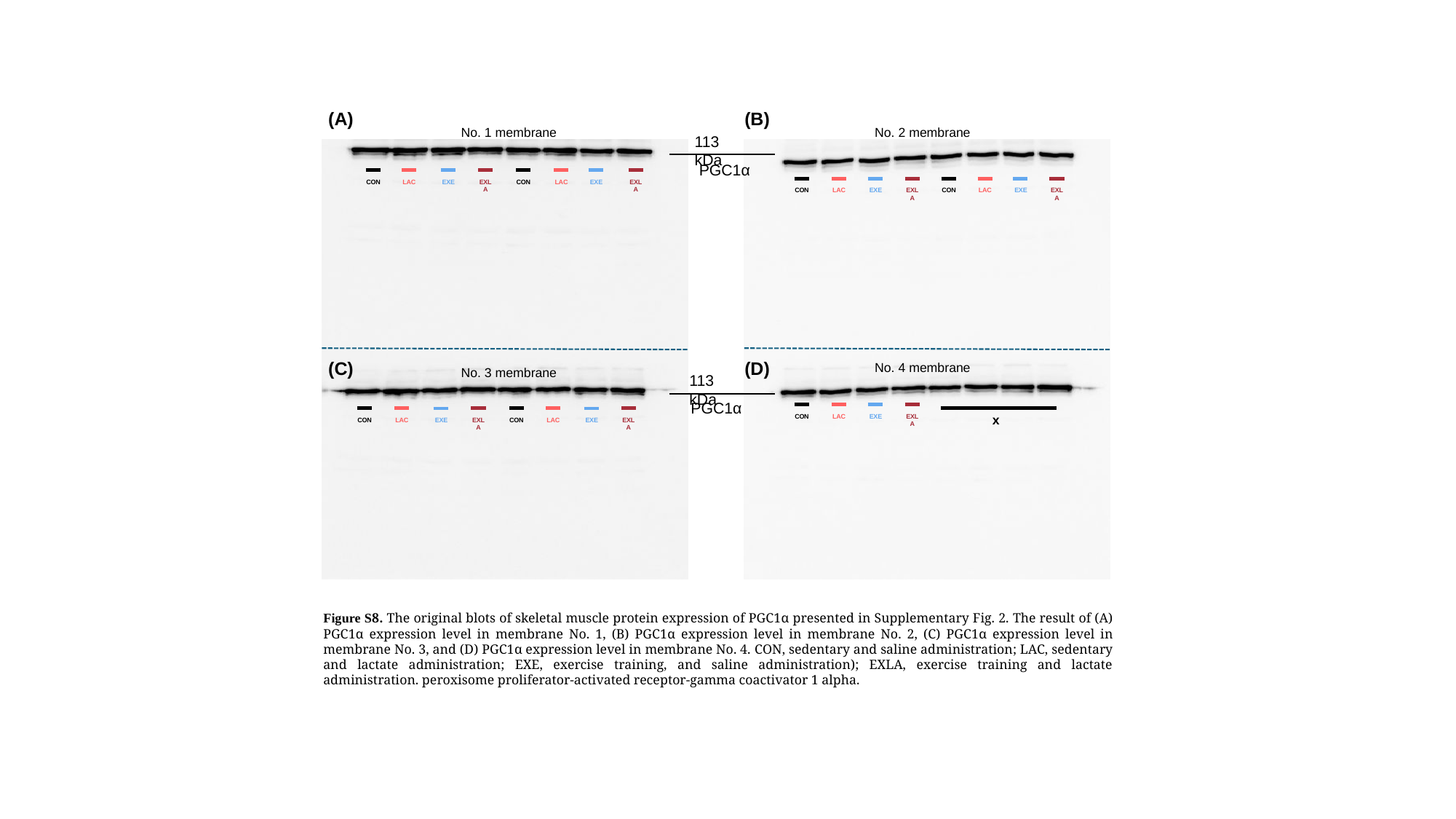

(A)
(B)
No. 1 membrane
No. 2 membrane
113 kDa
PGC1α
CON
LAC
EXE
EXLA
CON
LAC
EXE
EXLA
CON
LAC
EXE
EXLA
CON
LAC
EXE
EXLA
(C)
(D)
No. 4 membrane
No. 3 membrane
113 kDa
PGC1α
CON
LAC
EXE
EXLA
x
CON
LAC
EXE
EXLA
CON
LAC
EXE
EXLA
Figure S8. The original blots of skeletal muscle protein expression of PGC1α presented in Supplementary Fig. 2. The result of (A) PGC1α expression level in membrane No. 1, (B) PGC1α expression level in membrane No. 2, (C) PGC1α expression level in membrane No. 3, and (D) PGC1α expression level in membrane No. 4. CON, sedentary and saline administration; LAC, sedentary and lactate administration; EXE, exercise training, and saline administration); EXLA, exercise training and lactate administration. peroxisome proliferator-activated receptor-gamma coactivator 1 alpha.

## Slide 11
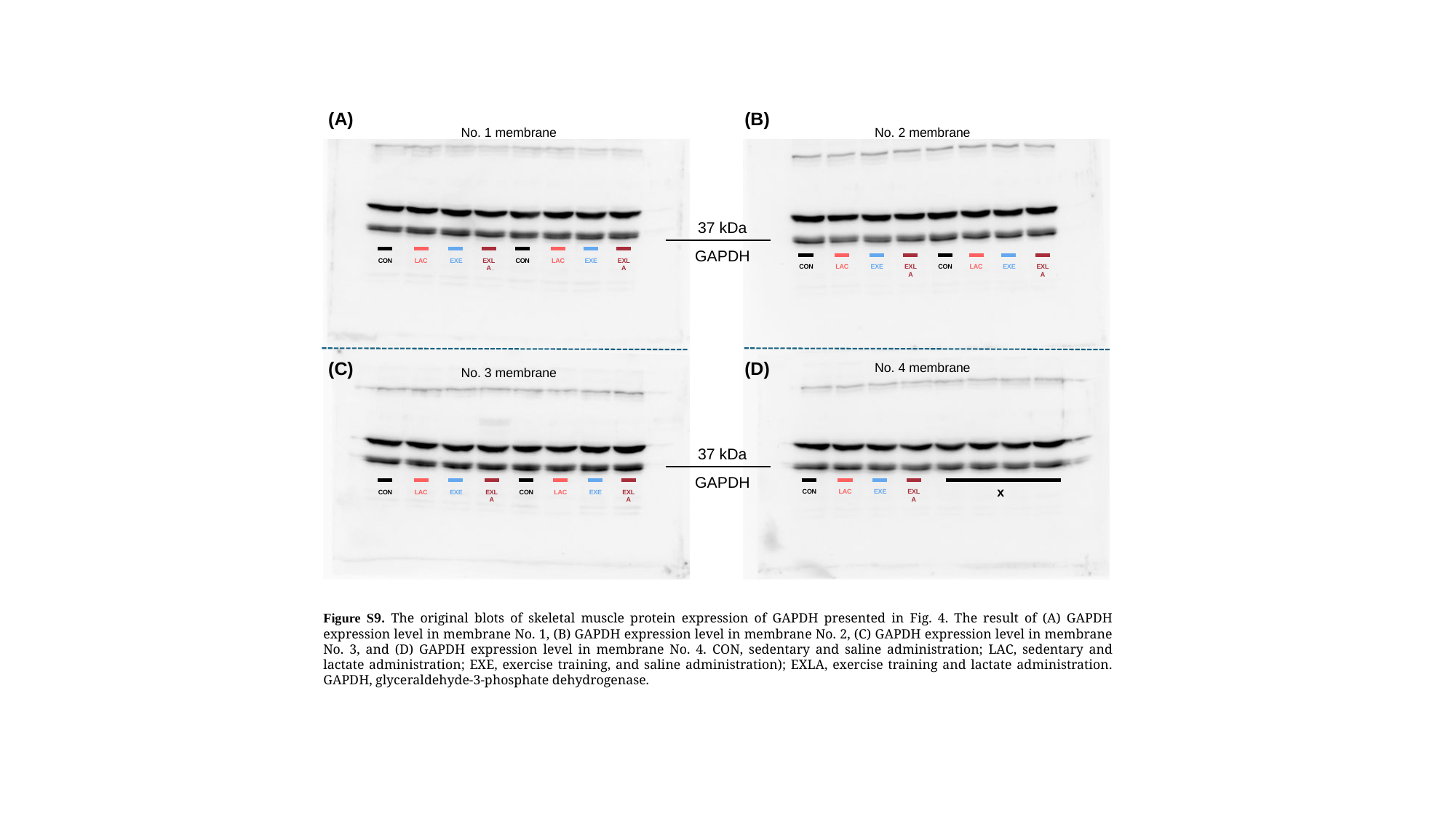

(A)
(B)
No. 1 membrane
No. 2 membrane
37 kDa
GAPDH
CON
LAC
EXE
EXLA
CON
LAC
EXE
EXLA
CON
LAC
EXE
EXLA
CON
LAC
EXE
EXLA
(C)
(D)
No. 4 membrane
No. 3 membrane
37 kDa
GAPDH
x
CON
LAC
EXE
EXLA
CON
LAC
EXE
EXLA
CON
LAC
EXE
EXLA
Figure S9. The original blots of skeletal muscle protein expression of GAPDH presented in Fig. 4. The result of (A) GAPDH expression level in membrane No. 1, (B) GAPDH expression level in membrane No. 2, (C) GAPDH expression level in membrane No. 3, and (D) GAPDH expression level in membrane No. 4. CON, sedentary and saline administration; LAC, sedentary and lactate administration; EXE, exercise training, and saline administration); EXLA, exercise training and lactate administration. GAPDH, glyceraldehyde-3-phosphate dehydrogenase.

## Slide 12
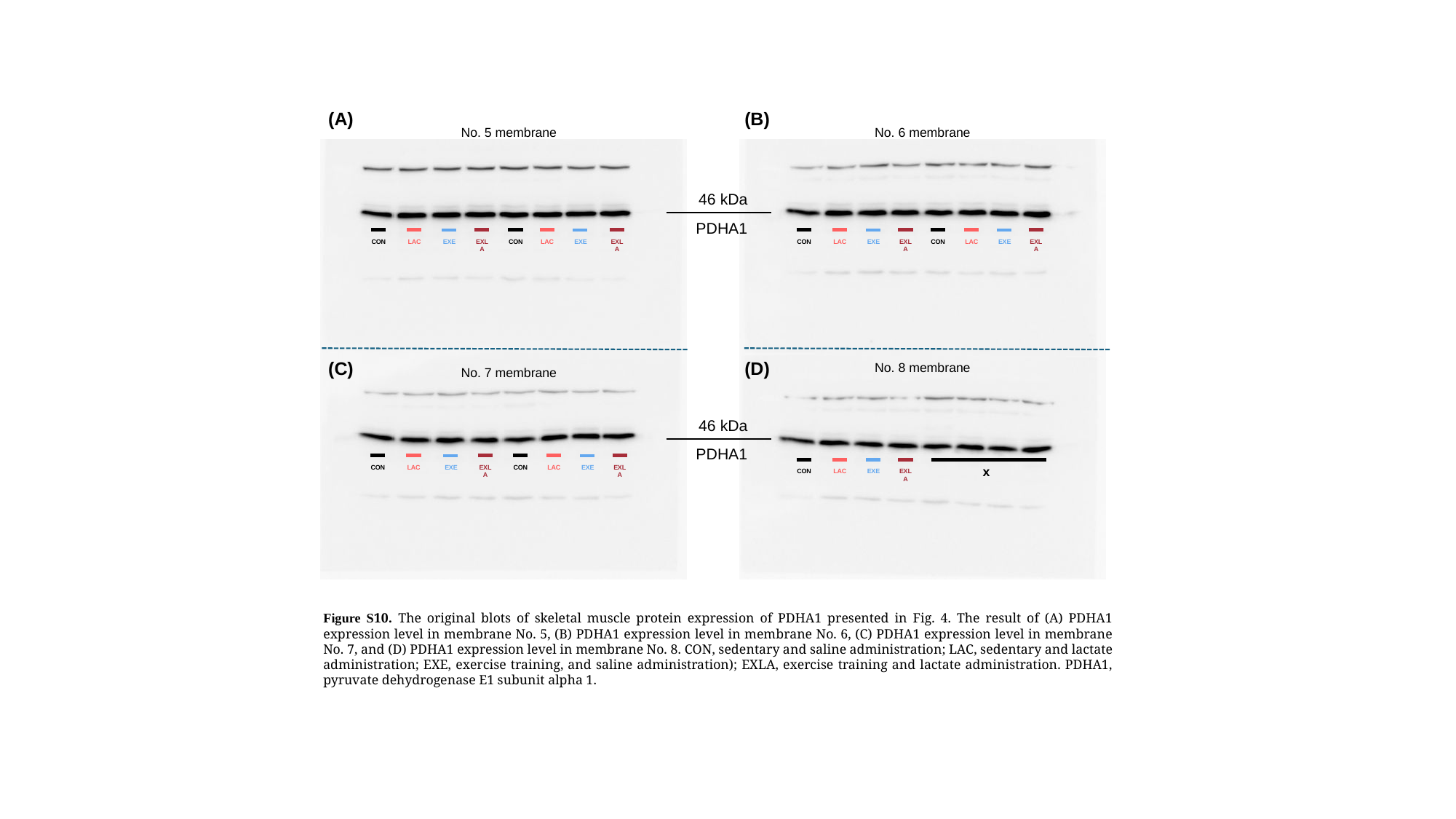

(A)
(B)
No. 5 membrane
No. 6 membrane
46 kDa
PDHA1
CON
LAC
EXE
EXLA
CON
LAC
EXE
EXLA
CON
LAC
EXE
EXLA
CON
LAC
EXE
EXLA
(C)
(D)
No. 8 membrane
No. 7 membrane
46 kDa
PDHA1
CON
LAC
EXE
EXLA
CON
LAC
EXE
EXLA
x
CON
LAC
EXE
EXLA
Figure S10. The original blots of skeletal muscle protein expression of PDHA1 presented in Fig. 4. The result of (A) PDHA1 expression level in membrane No. 5, (B) PDHA1 expression level in membrane No. 6, (C) PDHA1 expression level in membrane No. 7, and (D) PDHA1 expression level in membrane No. 8. CON, sedentary and saline administration; LAC, sedentary and lactate administration; EXE, exercise training, and saline administration); EXLA, exercise training and lactate administration. PDHA1, pyruvate dehydrogenase E1 subunit alpha 1.

## Slide 13
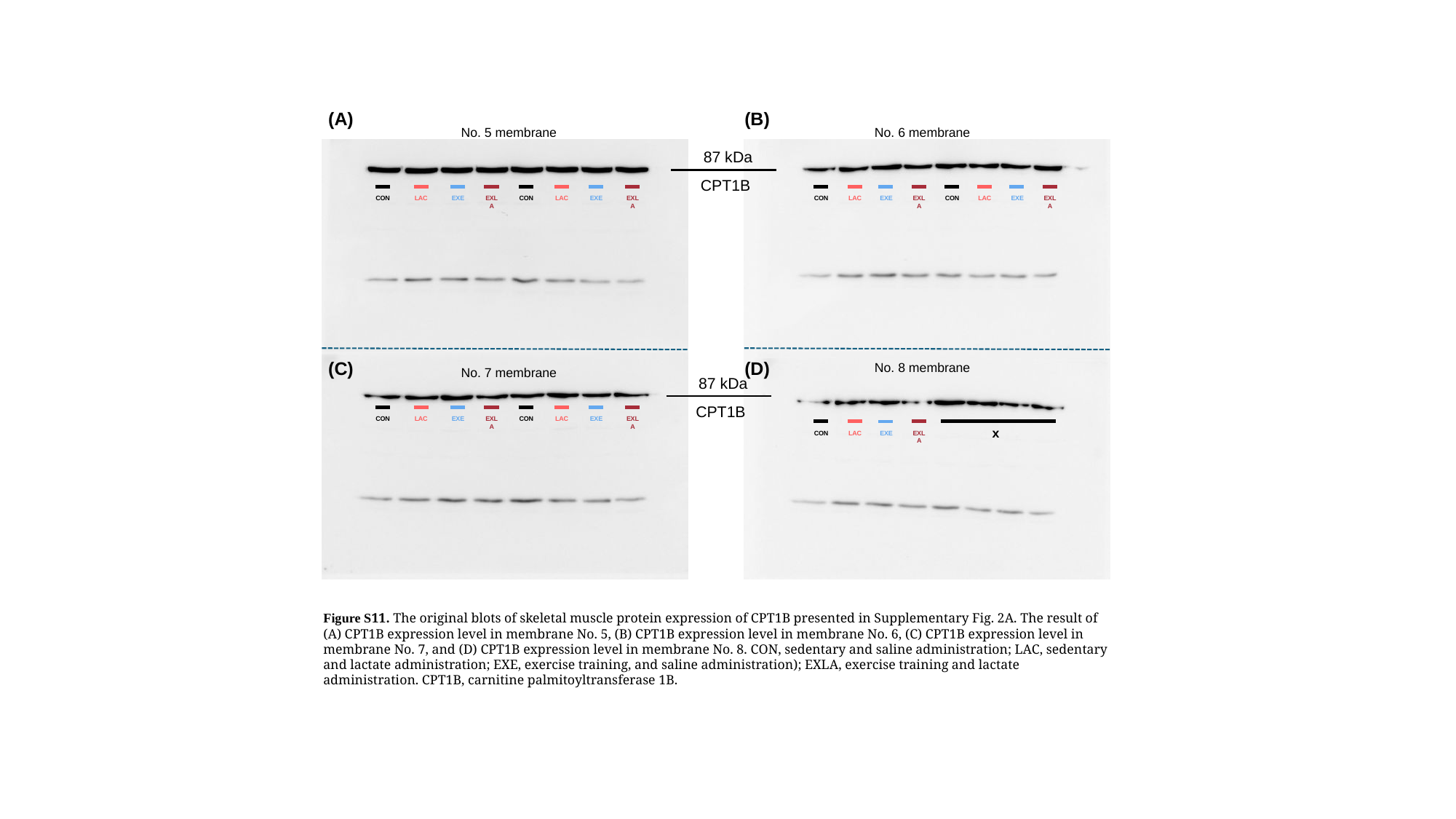

(A)
(B)
No. 5 membrane
No. 6 membrane
87 kDa
CPT1B
CON
LAC
EXE
EXLA
CON
LAC
EXE
EXLA
CON
LAC
EXE
EXLA
CON
LAC
EXE
EXLA
(C)
(D)
No. 8 membrane
No. 7 membrane
87 kDa
CPT1B
CON
LAC
EXE
EXLA
CON
LAC
EXE
EXLA
x
CON
LAC
EXE
EXLA
Figure S11. The original blots of skeletal muscle protein expression of CPT1B presented in Supplementary Fig. 2A. The result of (A) CPT1B expression level in membrane No. 5, (B) CPT1B expression level in membrane No. 6, (C) CPT1B expression level in membrane No. 7, and (D) CPT1B expression level in membrane No. 8. CON, sedentary and saline administration; LAC, sedentary and lactate administration; EXE, exercise training, and saline administration); EXLA, exercise training and lactate administration. CPT1B, carnitine palmitoyltransferase 1B.

## Slide 14
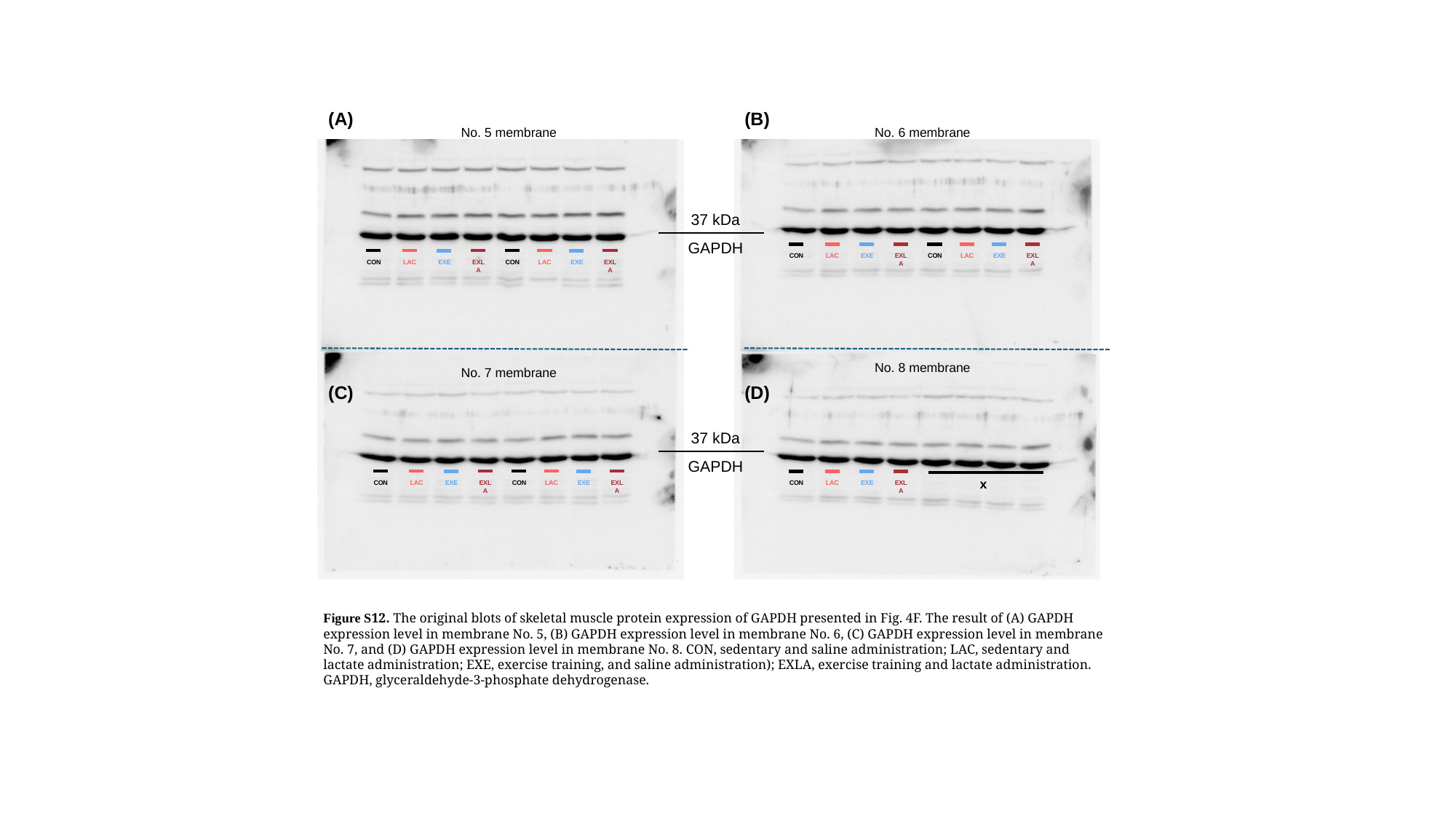

(A)
(B)
No. 5 membrane
No. 6 membrane
37 kDa
GAPDH
CON
LAC
EXE
EXLA
CON
LAC
EXE
EXLA
CON
LAC
EXE
EXLA
CON
LAC
EXE
EXLA
No. 8 membrane
No. 7 membrane
(C)
(D)
37 kDa
GAPDH
x
CON
LAC
EXE
EXLA
CON
LAC
EXE
EXLA
CON
LAC
EXE
EXLA
Figure S12. The original blots of skeletal muscle protein expression of GAPDH presented in Fig. 4F. The result of (A) GAPDH expression level in membrane No. 5, (B) GAPDH expression level in membrane No. 6, (C) GAPDH expression level in membrane No. 7, and (D) GAPDH expression level in membrane No. 8. CON, sedentary and saline administration; LAC, sedentary and lactate administration; EXE, exercise training, and saline administration); EXLA, exercise training and lactate administration. GAPDH, glyceraldehyde-3-phosphate dehydrogenase.

## Slide 15
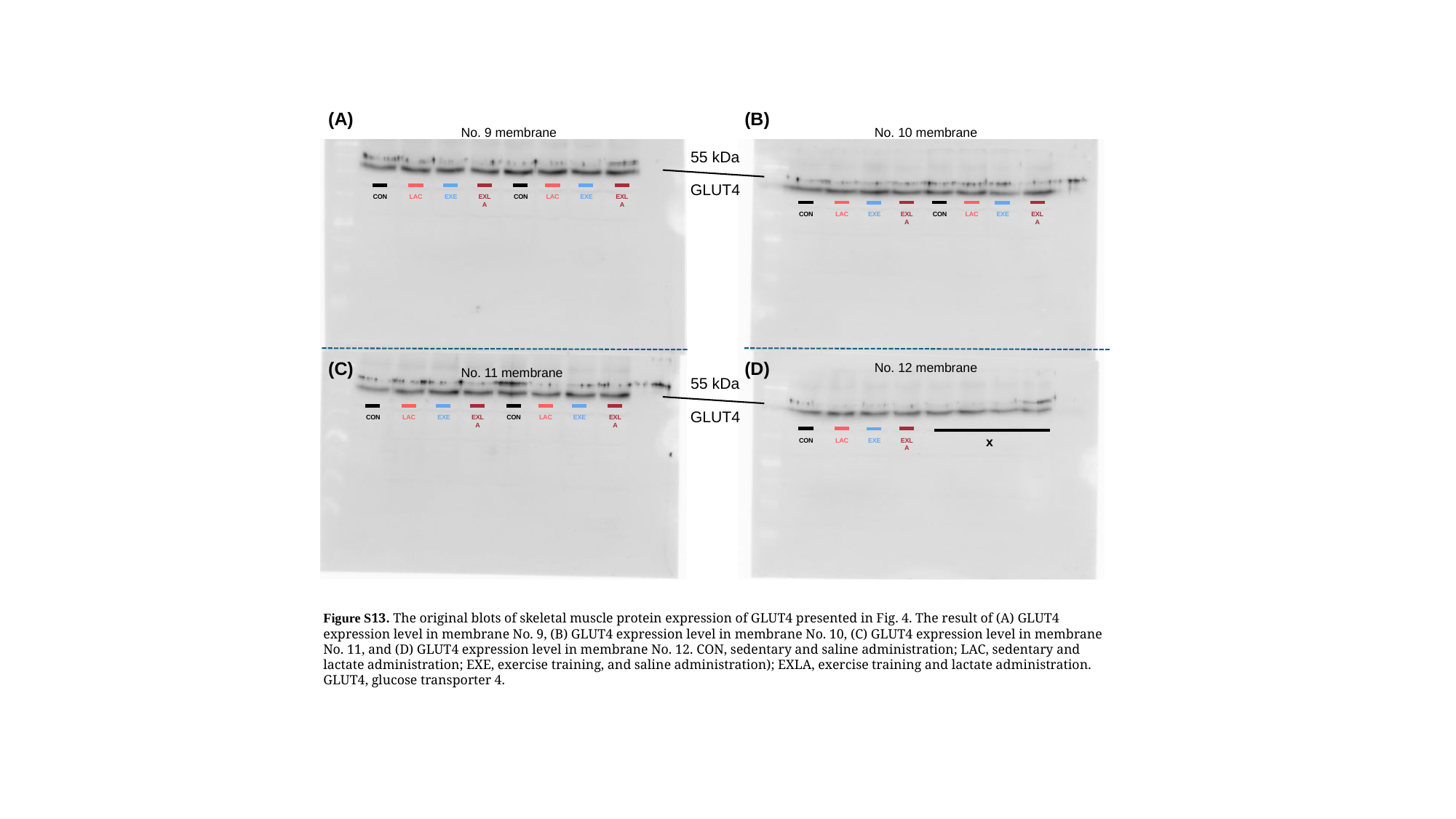

(A)
(B)
No. 9 membrane
No. 10 membrane
55 kDa
GLUT4
CON
LAC
EXE
EXLA
CON
LAC
EXE
EXLA
CON
LAC
EXE
EXLA
CON
LAC
EXE
EXLA
(C)
(D)
No. 12 membrane
No. 11 membrane
55 kDa
GLUT4
CON
LAC
EXE
EXLA
CON
LAC
EXE
EXLA
x
CON
LAC
EXE
EXLA
Figure S13. The original blots of skeletal muscle protein expression of GLUT4 presented in Fig. 4. The result of (A) GLUT4 expression level in membrane No. 9, (B) GLUT4 expression level in membrane No. 10, (C) GLUT4 expression level in membrane No. 11, and (D) GLUT4 expression level in membrane No. 12. CON, sedentary and saline administration; LAC, sedentary and lactate administration; EXE, exercise training, and saline administration); EXLA, exercise training and lactate administration. GLUT4, glucose transporter 4.

## Slide 16
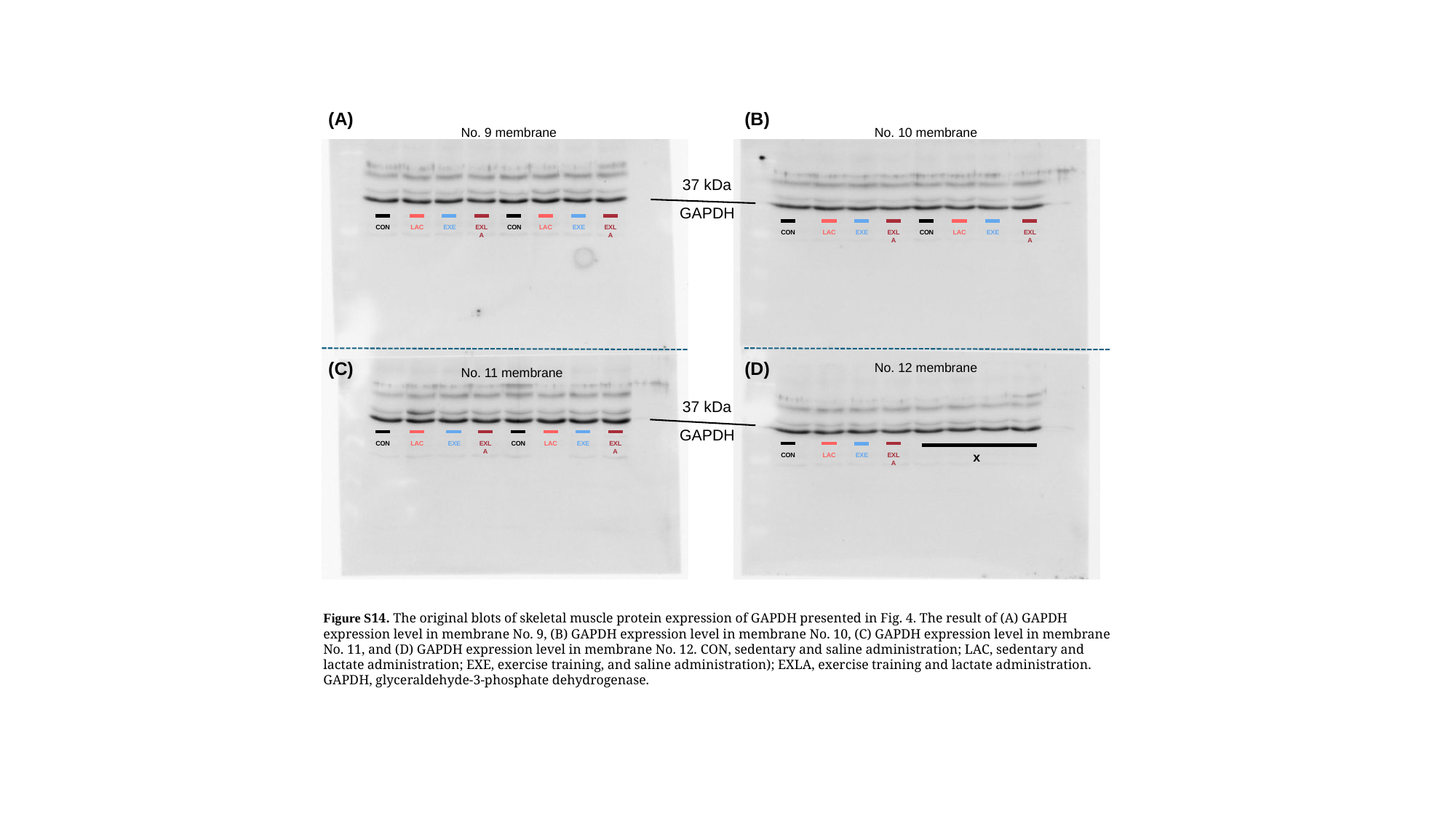

(A)
(B)
No. 9 membrane
No. 10 membrane
37 kDa
GAPDH
CON
LAC
EXE
EXLA
CON
LAC
EXE
EXLA
CON
LAC
EXE
EXLA
CON
LAC
EXE
EXLA
(C)
(D)
No. 12 membrane
No. 11 membrane
37 kDa
GAPDH
CON
LAC
EXE
EXLA
CON
LAC
EXE
EXLA
x
CON
LAC
EXE
EXLA
Figure S14. The original blots of skeletal muscle protein expression of GAPDH presented in Fig. 4. The result of (A) GAPDH expression level in membrane No. 9, (B) GAPDH expression level in membrane No. 10, (C) GAPDH expression level in membrane No. 11, and (D) GAPDH expression level in membrane No. 12. CON, sedentary and saline administration; LAC, sedentary and lactate administration; EXE, exercise training, and saline administration); EXLA, exercise training and lactate administration. GAPDH, glyceraldehyde-3-phosphate dehydrogenase.

## Slide 17
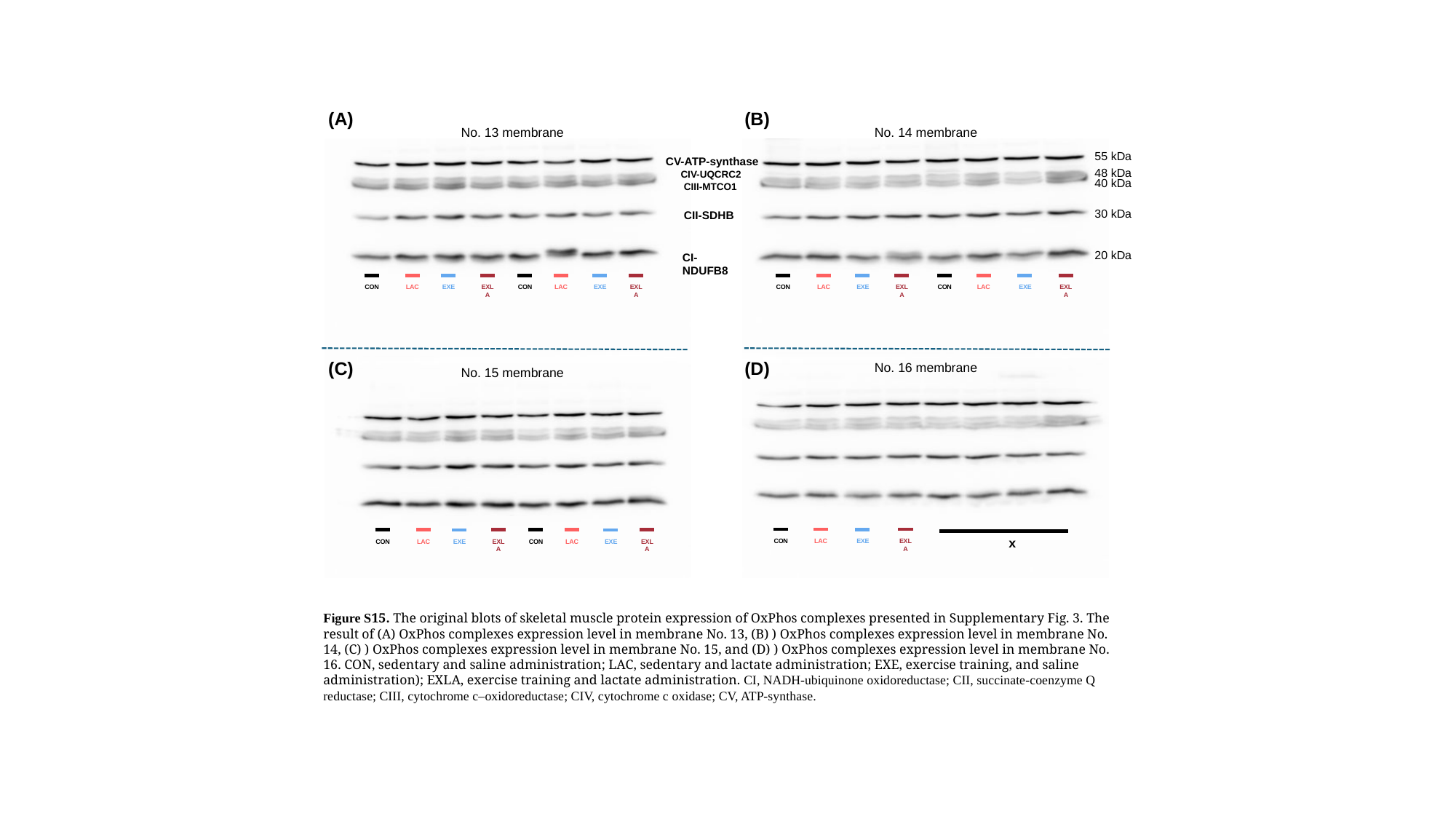

(A)
(B)
No. 13 membrane
No. 14 membrane
55 kDa
CV-ATP-synthase
48 kDa
CIV-UQCRC2
40 kDa
CIII-MTCO1
30 kDa
CII-SDHB
20 kDa
CI-NDUFB8
CON
LAC
EXE
EXLA
CON
LAC
EXE
EXLA
CON
LAC
EXE
EXLA
CON
LAC
EXE
EXLA
(C)
(D)
No. 16 membrane
No. 15 membrane
x
CON
LAC
EXE
EXLA
CON
LAC
EXE
EXLA
CON
LAC
EXE
EXLA
Figure S15. The original blots of skeletal muscle protein expression of OxPhos complexes presented in Supplementary Fig. 3. The result of (A) OxPhos complexes expression level in membrane No. 13, (B) ) OxPhos complexes expression level in membrane No. 14, (C) ) OxPhos complexes expression level in membrane No. 15, and (D) ) OxPhos complexes expression level in membrane No. 16. CON, sedentary and saline administration; LAC, sedentary and lactate administration; EXE, exercise training, and saline administration); EXLA, exercise training and lactate administration. CI, NADH-ubiquinone oxidoreductase; CII, succinate-coenzyme Q reductase; CIII, cytochrome c–oxidoreductase; CIV, cytochrome c oxidase; CV, ATP-synthase.

## Slide 18
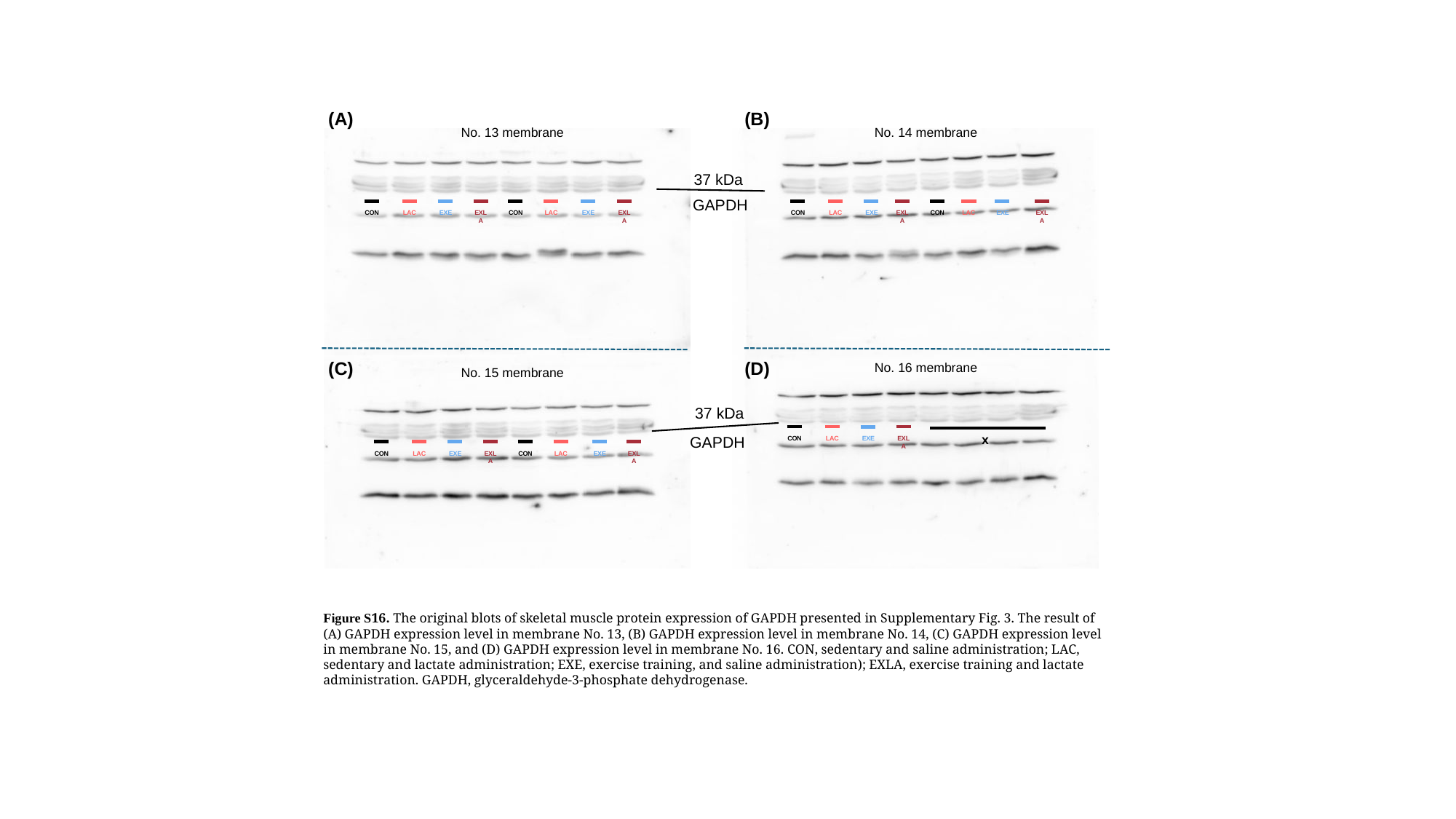

(A)
(B)
No. 13 membrane
No. 14 membrane
37 kDa
GAPDH
CON
LAC
EXE
EXLA
CON
LAC
EXE
EXLA
CON
LAC
EXE
EXLA
CON
LAC
EXE
EXLA
(C)
(D)
No. 16 membrane
No. 15 membrane
37 kDa
x
GAPDH
CON
LAC
EXE
EXLA
CON
LAC
EXE
EXLA
CON
LAC
EXE
EXLA
Figure S16. The original blots of skeletal muscle protein expression of GAPDH presented in Supplementary Fig. 3. The result of (A) GAPDH expression level in membrane No. 13, (B) GAPDH expression level in membrane No. 14, (C) GAPDH expression level in membrane No. 15, and (D) GAPDH expression level in membrane No. 16. CON, sedentary and saline administration; LAC, sedentary and lactate administration; EXE, exercise training, and saline administration); EXLA, exercise training and lactate administration. GAPDH, glyceraldehyde-3-phosphate dehydrogenase.

## Slide 19
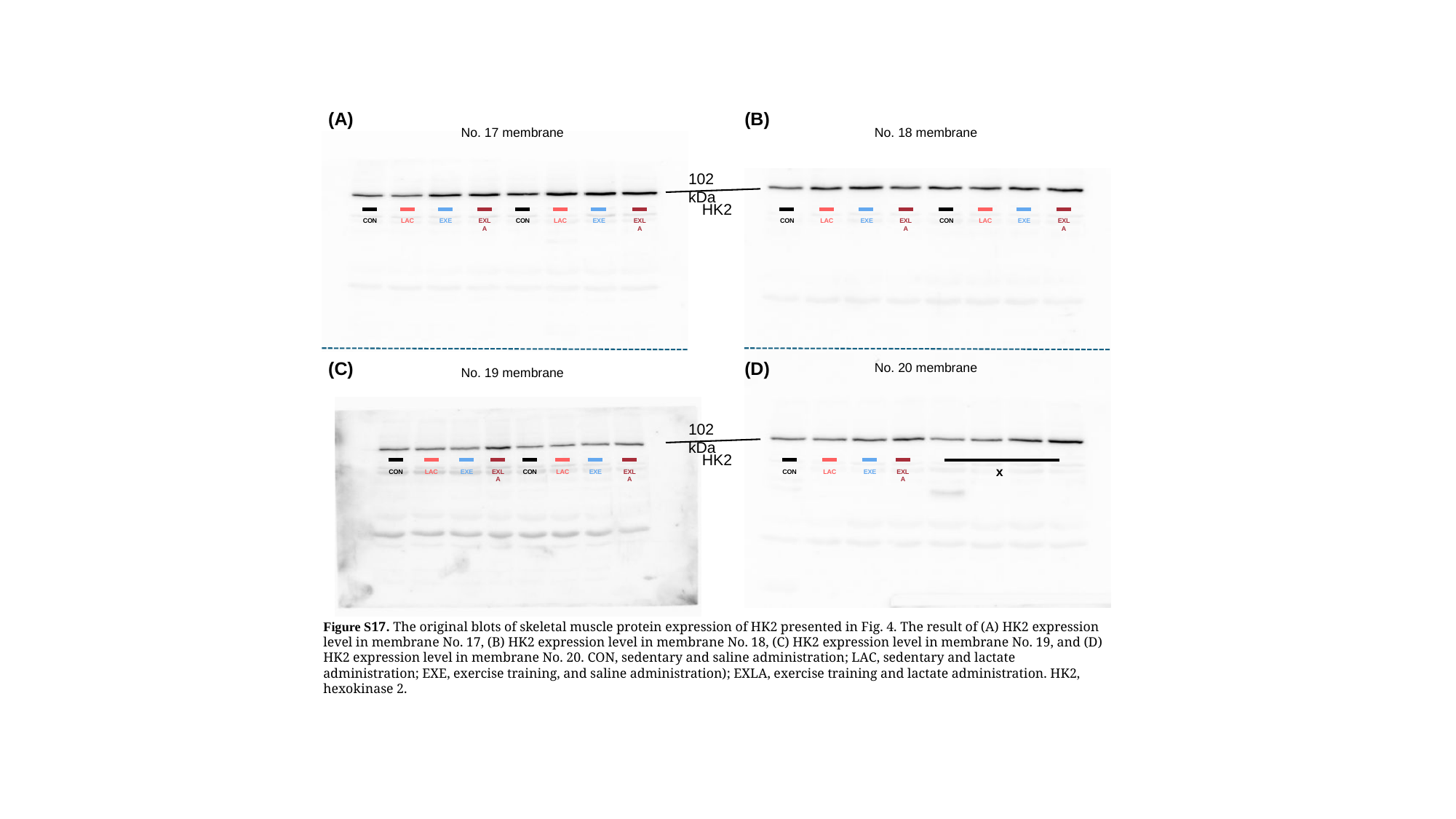

(A)
(B)
No. 17 membrane
No. 18 membrane
102 kDa
HK2
CON
LAC
EXE
EXLA
CON
LAC
EXE
EXLA
CON
LAC
EXE
EXLA
CON
LAC
EXE
EXLA
(C)
(D)
No. 20 membrane
No. 19 membrane
102 kDa
HK2
x
CON
LAC
EXE
EXLA
CON
LAC
EXE
EXLA
CON
LAC
EXE
EXLA
Figure S17. The original blots of skeletal muscle protein expression of HK2 presented in Fig. 4. The result of (A) HK2 expression level in membrane No. 17, (B) HK2 expression level in membrane No. 18, (C) HK2 expression level in membrane No. 19, and (D) HK2 expression level in membrane No. 20. CON, sedentary and saline administration; LAC, sedentary and lactate administration; EXE, exercise training, and saline administration); EXLA, exercise training and lactate administration. HK2, hexokinase 2.

## Slide 20
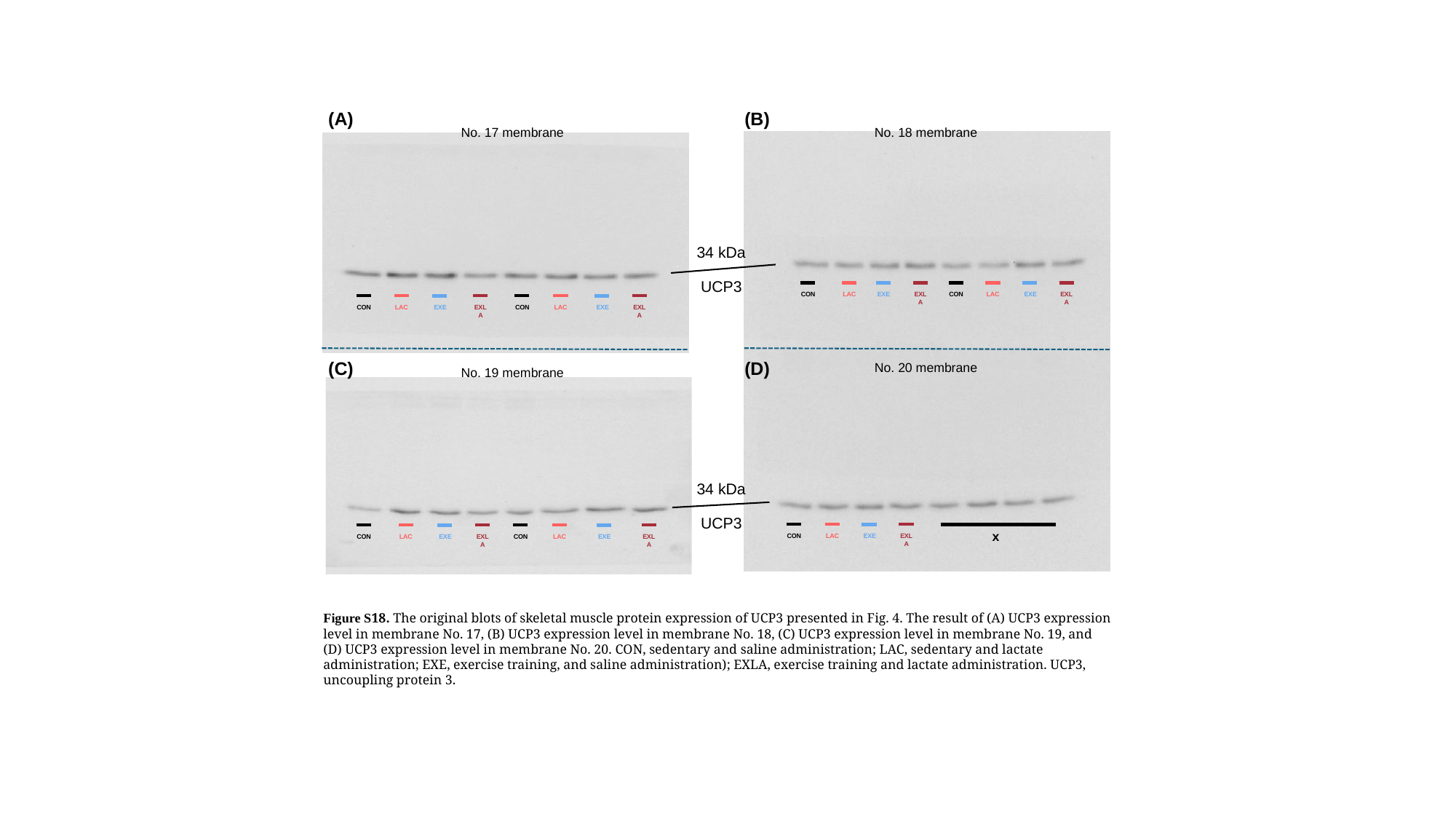

(A)
(B)
No. 17 membrane
No. 18 membrane
34 kDa
UCP3
CON
LAC
EXE
EXLA
CON
LAC
EXE
EXLA
CON
LAC
EXE
EXLA
CON
LAC
EXE
EXLA
(C)
(D)
No. 20 membrane
No. 19 membrane
34 kDa
UCP3
x
CON
LAC
EXE
EXLA
CON
LAC
EXE
EXLA
CON
LAC
EXE
EXLA
Figure S18. The original blots of skeletal muscle protein expression of UCP3 presented in Fig. 4. The result of (A) UCP3 expression level in membrane No. 17, (B) UCP3 expression level in membrane No. 18, (C) UCP3 expression level in membrane No. 19, and (D) UCP3 expression level in membrane No. 20. CON, sedentary and saline administration; LAC, sedentary and lactate administration; EXE, exercise training, and saline administration); EXLA, exercise training and lactate administration. UCP3, uncoupling protein 3.

## Slide 21
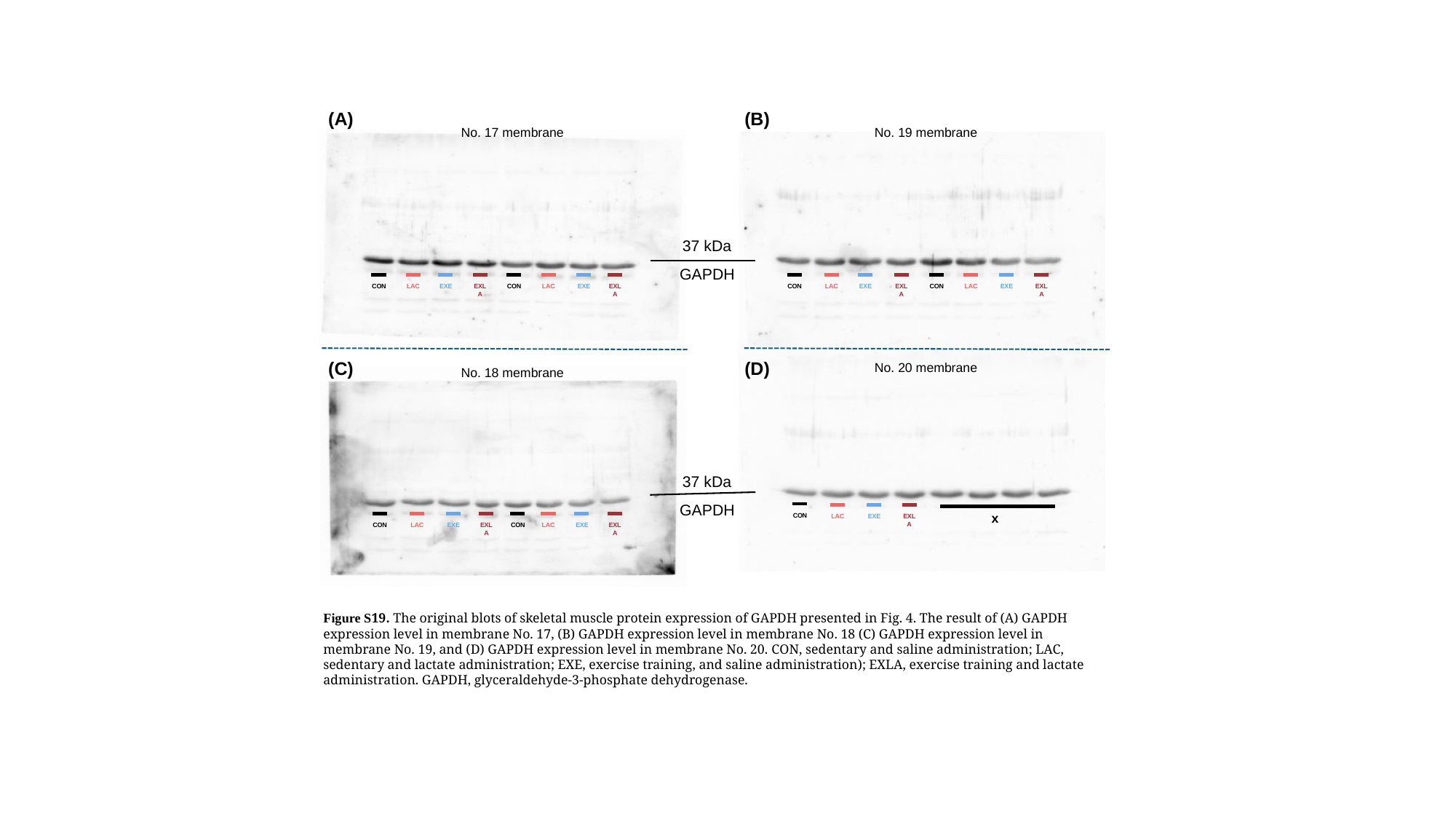

(A)
(B)
No. 17 membrane
No. 19 membrane
37 kDa
GAPDH
CON
LAC
EXE
EXLA
CON
LAC
EXE
EXLA
CON
LAC
EXE
EXLA
CON
LAC
EXE
EXLA
(C)
(D)
No. 20 membrane
No. 18 membrane
37 kDa
GAPDH
x
CON
LAC
EXE
EXLA
CON
LAC
EXE
EXLA
CON
LAC
EXE
EXLA
Figure S19. The original blots of skeletal muscle protein expression of GAPDH presented in Fig. 4. The result of (A) GAPDH expression level in membrane No. 17, (B) GAPDH expression level in membrane No. 18 (C) GAPDH expression level in membrane No. 19, and (D) GAPDH expression level in membrane No. 20. CON, sedentary and saline administration; LAC, sedentary and lactate administration; EXE, exercise training, and saline administration); EXLA, exercise training and lactate administration. GAPDH, glyceraldehyde-3-phosphate dehydrogenase.

## Slide 22
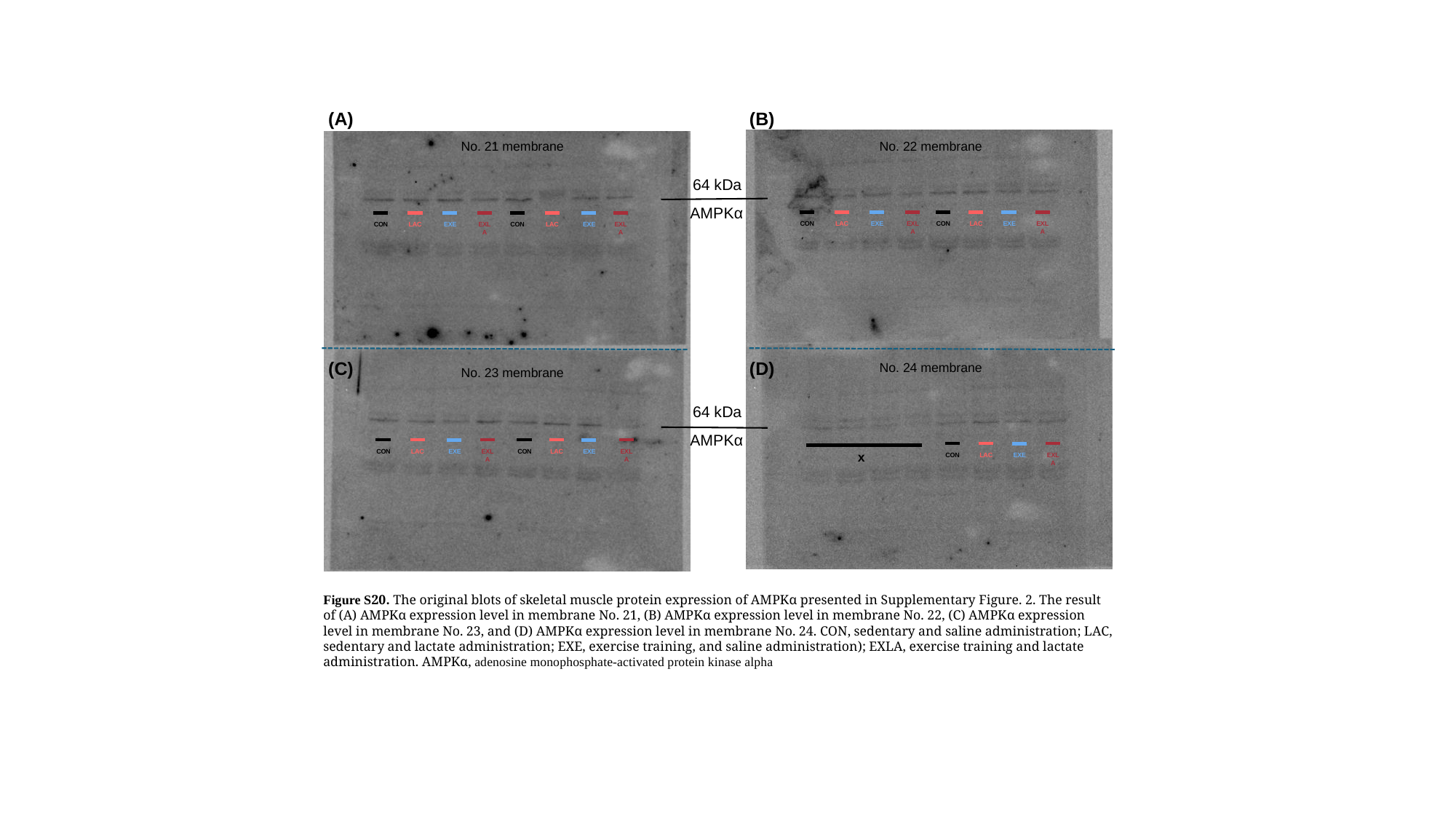

(A)
(B)
No. 21 membrane
No. 22 membrane
64 kDa
AMPKα
CON
LAC
EXE
EXLA
CON
LAC
EXE
EXLA
CON
LAC
EXE
EXLA
CON
LAC
EXE
EXLA
(C)
(D)
No. 24 membrane
No. 23 membrane
64 kDa
AMPKα
CON
LAC
EXE
EXLA
CON
LAC
EXE
EXLA
x
CON
LAC
EXE
EXLA
Figure S20. The original blots of skeletal muscle protein expression of AMPKα presented in Supplementary Figure. 2. The result of (A) AMPKα expression level in membrane No. 21, (B) AMPKα expression level in membrane No. 22, (C) AMPKα expression level in membrane No. 23, and (D) AMPKα expression level in membrane No. 24. CON, sedentary and saline administration; LAC, sedentary and lactate administration; EXE, exercise training, and saline administration); EXLA, exercise training and lactate administration. AMPKα, adenosine monophosphate-activated protein kinase alpha

## Slide 23
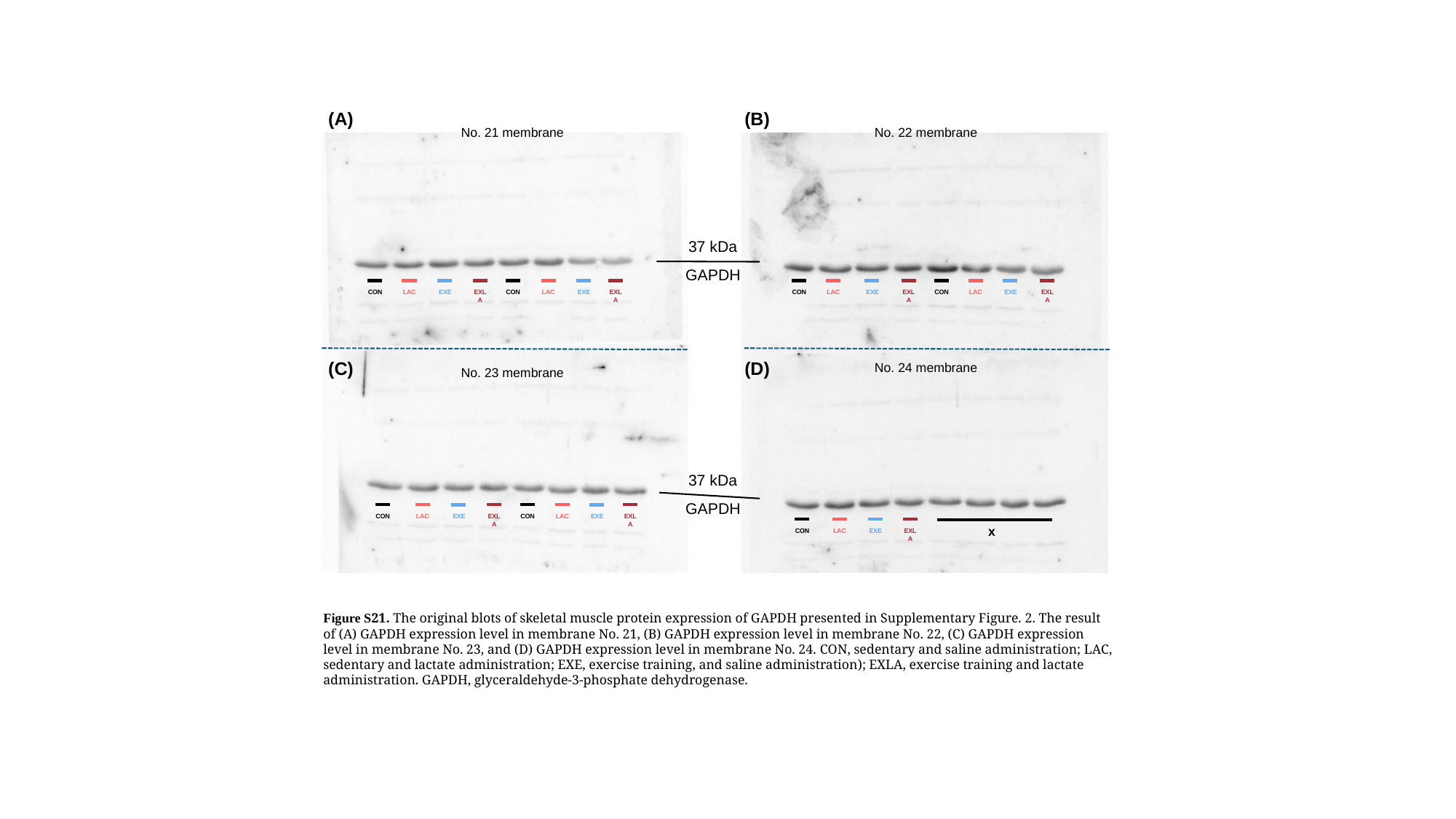

(A)
(B)
No. 21 membrane
No. 22 membrane
37 kDa
GAPDH
CON
LAC
EXE
EXLA
CON
LAC
EXE
EXLA
CON
LAC
EXE
EXLA
CON
LAC
EXE
EXLA
(C)
(D)
No. 24 membrane
No. 23 membrane
37 kDa
GAPDH
CON
LAC
EXE
EXLA
CON
LAC
EXE
EXLA
x
CON
LAC
EXE
EXLA
Figure S21. The original blots of skeletal muscle protein expression of GAPDH presented in Supplementary Figure. 2. The result of (A) GAPDH expression level in membrane No. 21, (B) GAPDH expression level in membrane No. 22, (C) GAPDH expression level in membrane No. 23, and (D) GAPDH expression level in membrane No. 24. CON, sedentary and saline administration; LAC, sedentary and lactate administration; EXE, exercise training, and saline administration); EXLA, exercise training and lactate administration. GAPDH, glyceraldehyde-3-phosphate dehydrogenase.
